# Trends in the prevalence and disability-adjusted life years of eating disorders from 1990 to 2017: results from the Global Burden of Disease Study 2017

**DOI:** 10.1017/S2045796020001055

**Published:** 2020-12-07

**Authors:** Jiayuan Wu, Jie Liu, Shasha Li, Huan Ma, Yufeng Wang

**Affiliations:** 1 Department of Clinical Research, the Affiliated Hospital of Guangdong Medical University, Zhanjiang 524001, Guangdong, China; 2 School of Public Health, Guangdong Medical University, Zhanjiang 524023, Guangdong, China

**Keywords:** Disability-adjusted life years, eating disorders, Global Burden of Disease, prevalence, secular trend

## Abstract

**Aim:**

Eating disorders have increasingly become a public health concern globally. This study aimed to reveal the burden of eating disorders at the global, regional and national levels using the Global Burden of Disease (GBD) Study 2017 data.

**Methods:**

We extracted the age-standardised rates (ASRs) of prevalence and disability-adjusted life years (DALYs) and their 95% uncertainty intervals (UIs) of eating disorders, including anorexia nervosa and bulimia nervosa, between 1990 and 2017 from the GBD 2017 data. The estimated annual percentage changes (EAPCs) were calculated to quantify the secular trends of the burden of eating disorders.

**Results:**

The ASRs of prevalence and the DALYs of eating disorders continuously increased worldwide from 1990 to 2017 by an average of 0.65 (95% UI: 0.59–0.71) and 0.66 (95% UI: 0.60–0.72), respectively. The burden of eating disorders was higher in females than in males, but the increment in ASRs was greater in males than in females over time. In 2017, the highest burden of eating disorders was observed in the high sociodemographic index (SDI) regions, especially Australasia (ASR of prevalence = 807.13, 95% UI: 664.20–982.30; ASR of DALYs = 170.74, 95% UI: 113.43–244.14, per 100 000 population), Western Europe and high-income North America. However, the most significant increment of the burden of eating disorders was observed in East Asia (EAPC for prevalence = 2.23, 95% UI: 2.14–2.32; EAPC for DALYs = 2.22, 95% UI: 2.13–2.31), followed by South Asia. An increasing trend in the burden of eating disorders at the national level was observed among most countries or territories. The countries with the top three highest increasing trends were Equatorial Guinea, Bosnia and Herzegovina and China. Positive associations were found between the burden estimates and the SDI levels in almost all geographic regions during the observed 28-year period. We also found that the human development indexes in 2017 were positively correlated with the EAPCs of the ASRs of prevalence (*ρ* = 0.222, *P* = 0.002) and DALYs (*ρ* = 0.208, *P* = 0.003).

**Conclusion:**

The highest burden of eating disorders remains in the high-income western countries, but an increasing trend was observed globally and in all SDI-quintiles, especially in Asian regions that were highly populous. These results could help governments worldwide formulate suitable medical and health policies for the prevention and early intervention of eating disorders.

## Introduction

Eating disorders, including anorexia nervosa, bulimia nervosa and binge eating disorders (BEDs), are a heterogeneous group of psychiatric illnesses characterised by the development of abnormal eating habit, dysregulation of body weight and overconcern with shape and weight. Unlike other psychiatric illnesses that do not inherently manifest medical complications, eating disorders have considerable influences on all body systems, and confer risk for decreased quality of life, medical complications and function impairment (Westmoreland *et al*., 2016). Anorexia nervosa has the highest mortality rate among all mental health disorders (Gibson *et al*., 2019).

Eating disorders were once considered ‘culture-bound syndromes’ restricted to the western culture because they were initially described in Western Europe and North America (Pike *et al*., 2014). In recent years, eating disorders have been identified as common mental disorders in all continents to a varying extent (Kolar *et al*., 2016; Thomas *et al*., 2016; van Hoeken *et al*., 2016). Although eating disorders have gained increasing attention over the past three decades, accurate information regarding the burden of eating disorders is still lacking. This problem is especially observed at the national level. A basic requirement for the advances in the diagnosis and treatment of eating disorders is a thorough understanding of their epidemiology. Policymakers cannot allocate limited resources and formulate policies rationally without accurate regional and national data on eating disorders.

At present, knowledge of eating disorders risk factors is still lacking. There was no difference in stressful life events (including exposure to physical or sexual abuse, certain family experiences and negative emotions) between people with eating disorders and other mental disorders, suggesting that they are common risk factors for all psychiatric diseases (Hilbert *et al*., 2014). Pursuit of the thin beauty ideal and the resulting body dissatisfaction, dieting and unhealthy weight control behaviours were the specific risk factors of eating disorders (Evans *et al*., 2017). Moreover, genetic factors play an important role in the emergence of eating disorders, with replicated heritability estimates for anorexia nervosa, bulimia nervosa and BED range from 0.48 to 0.74, 0.55 to 0.62, and 0.39 to 0.45, respectively (Bulik *et al*., 2016). These findings support the role of physiological and psychological factors as well as environmental backgrounds in the development of eating disorders.

Traditionally, epidemiological studies on the burden of different diseases have focused on incidence, prevalence and mortality. Although these metrics are essential, they provide a limited assessment when reviewed individually. For instance, the incidence and prevalence metrics only assess harm based on frequency and cannot fully describe the degree and duration of disability, and the mortality metric fails to consider the healthy life loss (GBD 2017 Childhood Cancer Collaborators, 2019). Evidence has demonstrated that eating disorders are responsible for mortality and disability (Erskine *et al*., 2016). In 2012, the Global Burden of Disease (GBD) 2010 was published and the first among the GBD studies to list the disability-adjusted life years (DALYs) as a routine index (Lozano *et al*., 2012; Murray *et al*., 2012). The DALYs at a population level quantify the gap between the present health status and an ideal scenario wherein the entire population is free of disease and has lived to an advanced age. A DALY is equivalent to a one-year loss of healthy life. Health loss due to death and disability is considered by calculating the DALYs through the summation of the years of life lost due to premature death and years lived with disability. The GBD studies have provided considerable information on the burden of eating disorders at the global level that helps to conduct comparisons between regions or countries (Yang *et al*., 2020).

The burden of eating disorders varies in different countries, possibly due to cultural factors and development status. In the general population, there was an 18-fold difference in the reported overall prevalence of eating disorders (range from 0.2 to 3.7%) (Qian *et al*., 2013). However, previous studies attempting to quantify the burden of eating disorders were limited by using point estimates, rather than analysing it as a secular trend. Moreover, no study has explored the associations between the burden of eating disorders and economic health factor in different regions. In this study, we used the GBD 2017 data to reveal the annual changes and trends in the prevalence and the DALYs of eating disorders at the global, regional and national levels. We also determine how the burden of eating disorders is related to the development level as measured by the human development indexes (HDIs).

## Methods

The GBD 2017 provided a comprehensive assessment of the burden of diseases (e.g. incidence, prevalence, mortality and DALYs) for 354 causes in 195 countries and territories from 1990 to 2017. The general methods used in the GBD 2017 were described in detail on the official website (http://www.healthdata.org/gbd/). In the GBD 2017, the countries or territories were classified into five regions, namely, low, low-middle, middle, high-middle and high, in terms of the sociodemographic index (SDI). The world was also geographically divided into 21 regions (e.g. East Asia and Western Europe) to observe the geographic disparities. Moreover, the HDIs of all countries were collected from the World Bank (http://hdr.undp.org/en/content/human-development-index-hdi/).

The estimated age-standardised rates (ASRs) (rates per 100 000 populations) of prevalence and DALYs due to eating disorders from 1990 to 2017 were extracted from the Global Health Data Exchange query tool (http://ghdx.healthdata.org/gbd-results-tool/). The ASRs and the 95% uncertainty intervals (UIs) were calculated based on the GBD 2017 global age-standard population (GBD 2017 Disease and Injury Incidence and Prevalence Collaborators, 2018). In the GBD 2017, eating disorders are subdivided into two categories: anorexia nervosa and bulimia nervosa. We also described the prevalence and the DALYs of these two types of eating disorders at the global, regional and national levels. Eating disorders were defined in accordance with the Diagnostic and Statistical Manual of Mental Disorder (DSM) and the International Classification of Disease (ICD) criteria. Different versions of DSM (DSM-III, DSM-III-R, DSM-IV, DSM-IV-TR and DSM-5) and ICD (ICD-9 and ICD-10) were accepted. The GBD 2017 also assumed no case of eating disorders prior to age 5 or after 50. These settings were in line with the corresponding cause of death model for eating disorders. The gender-specific crude rates and the 95% UIs were used to compare the burden of eating disorders between age groups: 5–9, 10–14, 15–19, 20–24, 25–29, 30–34, 35–39, 40–44 and 45–49.

The annual ASRs and the corresponding estimated annual percentage changes (EAPCs) were used to quantify the secular trends of prevalence and DALYs due to eating disorders. The ASR was calculated by summarising the products of age-specific rates (*α_i_*, where *i* denotes the *i*th age class) and number of cases (or weight; *w_i_*) in the same age subgroup *i* of the selected reference standard population, and then dividing it by the sum of the standard population weights (Zhou *et al*., 2019). The age-standardised population in the GBD was calculated using the GBD world population age standard. The specific explanations about age classes and their weights were provided in the online Supplementary Appendix of the published paper by GBD 2017 Mortality Collaborators (GBD 2017 Mortality Collaborators, 2018).
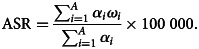
The EAPC was commonly used to reflect the variation tendency of ASRs over a specified interval. Accordingly, a regression line was fitted to the natural logarithm of the rates: *y* = *α* + *β*x + *ɛ*, where *y* = ln (ASR) and *x* = calendar year. The EAPC was calculated as 100 × (exp(*β*) − 1), and its 95% UI was obtained from the linear regression model (Liu *et al*., 2019; He *et al*., 2020). An increasing ASR trend was observed when EAPC > 0 and 95% UI > 0. By contrast, a decreasing ASR trend was identified when EAPC estimation <0 and 95% UI did not exceed 0. Otherwise, ASR was regarded as stable over time.

To explore the influential factors for EAPCs, we assessed the associations of EAPCs with the ASRs in 1990 and the HDIs in 2017 at the national level through scatter plots and Pearson's correlation analysis. The ASRs of eating disorders in 1990 indicate the disease reservoir at baseline and the HDIs in 2017 can serve as an alternative index for the quality and availability of health care in each country.

All statistical procedures were performed with the R program (version 3.5.3, R core team). *p* value < 0.05 was considered statistically significant.

## Results

### Burden of eating disorders at the global level

**Fig. 1.**
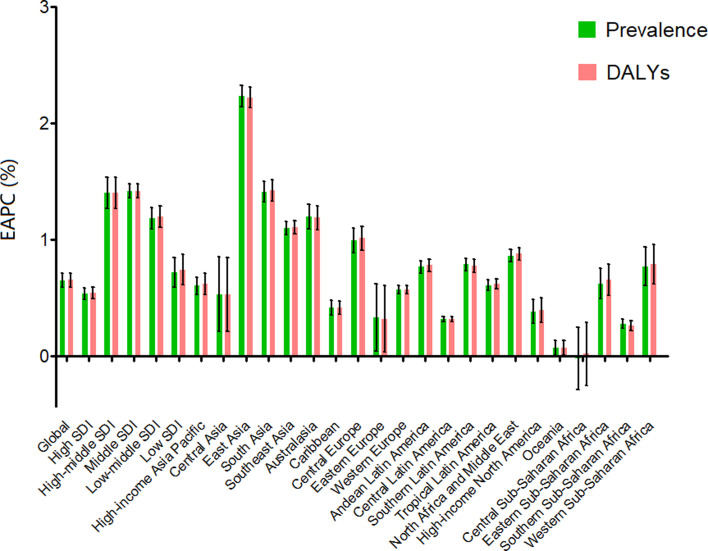
EAPC of prevalence and DALYs for eating disorders at the global and regional levels. EAPC, estimated annual percentage change; DALYs, disability-adjusted life years.

The global burden of eating disorders and their trends are listed in Table 1. The global age-standardised prevalence rates of eating disorders gradually increased from 172.53 (95% UI: 138.22–211.82) in 1990 to 203.20 (95% UI: 162.34–250.75) in 2017 per 100 000 population by an average of 0.65 (95% UI: 0.59–0.71; Fig. 1). The same increased trend was found in the age-standardised DALY rates of eating disorders from 35.75 (95% UI: 23.62–53.12) in 1990 to 43.36 (95% UI: 27.88–62.85) in 2017 per 100 000 population, indicating an increasing rate of 0.66 per year (95% UI: 0.60–0.72; Fig. 1).

**Table 1.** Age-standardised rates of prevalence and disability-adjusted life-years of eating disorders in 2017 and their temporal trend from 1990 to 2017 at global and regional levels

	Prevalence (95% UI)			DALYs (95% UI)		
	ASR in 1990 (per 1 00 000 population)	ASR in 2017 (per 1 00 000 population)	EAPC (1990–2017)	ASR in 1990 (per 1 00 000 population)	ASR in 2017 (per 1 00 000 population)	EAPC (1990–2017)
Global	172.53 (138.22–211.82)	203.20 (162.34–250.75)	0.65 (0.59–0.71)	36.75 (23.62–53.12)	43.36 (27.88–62.85)	0.66 (0.60–0.72)
Sex						
Male	103.84 (80.97–131.02)	124.08 (96.21–156.44)	0.71 (0.63–0.78)	22.32 (14.20–32.91)	26.66 (16.85–39.10)	0.70 (0.63–0.78)
Female	243.15 (197.05–298.13)	284.20 (230.57–349.30)	0.62 (0.56–0.68)	51.57 (33.26–74.57)	60.46 (39.23–87.62)	0.63 (0.58–0.68)
Aetiology						
Anorexia nervosa	39.33 (29.86–50.13)	43.87 (33.10–56.52)	0.44 (0.40–0.48)	8.49 (5.29–12.55)	9.51 (5.91–14.09)	0.45 (0.42–0.49)
Bulimia nervosa	134.19 (102.00–171.29)	160.25 (121.55–204.57)	0.71 (0.64–0.77)	28.26 (17.90–42.21)	33.85 (21.34–50.45)	0.72 (0.65–0.78)
Socio-demographic index						
High SDI	391.80 (319.21–476.14)	449.20 (365.14–548.76)	0.54 (0.49–0.59)	83.58 (54.47–120.27)	96.08 (62.90–138.97)	0.54 (0.49–0.59)
High-middle SDI	149.71 (118.07–184.83)	212.38 (168.49–262.67)	1.40 (1.27–1.54)	31.94 (20.45–46.63)	45.39 (29.07–66.49)	1.40 (1.27–1.53)
Middle SDI	121.57 (96.33–149.60)	176.65 (140.10–218.16)	1.42 (1.36–1.48)	26.03 (16.56–37.94)	37.79 (24.17–55.01)	1.42 (1.36–1.48)
Low-middle SDI	116.74 (92.25–144.69)	156.96 (123.42–194.28)	1.18 (1.09–1.27)	24.72 (15.77–36.17)	33.35 (21.08–48.53)	1.20 (1.10–1.29)
Low SDI	98.94 (77.49–122.79)	120.00 (94.58–149.07)	0.72 (0.60–0.85)	20.88 (13.17–30.46)	25.47 (16.18–37.32)	0.74 (0.62–0.87)
Region						
High-income Asia Pacific	338.74 (275.35–417.68)	404.82 (328.52–500.29)	0.61 (0.53–0.68)	73.20 (47.38–105.55)	88.18 (56.97–127.70)	0.62 (0.53–0.71)
Central Asia	150.94 (118.90–188.29)	165.13 (131.03–205.74)	0.53 (0.21–0.86)	32.32 (20.86–47.44)	35.39 (22.59–52.43)	0.53 (0.21–0.85)
East Asia	84.48 (65.96–104.18)	150.15 (118.79–185.72)	2.23 (2.14–2.32)	18.28 (11.7–26.84)	32.42 (20.51–48.03)	2.22 (2.13–2.31)
South Asia	105.28 (82.98–130.40)	152.08 (119.33–188.39)	1.41 (1.32–1.50)	22.30 (14.14–32.76)	32.3 (20.55–47.01)	1.42 (1.34–1.51)
Southeast Asia	102.47 (81.11–126.44)	140.88 (112.00–173.55)	1.10 (1.04–1.16)	21.94 (13.92–32.12)	30.20 (19.24–44.23)	1.11 (1.05–1.16)
Australasia	620.95 (496.19–773.29)	807.13 (664.20–982.30)	1.20 (1.09–1.30)	131.58 (85.74–193.54)	170.74 (113.43–244.14)	1.19 (1.09–1.29)
Caribbean	230.07 (182.09–289.53)	249.75 (198.41–314.41)	0.42 (0.36–0.48)	49.04 (31.43–71.70)	53.26 (34.26–78.97)	0.42 (0.36–0.48)
Central Europe	165.24 (130.61–205.72)	205.03 (162.90–254.32)	0.99 (0.89–1.10)	35.26 (22.46–51.63)	43.93 (27.86–64.75)	1.01 (0.91–1.12)
Eastern Europe	195.18 (153.66–242.58)	203.75 (159.89–254.93)	0.34 (0.05–0.62)	41.55 (26.35–60.67)	43.36 (27.69–63.52)	0.32 (0.04–0.61)
Western Europe	455.00 (373.25–551.26)	525.20 (428.64–640.61)	0.57 (0.54–0.61)	97.16 (63.22–140.56)	112.27 (73.12–162.79)	0.57 (0.54–0.61)
Andean Latin America	280.76 (220.08–356.14)	341.84 (269.48–434.28)	0.77 (0.72–0.82)	59.74 (37.76–89.95)	72.93 (46.49–107.52)	0.78 (0.73–0.83)
Central Latin America	248.01 (198.20–310.23)	270.77 (216.36–337.71)	0.32 (0.30–0.34)	53.01 (33.85–78.16)	57.95 (36.65–85.36)	0.32 (0.30–0.34)
Southern Latin America	297.05 (239.20–369.07)	374.15 (301.60–463.47)	0.79 (0.74–0.84)	63.14 (40.31–91.68)	79.38 (50.49–116.75)	0.78 (0.72–0.83)
Tropical Latin America	225.15 (178.34–282.92)	263.99 (210.00–329.82)	0.61 (0.57–0.65)	47.63 (30.58–69.78)	56.04 (35.98–81.74)	0.62 (0.58–0.67)
North Africa and Middle East	168.74 (134.38–209.36)	205.72 (162.61–254.43)	0.86 (0.81–0.92)	35.54 (22.74–51.68)	43.53 (27.78–63.72)	0.88 (0.83–0.93)
High-income North America	425.45 (342.39–523.43)	463.45 (373.25–570.99)	0.38 (0.28–0.49)	89.98 (58.66–130.21)	98.36 (63.78–144.38)	0.40 (0.29–0.50)
Oceania	102.24 (80.34–127.38)	108.61 (84.04–134.55)	0.08 (0.01–0.14)	21.79 (13.74–32.01)	23.15 (14.54–33.66)	0.08 (0.01–0.14)
Central Sub-Saharan Africa	116.53 (90.83–145.04)	116.21 (90.79–145.53)	−0.02 (−0.29 to 0.25)	24.36 (15.63–35.40)	24.58 (15.59–35.95)	0.02 (−0.25 to 0.29)
Eastern Sub-Saharan Africa	97.56 (76.46–122.07)	113.72 (88.58–141.25)	0.63 (0.49–0.76)	20.64 (13.19–30.16)	24.22 (15.39–35.49)	0.66 (0.52–0.79)
Southern Sub-Saharan Africa	179.38 (141.46–222.29)	189.87 (150.18–236.37)	0.28 (0.24–0.32)	37.97 (24.31–55.56)	40.08 (25.32–58.64)	0.26 (0.22–0.31)
Western Sub-Saharan Africa	119.92 (94.64–149.50)	140.28 (110.07–174.79)	0.77 (0.61–0.94)	25.26 (16.02–37.23)	29.72 (18.99–43.36)	0.79 (0.62–0.96)

DALYs, disability-adjusted life-years; ASR, age-standardised rate; EAPC, estimated annual percentage change; UI, uncertainty interval.

The global burdens of anorexia nervosa and bulimia nervosa and their trends are listed in online Supplementary Tables 1, 2, respectively. The global age-standardised prevalence rates of anorexia nervosa increased from 39.33 (95% UI: 29.86–50.13) in 1990 to 43.87 (95% UI: 33.10–56.52) in 2017 per 100 000 population with the EAPC being 0.44 (95% UI: 0.40–0.48; online Supplementary Fig. 1A). The global age-standardised DALY rates of anorexia nervosa increased from 8.49 (95% UI: 5.29–12.55) in 1990 to 9.51 (95% UI: 5.91–14.09) in 2017 per 100 000 population by an average of 0.45 (95% UI: 0.42–0.49; online Supplementary Fig. 1A). The global age-standardised prevalence rates of bulimia nervosa increased from 134.19 (95% UI: 101.98–171.29) in 1990 to 160.25 (95% UI: 121.55–204.57) in 2017 per 100 000 population at 0.71 per year (95% UI: 0.64–0.77). The global age-standardised DALY rates of bulimia nervosa increased from 28.26 (95% UI: 17.90–42.21) in 1990 to 33.85 (95% UI: 21.34–50.45) in 2017 per 100 000 population at 0.72 per year (95% UI: 0.65–0.78; online Supplementary Fig. 1B).

Women had a higher annual ASR than men in terms of the prevalence (online Supplementary Fig. 2A) and the DALYs (online Supplementary Fig. 2B) of eating disorders and its subtypes during the observed period. However, males showed a greater increment in the age-standardised prevalence and DALY rates than females over time concerning eating disorders (Table 1), anorexia nervosa (online Supplementary Table 1) and bulimia nervosa (online Supplementary Table 2).

The high-SDI region had the highest age-standardised prevalence and DALY rates in 2017 in terms of eating disorders and its subtypes. An overall increasing trend in the age-standardised prevalence and DALY rates was observed in all SDI regions from 1990 to 2017. The greatest increasing trends of these indexes were found in the high-middle and middle SDI regions in terms of eating disorders (Fig. 1), anorexia nervosa (online Supplementary Fig. 1A) and bulimia nervosa (online Supplementary Fig. 1B).

**Fig. 2.**
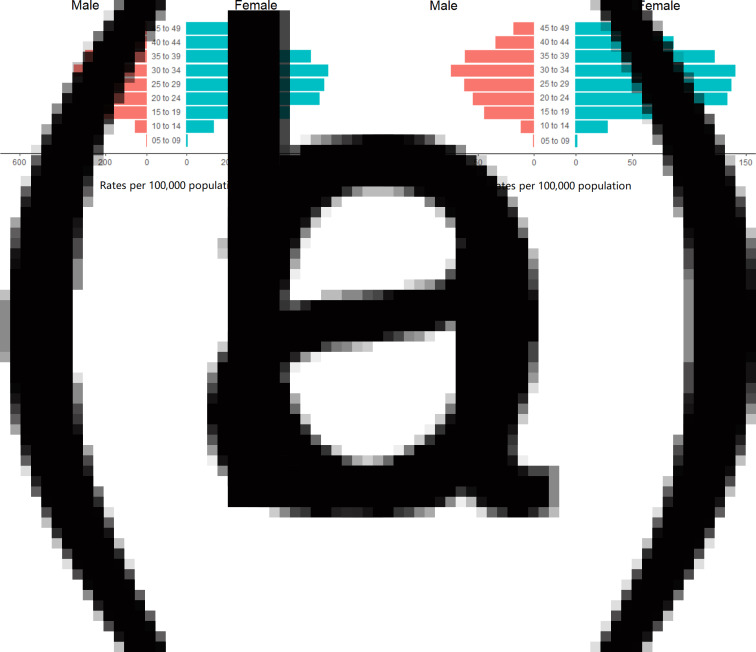
Prevalence rates (a) and DALY rates (b) of eating disorders in different age groups globally. DALYs, disability-adjusted life years.

We also analysed the crude rates of prevalence and the DALY rates for males and females in different age groups concerning eating disorders (Fig. 2), anorexia nervosa (online Supplementary Figs 3A, B) and bulimia nervosa (online Supplementary Figs 3C, D). The crude rates of prevalence and the DALY rates were higher for females than for males in all age groups. The highest crude rates of prevalence and DALY rates were observed among those aged 15–39 years for eating disorders and anorexia nervosa, and among those aged 15–44 years for bulimia nervosa.

### Burden of eating disorders at the regional level

The burden of eating disorders and their trends at the regional level are listed in Table 1. In 2017, Australasia had the highest age-standardised prevalence (807.13, 95% UI: 664.20–982.30, per 100 000 population) and DALY rates (170.74, 95% UI: 113.43–244.14, per 100 000 population), followed by Western Europe. As shown in Fig. 1, the ASRs of prevalence and DALYs of eating disorders increased in all geographic regions in the observed period, except for central Sub-Saharan Africa. The most significant increase of ASRs was detected in East Asia (EAPC for prevalence = 2.23, 95% UI: 2.14–2.32; EAPC for DALYs = 2.22, 95% UI: 2.13–2.31), followed by South Asia (EAPC for prevalence = 1.41, 95% UI: 1.32–1.50; EAPC for DALYs = 1.42, 95% UI: 1.34–1.51).

The burdens of anorexia nervosa and bulimia nervosa and their trends at the regional level are listed in online Supplementary Tables 1, 2, respectively. In 2017, Western Europe had the highest age-standardised prevalence (134.53, 95% UI: 102.65–172.92, per 100 000 population) and DALY rates (29.39, 95% UI: 18.47–43.55, per 100 000 population) for anorexia nervosa. In the same year, Australasia had the highest age-standardised prevalence (692.78, 95% UI: 552.74–855.61, per 100 000 population) and DALY rates (145.51, 95% UI: 94.72–212.43, per 100 000 population) for bulimia nervosa. Similar to eating disorders, the age-standardised prevalence and DALY rates of anorexia nervosa and bulimia nervosa increased in all 21 geographic regions, except for central Sub-Saharan Africa. East Asia had the most significant increment of ASRs for anorexia nervosa (EAPC for prevalence = 2.06, 95% UI: 2.00–2.11; EAPC for DALYs = 2.09, 95% UI: 2.04–2.15; online Supplementary Fig. 1A) and bulimia nervosa (EAPC for prevalence = 2.25, 95% UI: 2.15–2.36; EAPC for DALYs = 2.27, 95% UI: 2.16–2.38; online Supplementary Fig. 1B), followed by South Asia.

### Burden of eating disorders at the national level

**Fig. 3.**
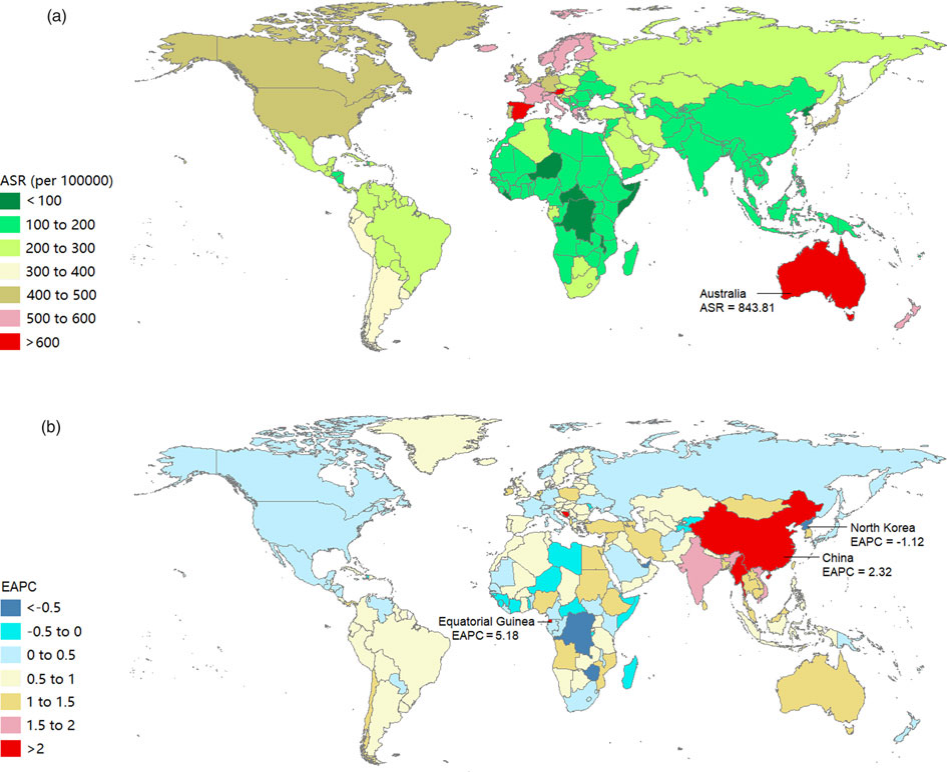
Age-standardised rates of prevalence (a) and DALYs (b) of eating disorders by SDI from 1990 to 2017, and expected value-based SDI. The black line represents the average expected relationship between SDI and prevalence (a) or DALYs (b) of eating disorders based on values from all regions from 1990 to 2017. DALYs, disability-adjusted life years; SDI, sociodemographic index.

**Fig. 4.**
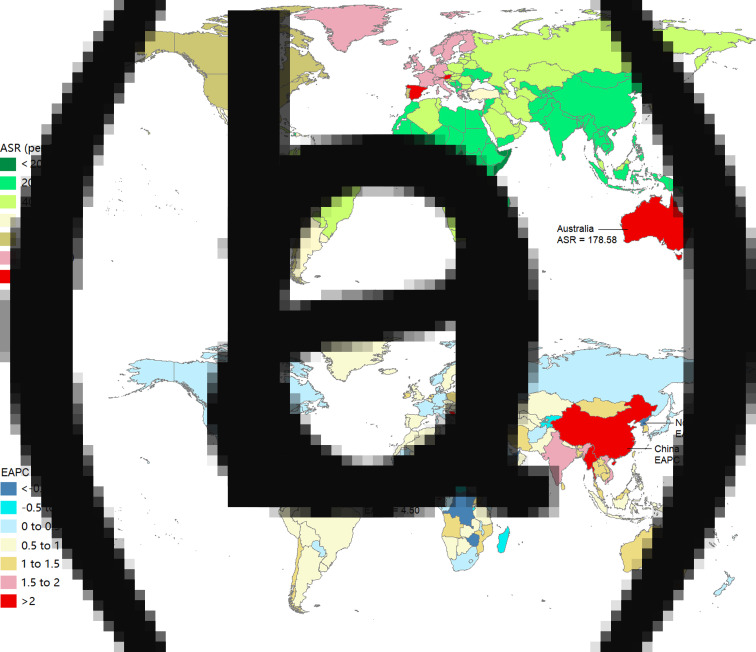
Age-standardised prevalence rates in 2017 (a) and the estimated annual percentage change of age-standardised prevalence rates from 1990 to 2017 (b) of eating disorders in 195 countries or territories.

The burden of eating disorders and their trends at the national level are listed in online Supplementary Table 3. In 2017, the three countries with the highest ASRs of prevalence and DALYs due to eating disorders were Australia, Luxembourg and Spain (Figs 3a, 4a). We observed an increasing trend in the age-standardised prevalence and DALY rates of eating disorders among 164 and 166 countries or territories, respectively. From 1990 to 2017, the top three countries with increasing trends in the ASRs of prevalence and DALYs due to eating disorders were Equatorial Guinea, Bosnia and Herzegovina and China (Figs 3b, 4b).

The burdens of anorexia nervosa and bulimia nervosa and their trends at national level are listed in online Supplementary Tables 4, 5, respectively. In 2017, Luxembourg, Spain and Finland were the three countries with the highest ASRs of prevalence and DALYs of anorexia nervosa (online Supplementary Figs 4A, 5A), and Australia, Luxembourg and Spain were the three countries with the highest ASRs of prevalence and DALYs of bulimia nervosa (online Supplementary Figs 6A, 7A). During the observation period, we observed increasing trends in the age-standardised prevalence rates among 164 and 165 countries or territories in terms of anorexia nervosa and bulimia nervosa, respectively. Likewise, we observed increasing trends in the age-standardised DALY rates among 163 and 167 countries or territories in terms of anorexia nervosa and bulimia nervosa, respectively. The top three countries with increasing trends in the ASRs of prevalence and DALYs for anorexia nervosa were Equatorial Guinea, Bosnia and Herzegovina and China (online Supplementary Figs 4B, 5B). These three countries also had the highest EAPCs of age-standardised prevalence and DALY rates for bulimia nervosa (online Supplementary Figs 6B, 7B).

### Relationship between burden estimates of eating disorders and SDI level

**Fig. 5.**
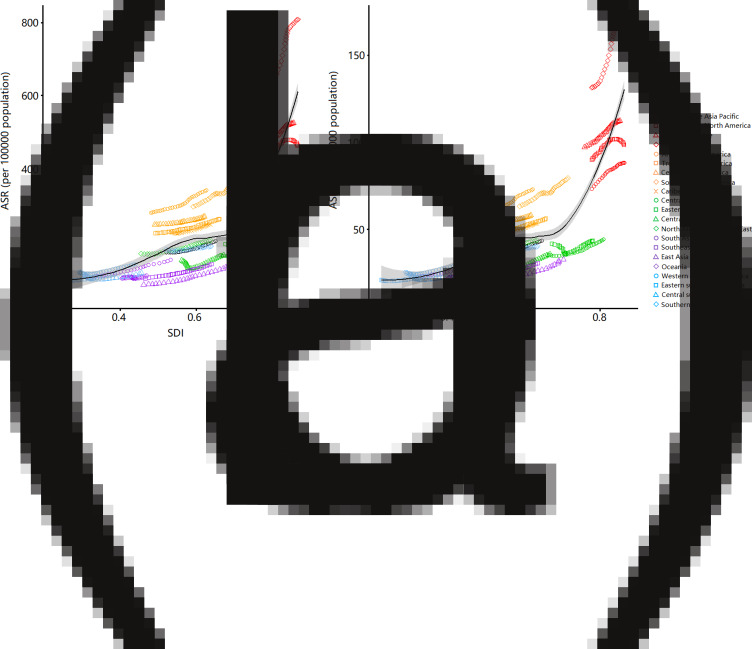
Age-standardised DALY rates in 2017 (a) and the estimated annual percentage change of age-standardised DALY rates from 1990 to 2017 (b) of eating disorders in 195 countries or territories. DALYs, disability-adjusted life years.

We illustrated the associations between the burden estimates of eating disorders and the SDI levels for each geographic region from 1990 to 2017 (Fig. 5). The SDIs increased in all 21 geographic regions over the 28 years observation period. Positive associations were observed between burden estimates and the SDI level in almost all regions. Burden estimates tended to be slightly raised with increasing SDIs when the SDIs were under 0.65. By contrast, burden estimates experienced a sharp increase with increasing SDIs when the SDIs were over 0.65.

### The influential factors for EAPC

**Fig. 6.**
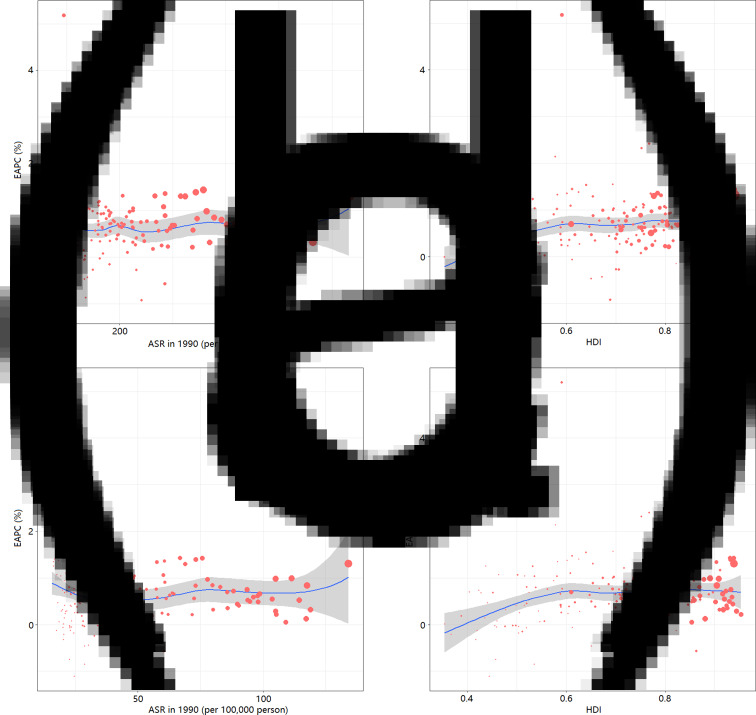
Correlation between the EAPC of age-standardised prevalence rates and the age-standardised prevalence rates in 1990 (a); the EAPC of age-standardised prevalence rates and the HDIs in 2017 (b); the EAPC of age-standardised DALY rates and the age-standardised DALY rates in 1990 (c); and the EAPC of age-standardised DALY rates and the HDIs in 2017 (d). The size of circle represents the age-standardised rates of prevalence or DALYs in this country or territory in 2017. DALYs, disability-adjusted life years; EAPC, estimated annual percentage change; HDI, human development index.

As shown in Figs 6b, d, a positive correlation was detected between the HDIs in 2017 and the EAPCs of age-standardised prevalence (*ρ* = 0.222, *P* = 0.002) and DALY rates (*ρ* = 0.208, *P* = 0.003), respectively. The ASRs in 1990 did not seem to affect the EAPCs with respect to the prevalence (*ρ* = −0.032, *P* = 0.661, Fig. 6a) and DALYs rates (*ρ* = −0.040, *P* = 0.581, Fig. 6c) of eating disorders.

## Discussion

To our knowledge, this is the first study to explore the burden estimates of eating disorders at global, regional and national levels based on the GBD 2017 data. The burden of eating disorders, as measured by prevalence and DALYs, continuously increased globally from 1990 to 2017, and in all regions regardless of the SDI. Of note, the burden of eating disorders raised with the increased SDI at regional level and the highest burden was observed in the high SDI regions, especially Australasia, Western Europe and high-income North America. However, the most significant increment was observed in East Asia and South Asia.

The continuously increasing trend of the global burden of eating disorders could be attributed to the improved awareness of mental disorders. A longitudinal study conducted in Denmark (Steinhausen *et al*., 2015) showed that the incidence rates of eating disorders increased from 1995 to 2010. Simultaneously, an increase in rates was also observed in all mental disorders. The increase in eating disorders did not exceed this general trend. Another study in China also revealed that economic growth was significantly associated with the deterioration in mental health in the 21st century, and the increment in eating disorders diagnosis was consistent with the increasing rate in all psychiatric diagnoses (Wang *et al*., 2019). The changes of the diagnostic practices could also provide an understanding of the true prevalence and burden of eating disorders. The criteria for anorexia nervosa and bulimia nervosa were broadened in the DSM-5 criteria (Smink *et al*., 2014). The changes introduced in the latest version could capture more individuals within these two specified types of eating disorders and reduce rates of other or unspecified diagnoses (Vo *et al*., 2017).

In line with our expectations, females demonstrated higher levels of burden of eating disorders than males. Eating disorders were once regarded as the most gendered psychiatric illnesses because women comprised most of the public health burden associated with eating disorders. However, recent studies showed that males also account for considerable eating disorders cases (Mitchison *et al*., 2014; Mitchison and Mond, 2015). Thus, suggesting that eating disorders were uncommon among males was no longer appropriate. In fact, the burden of male eating disorders in much of the previous studies is a gross underestimate (Dakanalis *et al*., 2016). Eating disorders in males were ignored until recently for several reasons. First, the omission of males from considerable research on eating disorders hindered the assessment of the male patients (Murray *et al*., 2017). Second, the female-biased diagnostic criteria led to a declined diagnostic efficiency in males (Robinson *et al*., 2013). Third, eating disorders were presented with different symptoms in males than in females. Fourth, many male patients concealed their diseases and did not seek treatment due to the stigma for having a ‘female disease’. Thus, obtaining an accurate prevalence on the number of men with these diseases was difficult at a population level. To address this gap, the criteria for eating disorders in the DSM-5 were recently broadened to make them less gendered. The burden of eating disorders, especially bulimia nervosa, in males was increasing faster than in females. This situation could be attributed to the increasing number of studies on the prevalence of eating disorders in males. However, a clear lag was still observed in the screening, assessment, classification and treatment specific to male concerns that were critically in need of advancement. Moreover, we observed that the gender difference in the burden of bulimia nervosa was much smaller than that in anorexia nervosa (online Supplementary Fig. 3), which could be partly explained by the difference in hormone level. In both experimental animals and humans, oestrogen reduces food intake, whereas testosterone may enhance appetite (Hirschberg, 2012). It has been proposed that sex hormones involve in the pathophysiology of eating disorders (Culbert *et al*., 2016). For example, androgens may promote bulimia by stimulating craving for food and/or reducing impulse control, and the antiandrogenic treatment attenuates bulimic behaviours (Castellini *et al*., 2019).

Globally, eating disorders were more common in high-income, industrialised western world. However, the burden of eating disorders was increasing more significantly in Asian countries, especially in China and India. The proceeding industrialisation, modernisation and social change in these countries with large populations were suspected as catalysts for increasing incidence rates of eating disorders. For instance, the rapid economic development that China has experienced since the 1990s has increased people's exposure to various risk factors of eating disorders, leading to an increment in eating disorders burden (Wu *et al*., 2020). Over the last two decades, the detection rate of eating disorders in China has increased with the improvement of diagnostic accuracy (Zheng *et al*., 2019). Although eating disorders contributed to a relatively small proportion to the overall health burden at the country level in these Asian countries, their contributions to the health burdens caused by eating disorders were proportionally and substantively large at the global level (Erskine *et al*., 2017). These areas remained relatively neglected within the scope of eating disorders research, highlighting a critical scientific gap and opportunity. The differences between regions or countries could be attributed to differences in cultural factors, including addictions (e.g. Internet addiction), distorted body images influenced by the media and exaggerated weight loss diets (Hammerle *et al*., 2016). These differences could also be due to distinct genetic backgrounds and dietary behaviours influenced by the environment, including diet and intestinal microbiota (Lozupone *et al*., 2012; Chong *et al*., 2015). Indeed, risk factors for eating disorders could be observed on many levels and generations.

The current data on the epidemiology of eating disorders outside Euro-American context were still sparse, with little to no population coverage in many countries. The GBD 2017 estimation showed that the prevalence of eating disorders in Latin America was higher than that in Asia with similar SDIs. This situation could be due to the stronger exposure to western culture in Latin American population than in Asian population. However, the burden of eating disorders was lower in Latin America than in western countries, which could be due to the different body ideal of Latinas and Latinos that idealises a ‘curvier’ shape and higher body weight than in western countries (Warren *et al*., 2013). The Latina identity and body image could serve as a protective factor for eating disorders when exposed to thin-ideal media (Schooler and Daniels, 2014). A similar picture was observed in Africa. A national survey in South Africa revealed that more black African women were happy with their current weight and fewer attempted to lose weight despite a mean BMI of 29.0 kg/m^2^ (Morris and Szabo, 2013). Given the widespread use of television, film and Internet on the African and Latin American continents, the exposure to western cultures and the risk of eating disorders were increasing, particularly in highly educated persons (van Hoeken *et al*., 2016).

We also found that the variations of ASRs from 1990 to 2017 were significantly and positively associated with the HDIs in 2017. The burden of eating disorders was more likely to increase in countries with high HDIs in 2017. This result could be explained by the following reasons: (1) people in countries with high socioeconomic levels were facing more social pressure and prone to have psychological problems; and (2) the wide spread of the electronic media in countries with high socioeconomic situation put people at great risk for eating disorders. Moreover, countries with low socioeconomic developments could possibly pay less attention to eating disorders, leading to more patients not being diagnosed in time. However, the amplitude in ASR variations showed a steady trend when the HDIs in 2017 exceeded 0.6. This result could be explained by the following reasons: (1) adequate medical and healthcare resources could reduce the burden of eating disorders; and (2) people were likely to develop healthy lifestyles and seek medical treatment.

Given the size and cost of the problem, the research activity and funding concerning eating disorders were lower than other mental disorders. The governments of countries with high burden of eating disorders should support relevant research and take steps to effectively reduce the burden of these disorders. The underlying mechanisms of eating disorders were still unclear. The interactions between genetic and environmental factors at a crucial period in development could possibly increase the complexity of modelling these disorders (Hilbert *et al*., 2014). Prospective longitudinal studies with large sample and sufficient power were significant to reveal the interactions of a range of risk factors (e.g. biological, environmental and psychological) in understanding the pathophysiology and natural history of eating disorders. Eating disorders were even vastly misunderstood by the general population for a long time. To solve this problem, the Academy of Eating Disorders recently published ‘nine truths about eating disorders’ to help the public understand these illnesses (Schaumberg *et al*., 2017). Early intervention was also considered helpful in improving outcomes. Therefore, rapid mediation and psychological interventions should be provided to people who are already suffering eating disorders rather than watchful waiting is essential.

Some limitations were unavoidable in this study due to the restrictions of the GBD 2017 database. In brief, the GBD estimations were reconstructed through an algorithm based on a large number of sources with different qualities, which (to some degree) could be deviant with regard to the actual data. For example, extremely sparse data resulted in inaccurate estimates for some underdeveloped regions, such as Africa and Latin America. Moreover, the GBD 2017 failed to include two other common types of eating disorders, namely, BED and other specified feeding and eating disorders (OSFEDS). These two types of eating disorders could contribute to a large proportion of related cases. The estimated burden in GBD 2017 was likely underestimated. As the GBD was set for continual updates, future GBD studies could consider BED and OSFEDS to provide an opportunity to represent the burden of eating disorders accurately.

## Conclusion

This study used the GBD 2017 database to provide a comprehensive description of the global burden of eating disorders. Although the highest burden of eating disorders remains in the high-income western countries, an increasing trend was also observed globally and in all SDI-quintiles, especially in Asian regions that are highly populous. Our findings highlight eating disorders as a global health problem and showed that variations in burden should be investigated by considering by region, country, sex and year when setting global health objectives. Our results could help governments around the world to understand their countries' burdens of eating disorders and formulate suitable medical and health policies for the prevention and early intervention of eating disorders.

## Data Availability

The data support the findings of this study and are available from the corresponding author upon reasonable request.

## Supplementary material

For supplementary material accompanying this paper visit https://doi.org/10.1017/S2045796020001055. click here to view supplementary material

## References

[ref1] Bulik CM, Kleiman SC and Yilmaz Z (2016) Genetic epidemiology of eating disorders. Current Opinion in Psychiatry 29, 383–388. 27532941 10.1097/YCO.0000000000000275 PMC5356465

[ref2] Castellini G, Lelli L, Cassioli E and Ricca V (2019) Relationships between eating disorder psychopathology, sexual hormones and sexual behaviours. Molecular and Cellular endocrinology 497, 110429. 31026479 10.1016/j.mce.2019.04.009

[ref3] Chong CW, Ahmad AF, Lim YAL, Teh CSJ, Yap IKS, Lee SC, Chin YT, Loke P and Chua KH (2015) Effect of ethnicity and socioeconomic variation to the gut microbiota composition among pre-adolescent in Malaysia. Scientific Reports 5, 13338. 26290472 10.1038/srep13338 PMC4542465

[ref4] Culbert KM, Racine SE and Klump KL (2016) Hormonal factors and disturbances in eating disorders. Current Psychiatry Reports 18, 65. 27222139 10.1007/s11920-016-0701-6

[ref5] Dakanalis A, Pla-Sanjuanelo J, Caslini M, Volpato C, Riva G, Clerici M and Carrà G (2016) Predicting onset and maintenance of men's eating disorders. International Journal of Clinical and Health Psychology 16, 247–255. 30487868 10.1016/j.ijchp.2016.05.002 PMC6225078

[ref6] Erskine HE, Whiteford HA and Pike KM (2016) The global burden of eating disorders. Current Opinion in Psychiatry 29, 346–353. 27532942 10.1097/YCO.0000000000000276

[ref7] Erskine HE, Baxter AJ, Patton G, Moffitt TE, Patel V, Whiteford HA and Scott JG (2017) The global coverage of prevalence data for mental disorders in children and adolescents. Epidemiology and Psychiatric Sciences 26, 395–402. 26786507 10.1017/S2045796015001158 PMC6998634

[ref8] Evans EH, Adamson AJ, Basterfield L, Le Couteur A, Reilly JK, Reilly JJ and Parkinson KN (2017) Risk factors for eating disorder symptoms at 12 years of age: a 6-year longitudinal cohort study. Appetite 108, 12–20. 27612559 10.1016/j.appet.2016.09.005 PMC5152119

[ref9] GBD (2017) Childhood Cancer Collaborators (2019) The global burden of childhood and adolescent cancer in 2017: an analysis of the Global Burden of Disease Study 2017. Lancet Oncology 20, 1211–1225. 31371206 10.1016/S1470-2045(19)30339-0 PMC6722045

[ref10] GBD 2017 Disease and Injury Incidence and Prevalence Collaborators (2018) Global, regional, and national incidence, prevalence, and years lived with disability for 354 diseases and injuries for 195 countries and territories, 1990–2017: a systematic analysis for the Global Burden of Disease Study 2017. Lancet (London, England) 392, 1789–1858. 10.1016/S0140-6736(18)32279-7 PMC6227754 30496104

[ref11] GBD 2017 Mortality Collaborators (2018) Global, regional, and national age-sex-specific mortality and life expectancy, 1950–2017: a systematic analysis for the Global Burden of Disease Study 2017. Lancet (London, England) 392, 1684–1735. 10.1016/S0140-6736(18)31891-9 PMC6227504 30496102

[ref12] Gibson D, Workman C and Mehler PS (2019) Medica complications of anorexia nervosa and bulimia nervosa. Psychiatric Clinics of North America 42, 263–274. 10.1016/j.psc.2019.01.009 31046928

[ref13] Hammerle F, Huss M, Ernst V and Bürger A (2016) Thinking dimensional: prevalence of DSM-5 early adolescent full syndrome, partial and subthreshold eating disorders in a cross-sectional survey in German schools. BMJ Open 6, e010843. 10.1136/bmjopen-2015-010843 PMC4861098 27150185

[ref14] He H, Liu Q, Li N, Guo L, Gao F, Bai L, Gao F and Lyu J (2020) Trends in the incidence and DALYs of schizophrenia at the global, regional and national levels: results from the Global Burden of Disease Study 2017. Epidemiology and Psychiatric Sciences 29, e91. 31928566 10.1017/S2045796019000891 PMC7214712

[ref15] Hilbert A, Pike KM, Goldschmidt AB, Wilfley DE, Fairburn CG, Dohm FA, Walsh BT and Striegel Weissman R (2014) Risk factors across the eating disorders. Psychiatry Research 220, 500–506. 25103674 10.1016/j.psychres.2014.05.054 PMC4785871

[ref16] Hirschberg AL (2012) Sex hormones, appetite and eating behaviour in women. Maturitas 71, 248–256. 22281161 10.1016/j.maturitas.2011.12.016

[ref17] Kolar DR, Rodriguez DL, Chams MM and Hoek HW (2016) Epidemiology of eating disorders in Latin America: a systematic review and meta-analysis. Current Opinion in Psychiatry 29, 363–371. 27584709 10.1097/YCO.0000000000000279

[ref18] Liu Z, Jiang Y, Yuan H, Fang Q, Cai N, Suo C, Jin L, Zhang T and Chen X (2019) The trends in incidence of primary liver cancer caused by specific etiologies: Results from the Global Burden of Disease Study 2016 and implications for liver cancer prevention. Journal of Hepatology 70, 674–683. 30543829 10.1016/j.jhep.2018.12.001

[ref19] Lozano R, Naghavi M, Foreman K, Lim S, Shibuya K, Aboyans V, Abraham J, Adair T, Aggarwal R, Ahn SY, Alvarado M, Anderson HR, Anderson LM, Andrews KG, Atkinson C, Baddour LM, Barker-Collo S, Bartels DH, Bell ML, Benjamin EJ, Bennett D, Bhalla K, Bikbov B, Bin Abdulhak A, Birbeck G, Blyth F, Bolliger I, Boufous S, Bucello C, Burch M, Burney P, Carapetis J, Chen H, Chou D, Chugh SS, Coffeng LE, Colan SD, Colquhoun S, Colson KE, Condon J, Connor MD, Cooper LT, Corriere M, Cortinovis M, de Vaccaro KC, Couser W, Cowie BC, Criqui MH, Cross M, Dabhadkar KC, Dahodwala N, De Leo D, Degenhardt L, Delossantos A, Denenberg J, Des Jarlais DC, Dharmaratne SD, Dorsey ER, Driscoll T, Duber H, Ebel B, Erwin PJ, Espindola P, Ezzati M, Feigin V, Flaxman AD, Forouzanfar MH, Fowkes FG, Franklin R, Fransen M, Freeman MK, Gabriel SE, Gakidou E, Gaspari F, Gillum RF, Gonzalez-Medina D, Halasa YA, Haring D, Harrison JE, Havmoeller R, Hay RJ, Hoen B, Hotez PJ, Hoy D, Jacobsen KH, James SL, Jasrasaria R, Jayaraman S, Johns N, Karthikeyan G, Kassebaum N, Keren A, Khoo JP, Knowlton LM, Kobusingye O, Koranteng A, Krishnamurthi R, Lipnick M, Lipshultz SE, Ohno SL, Mabweijano J, MacIntyre MF, Mallinger L, March L, Marks GB, Marks R, Matsumori A, Matzopoulos R, Mayosi BM, McAnulty JH, McDermott MM, McGrath J, Mensah GA, Merriman TR, Michaud C, Miller M, Miller TR, Mock C, Mocumbi AO, Mokdad AA, Moran A, Mulholland K, Nair MN, Naldi L, Narayan KM, Nasseri K, Norman P, O'Donnell M, Omer SB, Ortblad K, Osborne R, Ozgediz D, Pahari B, Pandian JD, Rivero AP, Padilla RP, Perez-Ruiz F, Perico N, Phillips D, Pierce K, Pope CA 3rd, Porrini E, Pourmalek F, Raju M, Ranganathan D, Rehm JT, Rein DB, Remuzzi G, Rivara FP, Roberts T, De León FR, Rosenfeld LC, Rushton L, Sacco RL, Salomon JA, Sampson U, Sanman E, Schwebel DC, Segui-Gomez M, Shepard DS, Singh D, Singleton J, Sliwa K, Smith E, Steer A, Taylor JA, Thomas B, Tleyjeh IM, Towbin JA, Truelsen T, Undurraga EA, Venketasubramanian N, Vijayakumar L, Vos T, Wagner GR, Wang M, Wang W, Watt K, Weinstock MA, Weintraub R, Wilkinson JD, Woolf AD, Wulf S, Yeh PH, Yip P, Zabetian A, Zheng ZJ, Lopez AD, Murray CJ, AlMazroa MA and Memish ZA (2012) Global and regional mortality from 235 causes of death for 20 age groups in 1990 and 2010: a systematic analysis for the Global Burden of Disease Study 2010. Lancet (London, England) 380, 2095–2128. 10.1016/S0140-6736(12)61728-0 PMC10790329 23245604

[ref20] Lozupone CA, Stombaugh JI, Gordon JI, Jansson JK and Knight R (2012) Diversity, stability and resilience of the human gut microbiota. Nature 489, 220–230. 22972295 10.1038/nature11550 PMC3577372

[ref21] Mitchison D and Mond J (2015) Epidemiology of eating disorders, eating disordered behaviour, and body image disturbance in males: a narrative review. Journal of Eating Disorders 3, 20. 27408719 10.1186/s40337-015-0058-y PMC4940910

[ref22] Mitchison D, Hay P, Slewa-Younan S and Mond J (2014) The changing demographic profile of eating disorder behaviors in the community. BMC Public Health 14, 943. 25213544 10.1186/1471-2458-14-943 PMC4246495

[ref23] Morris PF and Szabo CP (2013) Meanings of thinness and dysfunctional eating in black South African females: a qualitative study. African Journal of Psychiatry (South Africa) 16, 338–342. 10.4314/ajpsy.v16i5.45 24051666

[ref24] Murray CJ, Vos T, Lozano R, Naghavi M, Flaxman AD, Michaud C, Ezzati M, Shibuya K, Salomon JA, Abdalla S, Aboyans V, Abraham J, Ackerman I, Aggarwal R, Ahn SY, Ali MK, Alvarado M, Anderson HR, Anderson LM, Andrews KG, Atkinson C, Baddour LM, Bahalim AN, Barker-Collo S, Barrero LH, Bartels DH, Basáñez MG, Baxter A, Bell ML, Benjamin EJ, Bennett D, Bernabé E, Bhalla K, Bhandari B, Bikbov B, Bin Abdulhak A, Birbeck G, Black JA, Blencowe H, Blore JD, Blyth F, Bolliger I, Bonaventure A, Boufous S, Bourne R, Boussinesq M, Braithwaite T, Brayne C, Bridgett L, Brooker S, Brooks P, Brugha TS, Bryan-Hancock C, Bucello C, Buchbinder R, Buckle G, Budke CM, Burch M, Burney P, Burstein R, Calabria B, Campbell B, Canter CE, Carabin H, Carapetis J, Carmona L, Cella C, Charlson F, Chen H, Cheng AT, Chou D, Chugh SS, Coffeng LE, Colan SD, Colquhoun S, Colson KE, Condon J, Connor MD, Cooper LT, Corriere M, Cortinovis M, de Vaccaro KC, Couser W, Cowie BC, Criqui MH, Cross M, Dabhadkar KC, Dahiya M, Dahodwala N, Damsere-Derry J, Danaei G, Davis A, De Leo D, Degenhardt L, Dellavalle R, Delossantos A, Denenberg J, Derrett S, Des Jarlais DC, Dharmaratne SD, Dherani M, Diaz-Torne C, Dolk H, Dorsey ER, Driscoll T, Duber H, Ebel B, Edmond K, Elbaz A, Ali SE, Erskine H, Erwin PJ, Espindola P, Ewoigbokhan SE, Farzadfar F, Feigin V, Felson DT, Ferrari A, Ferri CP, Fèvre EM, Finucane MM, Flaxman S, Flood L, Foreman K, Forouzanfar MH, Fowkes FG, Fransen M, Freeman MK, Gabbe BJ, Gabriel SE, Gakidou E, Ganatra HA, Garcia B, Gaspari F, Gillum RF, Gmel G, Gonzalez-Medina D, Gosselin R, Grainger R, Grant B, Groeger J, Guillemin F, Gunnell D, Gupta R, Haagsma J, Hagan H, Halasa YA, Hall W, Haring D, Haro JM, Harrison JE, Havmoeller R, Hay RJ, Higashi H, Hill C, Hoen B, Hoffman H, Hotez PJ, Hoy D, Huang JJ, Ibeanusi SE, Jacobsen KH, James SL, Jarvis D, Jasrasaria R, Jayaraman S, Johns N, Jonas JB, Karthikeyan G, Kassebaum N, Kawakami N, Keren A, Khoo JP, King CH, Knowlton LM, Kobusingye O, Koranteng A, Krishnamurthi R, Laden F, Lalloo R, Laslett LL, Lathlean T, Leasher JL, Lee YY, Leigh J, Levinson D, Lim SS, Limb E, Lin JK, Lipnick M, Lipshultz SE, Liu W, Loane M, Ohno SL, Lyons R, Mabweijano J, MacIntyre MF, Malekzadeh R, Mallinger L, Manivannan S, Marcenes W, March L, Margolis DJ, Marks GB, Marks R, Matsumori A, Matzopoulos R, Mayosi BM, McAnulty JH, McDermott MM, McGill N, McGrath J, Medina-Mora ME, Meltzer M, Mensah GA, Merriman TR, Meyer AC, Miglioli V, Miller M, Miller TR, Mitchell PB, Mock C, Mocumbi AO, Moffitt TE, Mokdad AA, Monasta L, Montico M, Moradi-Lakeh M, Moran A, Morawska L, Mori R, Murdoch ME, Mwaniki MK, Naidoo K, Nair MN, Naldi L, Narayan KM, Nelson PK, Nelson RG, Nevitt MC, Newton CR, Nolte S, Norman P, Norman R, O'Donnell M, O'Hanlon S, Olives C, Omer SB, Ortblad K, Osborne R, Ozgediz D, Page A, Pahari B, Pandian JD, Rivero AP, Patten SB, Pearce N, Padilla RP, Perez-Ruiz F, Perico N, Pesudovs K, Phillips D, Phillips MR, Pierce K, Pion S, Polanczyk GV, Polinder S, Pope CA 3rd, Popova S, Porrini E, Pourmalek F, Prince M, Pullan RL, Ramaiah KD, Ranganathan D, Razavi H, Regan M, Rehm JT, Rein DB, Remuzzi G, Richardson K, Rivara FP, Roberts T, Robinson C, De Leòn FR, Ronfani L, Room R, Rosenfeld LC, Rushton L, Sacco RL, Saha S, Sampson U, Sanchez-Riera L, Sanman E, Schwebel DC, Scott JG, Segui-Gomez M, Shahraz S, Shepard DS, Shin H, Shivakoti R, Singh D, Singh GM, Singh JA, Singleton J, Sleet DA, Sliwa K, Smith E, Smith JL, Stapelberg NJ, Steer A, Steiner T, Stolk WA, Stovner LJ, Sudfeld C, Syed S, Tamburlini G, Tavakkoli M, Taylor HR, Taylor JA, Taylor WJ, Thomas B, Thomson WM, Thurston GD, Tleyjeh IM, Tonelli M, Towbin JA, Truelsen T, Tsilimbaris MK, Ubeda C, Undurraga EA, van der Werf MJ, van Os J, Vavilala MS, Venketasubramanian N, Wang M, Wang W, Watt K, Weatherall DJ, Weinstock MA, Weintraub R, Weisskopf MG, Weissman MM, White RA, Whiteford H, Wiebe N, Wiersma ST, Wilkinson JD, Williams HC, Williams SR, Witt E, Wolfe F, Woolf AD, Wulf S, Yeh PH, Zaidi AK, Zheng ZJ, Zonies D, Lopez AD, AlMazroa MA and Memish ZA (2012) Disability-adjusted life years (DALYs) for 291 diseases and injuries in 21 regions, 1990–2010: a systematic analysis for the Global Burden of Disease Study 2010. Lancet (London, England) 380, 2197–2223. 10.1016/S0140-6736(12)61689-4 23245608

[ref25] Murray SB, Nagata JM, Griffiths S, Calzo JP, Brown TA, Mitchison D and Mond JM (2017) The enigma of male eating disorders: a critical review and synthesis. Clinical Psychology Review 57, 1–11. 28800416 10.1016/j.cpr.2017.08.001

[ref26] Pike KM, Hoek HW and Dunne PE (2014) Cultural trends and eating disorders. Current Opinion in Psychiatry 27, 436–442. 25211499 10.1097/YCO.0000000000000100

[ref27] Qian J, Hu Q, Wan Y, Li T, Wu M, Ren Z and Yu D (2013) Prevalence of eating disorders in the general population: a systematic review. Shanghai Archives of Psychiatry 25, 212–223. 24991159 10.3969/j.issn.1002-0829.2013.04.003 PMC4054558

[ref28] Robinson KJ, Mountford VA and Sperlinger DJ (2013) Being men with eating disorders: perspectives of male eating disorder service-users. Journal of Health Psychology 18, 176–186. 22453166 10.1177/1359105312440298

[ref29] Schaumberg K, Welch E, Breithaupt L, Hübel C, Baker JH, Munn-Chernoff MA, Yilmaz Z, Ehrlich S, Mustelin L, Ghaderi A, Hardaway AJ, Bulik-Sullivan EC, Hedman AM, Jangmo A, Nilsson IAK, Wiklund C, Yao S, Seidel M and Bulik CM (2017) The science behind the academy for eating disorders’ nine truths about eating disorders. European Eating Disorders Review 25, 432–450. 28967161 10.1002/erv.2553 PMC5711426

[ref30] Schooler D and Daniels EA (2014) “I am not a skinny toothpick and proud of it”: Latina adolescents’ ethnic identity and responses to mainstream media images. Body Image 11, 11–18. 24125762 10.1016/j.bodyim.2013.09.001

[ref31] Smink FR, van Hoeken D, Oldehinkel AJ and Hoek HW (2014) Prevalence and severity of DSM-5 eating disorders in a community cohort of adolescents. International Journal of Eating Disorders 47, 610–619. 10.1002/eat.22316 24903034

[ref32] Steinhausen HC, Jakobsen H, Helenius D, Munk-Jørgensen P and Strober M (2015) A nation-wide study of the family aggregation and risk factors in anorexia nervosa over three generations. International Journal of Eating Disorders 48, 1–8. 10.1002/eat.22293 24777686

[ref33] Thomas JJ, Lee S and Becker AE (2016) Updates in the epidemiology of eating disorders in Asia and the Pacific. Current Opinion in Psychiatry 29, 354–362. 27648781 10.1097/YCO.0000000000000288

[ref34] Van Hoeken D, Burns JK and Hoek HW (2016) Epidemiology of eating disorders in Africa. Current Opinion in Psychiatry 29, 372–377. 27532943 10.1097/YCO.0000000000000274

[ref35] Vo M, Accurso EC, Goldschmidt AB and Le Grange D (2017) The impact of DSM-5 on eating disorder diagnoses. International Journal of Eating Disorders 50, 578–581. 10.1002/eat.22628 PMC5867898 27862127

[ref36] Wang Q and Tapia Granados JA (2019) Economic growth and mental health in 21st century China. Social Science & Medicine 220, 387–395. 30529490 10.1016/j.socscimed.2018.11.031

[ref37] Warren CS, Gleaves DH and Rakhkovskaya LM (2013) Score reliability and factor similarity of the Sociocultural Attitudes Towards Appearance Questionnaire-3 (SATAQ-3) among four ethnic groups. Journal of Eating Disorders 1, 14. 24999395 10.1186/2050-2974-1-14 PMC4081787

[ref38] Westmoreland P, Krantz MJ and Mehler PS (2016) Medical complications of anorexia nervosa and bulimia. American Journal of Medicine 129, 30–37. 10.1016/j.amjmed.2015.06.031 26169883

[ref39] Wu J, Lin Z, Liu Z, He H, Bai L and Lyu J (2020) Secular trends in the incidence of eating disorders in China from 1990 to 2017: a joinpoint and age-period-cohort analysis. Psychological Medicine. Available at https://www.cambridge.org/core/journals/psychological-medicine/article/secular-trends-in-the-incidence-of-eating-disorders-in-china-from-1990-to-2017-a-joinpoint-and-ageperiodcohort-analysis/B32EA1CEF6F1BCDB4E8337A2C495F5EA. [Epub ahead of print]. 10.1017/S0033291720002706 32744194

[ref40] Yang J, Li Y, Liu Q, Li L, Feng A, Wang T, Zheng S, Xu A and Lyu J (2020) Brief introduction of medical database and data mining technology in big data era. Journal of Evidence-Based Medicine 13, 57–69. 32086994 10.1111/jebm.12373 PMC7065247

[ref41] Zheng Y, Kang Q, Huang J, Jiang W, Liu Q, Chen H, Fan Q, Wang Z, Xiao Z and Chen J (2019) The classification of eating disorders in China: a categorical model or a dimensional model. The International Journal of Eating Disorders 52, 712–720. 30883838 10.1002/eat.23069 PMC6618033

[ref42] Zhou L, Deng Y, Li N, Zheng Y, Tian T, Zhai Z, Yang S, Hao Q, Wu Y, Song D, Zhang D, Lyu J and Dai Z (2019) Global, regional, and national burden of Hodgkin lymphoma from 1990 to 2017: estimates from the 2017 Global Burden of Disease study. Journal of Hematology & Oncology 12, 107. 31640759 10.1186/s13045-019-0799-1 PMC6805485

