# Supplementary material for: Trends in the prevalence and disability-adjusted life years of eating disorders from 1990 to 2017: results from the Global Burden of Disease Study 2017
Source: Epidemiol Psychiatr Sci. 2020 Dec 7;29:e191. doi: 10.1017/S2045796020001055 (PMC7737181; doi:10.1017/S2045796020001055)
Supplement: Supplementary file 1 [file S2045796020001055sup001.zip › Supplementary_Table_5.docx]

**Supplementary Table 5. Age-standardized rates of prevalence and disability-adjusted life-years of bulimia nervosa in 2017 and their temporal trend from 1990 to 2017 at national level.**

|  | **Prevalence (95% UI)** | | | **DALYs (95% UI)** | | |
| --- | --- | --- | --- | --- | --- | --- |
|  | **ASR in 1990**  **(per 100 000 population)** | **ASR in 2017**  **(per 100 000 population)** | **EAPC (%)** | **ASR in 2017**  **(per 100 000 population)** | **ASR in 1990**  **(per 100 000 population)** | **EAPC (%)** |
| Afghanistan | 83.36 (61.48 - 109.37) | 86.54 (64.70 - 111.44) | 0.37 (0.07-0.67) | 16.87 (10.26 - 25.46) | 17.82 (10.84 - 26.93) | 0.44 (0.14-0.74) |
| Albania | 101.74 (75.53 - 132.98) | 134.04 (100.92 - 172.08) | 1.37 (1.21-1.52) | 21.48 (13.10 - 32.67) | 28.44 (17.52 - 43.67) | 1.38 (1.23-1.53) |
| Algeria | 150.96 (113.92 - 194.66) | 167.68 (128.03 - 215.32) | 0.57 (0.47-0.67) | 31.60 (19.53 - 47.81) | 35.30 (21.59 - 53.06) | 0.59 (0.49-0.69) |
| American Samoa | 136.84 (103.38 - 177.47) | 128.96 (96.46 - 166.86) | -0.16 (-0.19 to -0.13) | 28.88 (17.52 - 43.49) | 27.24 (16.59 - 41.04) | -0.16 (-0.19 to -0.12) |
| Andorra | 405.67 (311.20 - 526.77) | 446.73 (340.83 - 570.72) | 0.53 (0.45-0.61) | 85.57 (53.66 - 129.76) | 94.29 (58.42 - 142.18) | 0.53 (0.45-0.61) |
| Angola | 109.86 (82.51 - 141.88) | 136.33 (102.76 - 176.61) | 1.02 (0.78-1.27) | 22.74 (14.14 - 33.60) | 28.50 (17.50 - 42.51) | 1.05 (0.80-1.30) |
| Antigua and Barbuda | 245.86 (185.70 - 315.79) | 285.61 (217.98 - 371.68) | 0.56 (0.51-0.61) | 51.82 (31.88 - 78.37) | 60.19 (37.28 - 90.10) | 0.57 (0.51-0.62) |
| Argentina | 239.30 (184.69 - 307.21) | 293.77 (222.24 - 379.92) | 0.69 (0.62-0.76) | 50.58 (31.43 - 75.59) | 62.11 (38.53 - 94.09) | 0.68 (0.61-0.75) |
| Armenia | 109.13 (80.85 - 140.49) | 128.04 (94.91 - 165.59) | 1.07 (0.73-1.42) | 23.18 (14.11 - 34.85) | 27.21 (17.03 - 41.42) | 1.07 (0.73-1.41) |
| Australia | 542.89 (416.81 - 695.01) | 734.05 (585.83 - 912.64) | 1.38 (1.25-1.51) | 114.36 (70.84 - 169.97) | 154.27 (100.00 - 224.58) | 1.38 (1.25-1.51) |
| Austria | 387.96 (297.27 - 493.55) | 486.51 (373.92 - 619.26) | 0.90 (0.83-0.98) | 81.87 (50.70 - 121.87) | 102.92 (64.36 - 154.81) | 0.92 (0.84-1.00) |
| Azerbaijan | 129.77 (97.44 - 166.09) | 157.08 (118.90 - 202.61) | 1.12 (0.58-1.66) | 27.54 (17.07 – 41.00) | 33.43 (20.35 - 50.16) | 1.12 (0.58-1.66) |
| Bahrain | 193.14 (147.39 - 246.01) | 219.41 (165.33 - 279.63) | 0.44 (0.40-0.47) | 40.48 (25.21 - 60.21) | 46.14 (28.21 - 69.94) | 0.45 (0.42-0.48) |
| Bangladesh | 79.64 (59.71 - 101.95) | 107.90 (80.55 - 139.55) | 1.11 (1.00-1.22) | 16.68 (10.04 - 24.77) | 22.76 (13.95 - 34.37) | 1.14 (1.03-1.25) |
| Barbados | 247.55 (190.66 - 318.02) | 257.97 (196.48 - 333.81) | 0.22 (0.16-0.27) | 52.57 (33.03 - 78.04) | 54.75 (34.57 - 81.95) | 0.22 (0.16-0.27) |
| Belarus | 131.97 (99.69 - 169.84) | 158.60 (119.72 - 204.93) | 0.93 (0.67-1.20) | 27.84 (17.10 - 42.31) | 33.35 (20.60 - 49.91) | 0.93 (0.67-1.20) |
| Belgium | 354.16 (270.56 - 455.39) | 422.05 (324.59 - 536.74) | 0.65 (0.62-0.68) | 74.60 (46.32 - 112.56) | 88.88 (56.01 - 132.13) | 0.65 (0.62-0.68) |
| Belize | 173.94 (133.06 - 226.68) | 211.06 (161.43 - 273.75) | 0.66 (0.59-0.72) | 36.78 (22.92 - 55.33) | 44.62 (27.90 - 67.25) | 0.66 (0.60-0.72) |
| Benin | 86.60 (64.75 - 112.20) | 94.27 (70.69 - 121.14) | 0.39 (0.33-0.44) | 17.91 (10.81 - 27.47) | 19.79 (12.19 - 29.85) | 0.44 (0.38-0.49) |
| Bermuda | 342.42 (259.05 - 446.54) | 393.94 (300.11 - 516.98) | 0.70 (0.63-0.76) | 72.77 (45.35 - 109.34) | 83.64 (52.02 - 127.01) | 0.70 (0.63-0.77) |
| Bhutan | 91.57 (68.57 - 118.71) | 134.15 (100.12 - 173.74) | 1.48 (1.46-1.51) | 19.15 (11.84 - 28.91) | 28.32 (17.46 - 42.83) | 1.52 (1.49-1.55) |
| Bolivia | 212.06 (161.18 - 276.68) | 262.68 (199.50 - 342.74) | 0.77 (0.71-0.84) | 44.63 (27.45 - 67.39) | 55.49 (34.64 - 83.26) | 0.79 (0.73-0.85) |
| Bosnia and Herzegovina | 77.75 (57.82 - 99.84) | 132.52 (98.45 - 170.39) | 2.46 (2.18-2.74) | 16.41 (9.95 - 24.47) | 28.02 (17.72 - 41.81) | 2.47 (2.18-2.75) |
| Botswana | 135.18 (100.26 - 173.77) | 175.70 (132.34 - 227.78) | 0.94 (0.92-0.96) | 28.24 (17.31 - 42.40) | 36.65 (22.73 - 55.66) | 0.94 (0.93-0.95) |
| Brazil | 185.57 (141.13 - 240.66) | 218.89 (167.67 - 284.14) | 0.63 (0.59-0.68) | 39.01 (24.51 - 58.79) | 46.15 (29.21 - 68.71) | 0.64 (0.60-0.69) |
| Brunei | 387.26 (297.19 - 499.11) | 368.28 (280.82 - 480.23) | -0.07 (-0.11 to -0.03) | 82.30 (51.57 - 122.88) | 78.52 (49.07 - 117.96) | -0.06 (-0.10 to -0.03) |
| Bulgaria | 135.63 (102.54 - 174.85) | 155.29 (117.90 - 201.35) | 0.66 (0.46-0.85) | 28.76 (17.57 - 43.21) | 33.02 (20.56 - 49.33) | 0.67 (0.47-0.86) |
| Burkina Faso | 74.73 (55.44 - 96.59) | 88.77 (65.60 - 115.37) | 0.69 (0.64-0.74) | 15.4 (9.26 - 23.16) | 18.70 (11.49 - 28.90) | 0.76 (0.71-0.82) |
| Burundi | 75.15 (55.55 - 97.79) | 70.36 (52.22 - 91.29) | -0.33 (-0.44 to -0.21) | 15.79 (9.73 - 23.69) | 14.80 (8.98 - 22.32) | -0.33 (-0.46 to -0.20) |
| Cambodia | 56.74 (42.18 - 73.29) | 79.84 (59.49 - 102.84) | 1.37 (1.25-1.48) | 11.88 (7.10 - 18.23) | 16.88 (10.47 - 25.32) | 1.42 (1.30-1.54) |
| Cameroon | 106.62 (79.18 - 137.74) | 104.58 (77.33 - 135.39) | 0.03 (-0.08 to 0.14) | 22.08 (13.52 - 32.80) | 21.95 (13.61 - 32.90) | 0.08 (-0.03-0.19) |
| Canada | 313.64 (242.34 - 404.96) | 335.9 (255.79 - 433.97) | 0.32 (0.28-0.35) | 66.12 (41.76 - 99.52) | 70.83 (44.52 - 105.93) | 0.32 (0.28-0.35) |
| Cape Verde | 95.00 (71.78 - 123.25) | 128.88 (97.72 - 167.05) | 1.31 (1.25-1.37) | 20.04 (12.25 - 30.47) | 27.25 (17.20 - 41.14) | 1.34 (1.28-1.41) |
| Central African Republic | 77.15 (57.45 - 100.21) | 69.66 (51.53 - 90.39) | -0.27 (-0.31 to -0.23) | 15.94 (9.66 - 23.88) | 14.61 (8.96 - 22.20) | -0.22 (-0.26 to -0.18) |
| Chad | 81.06 (60.07 - 103.41) | 95.68 (70.60 - 124.39) | 0.69 (0.58-0.80) | 16.80 (10.36 - 25.09) | 19.95 (12.19 - 29.88) | 0.72 (0.60-0.84) |
| Chile | 223.55 (169.84 - 287.22) | 301.10 (231.02 - 386.66) | 1.08 (1.06-1.10) | 47.10 (29.30 - 70.07) | 63.42 (39.30 - 95.84) | 1.08 (1.06-1.10) |
| China | 60.68 (44.59 - 79.50) | 111.28 (82.28 - 143.60) | 2.35 (2.23-2.46) | 12.90 (7.97 - 19.19) | 23.78 (14.80 - 35.82) | 2.36 (2.25-2.47) |
| Colombia | 168.35 (127.17 - 219.20) | 203.93 (155.8 - 263.27) | 0.64 (0.54-0.73) | 35.75 (22.09 - 54.41) | 43.43 (26.81 - 64.79) | 0.65 (0.56-0.75) |
| Comoros | 91.21 (67.29 - 118.10) | 85.52 (62.91 - 111.21) | -0.21 (-0.25 to -0.16) | 19.13 (11.77 - 28.66) | 18.11 (11.02 - 27.52) | -0.18 (-0.22 to -0.13) |
| Congo | 117.41 (87.28 - 152.15) | 126.95 (93.34 - 164.28) | 0.36 (0.23-0.48) | 24.38 (14.76 - 36.49) | 26.62 (16.32 - 39.73) | 0.39 (0.26-0.52) |
| Costa Rica | 187.26 (141.36 - 243.86) | 228.6 (175.58 - 296.75) | 0.73 (0.70-0.77) | 39.77 (24.65 - 61.04) | 48.55 (30.45 - 73.17) | 0.74 (0.70-0.77) |
| Cote d'Ivoire | 105.55 (80.21 - 136.28) | 106.94 (78.92 - 138.74) | -0.05 (-0.15 to 0.04) | 21.96 (13.46 - 32.67) | 22.44 (13.70 - 33.84) | -0.02 (-0.12 to 0.08) |
| Croatia | 147.93 (109.91 - 190.96) | 165.84 (124.88 - 210.66) | 0.74 (0.61-0.86) | 31.34 (19.55 - 47.33) | 35.25 (21.78 - 52.48) | 0.76 (0.63-0.89) |
| Cuba | 190.90 (144.70 - 246.06) | 211.67 (159.82 - 273.69) | 0.65 (0.42-0.87) | 40.45 (24.54 - 60.84) | 44.93 (27.54 - 67.86) | 0.66 (0.44-0.88) |
| Cyprus | 295.19 (225.71 - 377.06) | 356.57 (275.00 - 457.14) | 0.82 (0.73-0.90) | 62.09 (38.55 - 93.22) | 75.12 (46.10 - 112.24) | 0.82 (0.74-0.91) |
| Czech Republic | 160.42 (120.11 - 204.33) | 183.81 (136.76 - 236.63) | 0.65 (0.58-0.73) | 33.91 (21.03 - 50.43) | 38.87 (23.58 - 57.73) | 0.66 (0.58-0.73) |
| Democratic Republic of the Congo | 85.64 (64.08 - 110.25) | 72.74 (53.78 - 95.25) | -0.82 (-1.16 to -0.48) | 17.70 (10.83 - 26.42) | 15.22 (9.31 - 22.73) | -0.77 (-1.12 to -0.42) |
| Denmark | 335.44 (256.69 - 427.92) | 382.68 (294.04 - 490.03) | 0.56 (0.51-0.61) | 70.77 (44.75 - 106.41) | 80.51 (51.21 – 121.00) | 0.55 (0.50-0.60) |
| Djibouti | 107.56 (79.33 - 139.89) | 105.29 (78.40 - 136.25) | -0.05 (-0.23 to 0.12) | 22.73 (14.23 - 34.24) | 22.35 (13.76 - 33.57) | -0.04 (-0.22 to 0.14) |
| Dominica | 190.87 (143.83 - 249.38) | 225.34 (170.62 - 291.96) | 0.64 (0.62-0.66) | 40.45 (24.68 - 60.68) | 47.70 (28.92 - 71.35) | 0.64 (0.62-0.66) |
| Dominican Republic | 190.70 (144.94 - 245.90) | 247.23 (186.85 - 320.60) | 1.01 (0.96-1.05) | 40.47 (25.06 - 60.95) | 52.56 (32.32 - 80.04) | 1.01 (0.97-1.05) |
| Ecuador | 261.77 (196.66 - 343.29) | 309.19 (234.54 - 401.29) | 0.63 (0.54-0.72) | 55.38 (34.27 - 84.37) | 65.62 (40.65 - 100.36) | 0.64 (0.56-0.73) |
| Egypt | 119.10 (89.42 - 153.78) | 154.77 (116.10 - 200.22) | 1.04 (1.02-1.07) | 24.80 (15.19 - 37.11) | 32.53 (19.79 - 49.56) | 1.06 (1.03-1.08) |
| El Salvador | 150.73 (113.93 - 195.17) | 194.53 (148.19 - 255.43) | 0.98 (0.96-0.99) | 31.89 (19.85 - 48.15) | 41.37 (25.25 - 62.83) | 0.99 (0.98-1.01) |
| Equatorial Guinea | 75.04 (55.71 - 96.85) | 218.78 (167.04 - 282.05) | 5.27 (4.57-5.97) | 15.52 (9.40 - 23.30) | 45.84 (28.41 - 68.12) | 5.32 (4.62-6.03) |
| Eritrea | 68.40 (50.29 - 88.42) | 79.78 (58.92 - 103.44) | 0.40 (0.19-0.61) | 14.02 (8.50 - 21.54) | 16.76 (10.16 - 25.54) | 0.49 (0.27-0.70) |
| Estonia | 151.65 (115.54 - 196.02) | 180.17 (136.62 - 230.42) | 0.90 (0.78-1.03) | 32.36 (20.3 - 48.47) | 38.26 (23.88 - 57.13) | 0.87 (0.75-0.99) |
| Ethiopia | 65.56 (48.51 - 85.43) | 85.52 (62.98 - 109.91) | 1.06 (0.76-1.36) | 13.65 (8.57 - 20.57) | 18.05 (11.14 - 27.06) | 1.12 (0.83-1.42) |
| Federated States of Micronesia | 74.03 (54.73 - 95.87) | 80.28 (59.11 - 103.73) | 0.28 (0.25-0.32) | 15.6 (9.48 - 23.74) | 16.99 (10.36 - 25.67) | 0.30 (0.26-0.33) |
| Fiji | 94.01 (70.52 - 121.09) | 107.91 (80.33 - 137.76) | 0.47 (0.45-0.48) | 19.80 (12.16 - 30.09) | 22.77 (13.90 - 34.38) | 0.47 (0.46-0.49) |
| Finland | 341.53 (259.95 - 435.07) | 388.09 (298.67 - 499.93) | 0.57 (0.48-0.66) | 72.01 (44.77 - 106.70) | 81.97 (51.69 - 122.86) | 0.58 (0.49-0.67) |
| France | 340.87 (262.03 - 434.73) | 392.13 (299.85 - 505.78) | 0.53 (0.50-0.56) | 71.90 (44.43 - 107.80) | 82.90 (51.29 - 126.18) | 0.54 (0.51-0.56) |
| Gabon | 171.02 (130.43 - 219.64) | 176.64 (133.93 - 226.35) | 0.10 (0.08-0.12) | 35.64 (21.73 - 53.84) | 37.00 (22.73 - 54.97) | 0.11 (0.09-0.13) |
| Georgia | 131.59 (97.36 - 168.88) | 131.10 (97.05 - 168.85) | 0.35 (-0.13 to 0.82) | 28.12 (17.55 - 42.17) | 27.96 (17.15 - 42.55) | 0.34 (-0.13 to 0.81) |
| Germany | 281.04 (220.96 - 357.76) | 319.87 (247.45 - 409.92) | 0.49 (0.45-0.53) | 59.52 (37.78 - 90.35) | 67.82 (42.62 - 102.50) | 0.49 (0.45-0.53) |
| Ghana | 91.86 (68.62 - 118.85) | 114.76 (84.99 - 148.84) | 0.76 (0.68-0.84) | 19.25 (11.95 - 28.96) | 24.21 (15.14 - 36.21) | 0.78 (0.69-0.86) |
| Greece | 356.59 (275.63 - 456.20) | 401.16 (309.98 - 512.34) | 0.61 (0.51-0.72) | 75.07 (46.96 - 113.15) | 84.52 (53.35 - 127.23) | 0.62 (0.51-0.73) |
| Greenland | 344.98 (267.74 - 445.29) | 379.74 (292.12 - 483.67) | 0.47 (0.40-0.54) | 72.19 (46.20 - 106.94) | 79.83 (50.60 - 119.82) | 0.49 (0.42-0.55) |
| Grenada | 185.38 (139.33 - 240.28) | 234.75 (177.49 - 304.93) | 0.97 (0.92-1.03) | 39.22 (24.08 - 58.89) | 49.73 (30.82 - 75.82) | 0.98 (0.93-1.03) |
| Guam | 170.78 (128.27 - 220.41) | 182.93 (138.06 - 234.92) | 0.32 (0.29-0.35) | 36.29 (22.09 - 54.07) | 38.89 (24.45 - 58.63) | 0.32 (0.29-0.35) |
| Guatemala | 164.86 (124.87 - 213.21) | 186.12 (142.40 - 240.75) | 0.46 (0.44-0.47) | 34.66 (21.75 - 51.68) | 39.50 (24.62 - 59.61) | 0.49 (0.47-0.51) |
| Guinea | 87.97 (65.88 - 113.09) | 87.84 (65.81 - 113.81) | -0.09 (-0.16 to -0.02) | 18.31 (11.26 - 27.60) | 18.45 (11.09 - 27.95) | -0.06 (-0.13 to 0.01) |
| Guinea-Bissau | 85.28 (62.54 - 110.85) | 86.65 (64.58 - 112.26) | -0.04 (-0.07 to 0.01) | 17.72 (10.69 - 26.97) | 18.14 (11.10 - 27.23) | -0.02 (-0.06 to 0.02) |
| Guyana | 152.21 (115.93 - 196.44) | 195.46 (147.25 - 252.30) | 0.89 (0.84-0.94) | 31.97 (19.81 - 48.22) | 41.12 (25.75 - 62.09) | 0.91 (0.86-0.96) |
| Haiti | 136.66 (101.96 - 177.72) | 133.50 (100.02 - 172.92) | -0.03 (-0.08 to 0.02) | 28.47 (17.43 - 42.62) | 27.83 (17.12 - 41.39) | -0.02 (-0.06 to 0.03) |
| Honduras | 145.89 (109.48 - 191.41) | 163.93 (121.70 - 214.06) | 0.46 (0.41-0.50) | 30.94 (19.14 - 46.60) | 34.78 (21.58 - 53.02) | 0.46 (0.41-0.51) |
| Hungary | 155.99 (119.93 - 198.86) | 182.35 (137.19 - 237.08) | 0.73 (0.66-0.80) | 32.90 (20.50 - 49.13) | 38.63 (24.22 - 58.22) | 0.74 (0.67-0.81) |
| Iceland | 340.11 (260.91 - 433.87) | 390.77 (297.68 - 500.95) | 0.63 (0.59-0.66) | 71.78 (44.59 - 107.73) | 82.31 (51.46 - 122.73) | 0.63 (0.59-0.66) |
| India | 82.93 (61.43 - 106.63) | 124.93 (93.01 - 159.99) | 1.59 (1.49-1.68) | 17.33 (10.77 - 25.76) | 26.28 (16.32 - 39.20) | 1.62 (1.52-1.71) |
| Indonesia | 79.18 (58.90 - 101.54) | 109.52 (81.29 - 140.95) | 1.03 (0.94-1.12) | 16.76 (10.6 - 25.06) | 23.25 (14.41 - 34.65) | 1.04 (0.96-1.13) |
| Iran | 144.20 (109.13 - 184.04) | 184.84 (139.90 - 237.13) | 1.10 (1.03-1.17) | 30.12 (18.88 - 44.21) | 38.82 (24.34 - 57.28) | 1.12 (1.05-1.19) |
| Iraq | 144.82 (107.87 - 187.20) | 167.74 (126.58 - 214.60) | 0.85 (0.66-1.05) | 30.03 (18.45 - 44.79) | 35.03 (21.48 - 51.87) | 0.89 (0.70-1.09) |
| Ireland | 276.91 (210.91 - 354.56) | 396.17 (304.21 - 515.77) | 1.44 (1.35-1.53) | 58.15 (36.34 - 87.27) | 83.28 (51.29 - 124.95) | 1.44 (1.35-1.53) |
| Israel | 273.87 (210.41 - 353.62) | 339.91 (259.72 - 434.02) | 0.80 (0.76-0.84) | 57.75 (36.04 - 86.66) | 71.63 (44.94 - 107.82) | 0.80 (0.76-0.85) |
| Italy | 425.41 (329.34 - 538.28) | 452.48 (350.87 - 572.23) | 0.16 (0.08-0.25) | 89.97 (56.16 - 133.67) | 95.56 (60.12 - 142.04) | 0.16 (0.07-0.24) |
| Jamaica | 195.23 (148.14 - 253.35) | 212.08 (161.70 - 276.98) | 0.27 (0.24-0.29) | 41.37 (25.38 - 62.64) | 44.86 (28.31 - 68.34) | 0.26 (0.23-0.29) |
| Japan | 273.89 (210.92 - 349.89) | 304.96 (235.25 - 390.88) | 0.30 (0.24-0.36) | 58.24 (36.76 - 86.65) | 64.64 (41.06 - 96.78) | 0.29 (0.23-0.35) |
| Jordan | 131.45 (98.95 - 171.70) | 149.66 (113.87 - 193.60) | 0.70 (0.59-0.82) | 27.58 (16.84 - 41.55) | 31.57 (19.58 - 47.32) | 0.72 (0.60-0.83) |
| Kazakhstan | 146.01 (109.88 - 188.84) | 174.38 (131.54 - 225.79) | 0.92 (0.62-1.21) | 30.87 (19.17 - 46.18) | 36.92 (22.63 - 55.46) | 0.91 (0.62-1.21) |
| Kenya | 96.63 (71.44 - 124.35) | 105.25 (77.86 - 134.89) | 0.26 (0.17-0.34) | 20.39 (12.74 - 30.31) | 22.21 (13.85 - 33.17) | 0.27 (0.18-0.36) |
| Kiribati | 70.26 (51.62 - 91.65) | 68.81 (50.86 - 88.54) | -0.06 (-0.09 to -0.04) | 14.74 (8.94 - 22.19) | 14.49 (8.96 - 21.89) | -0.06 (-0.09 to -0.04) |
| Kuwait | 222.42 (169.74 - 287.63) | 269.05 (204.47 - 345.37) | 0.78 (0.73-0.83) | 46.72 (29.07 - 69.81) | 56.79 (34.25 - 85.49) | 0.79 (0.74-0.83) |
| Kyrgyzstan | 111.81 (84.21 - 145.19) | 98.66 (73.45 - 128.47) | -0.43 (-0.73 to -0.13) | 23.77 (14.73 - 35.57) | 20.96 (12.85 - 31.73) | -0.43 (-0.72 to -0.14) |
| Laos | 63.89 (46.99 - 83.05) | 93.74 (69.84 - 121.71) | 1.41 (1.30-1.52) | 13.47 (8.12 - 20.36) | 19.91 (12.14 - 29.92) | 1.44 (1.33-1.55) |
| Latvia | 150.62 (113.40 - 194.06) | 169.02 (128.34 - 219.98) | 0.80 (0.54-1.05) | 31.80 (19.62 - 47.40) | 35.70 (21.79 - 53.73) | 0.80 (0.54-1.05) |
| Lebanon | 150.09 (112.95 - 195.04) | 173.45 (131.13 - 224.07) | 0.74 (0.67-0.81) | 31.40 (19.77 - 47.49) | 36.41 (22.05 - 54.78) | 0.75 (0.68-0.82) |
| Lesotho | 84.51 (62.95 - 109.93) | 106.75 (78.84 - 137.34) | 0.85 (0.83-0.87) | 17.65 (10.95 - 26.70) | 22.18 (13.59 - 33.05) | 0.82 (0.79-0.86) |
| Liberia | 77.37 (57.76 - 100.35) | 71.98 (53.10 - 93.16) | 0.16 (-0.14 to 0.45) | 16.05 (9.71 - 23.92) | 14.88 (9.11 - 22.33) | 0.16 (-0.15 to 0.47) |
| Libya | 192.73 (146.83 - 247.31) | 155.60 (117.92 - 197.92) | -0.26 (-0.48 to -0.04) | 40.49 (24.71 - 60.52) | 32.64 (20.41 - 48.67) | -0.27 (-0.49 to -0.05) |
| Lithuania | 147.40 (111.20 - 189.23) | 175.90 (134.25 - 226.71) | 0.95 (0.72-1.19) | 31.06 (19.45 - 46.77) | 37.08 (22.78 - 55.66) | 0.96 (0.72-1.20) |
| Luxembourg | 401.7 (305.86 - 513.34) | 506.44 (390.38 - 647.20) | 0.87 (0.79-0.95) | 84.74 (52.33 - 126.11) | 106.71 (66.65 - 161.32) | 0.87 (0.78-0.95) |
| Macedonia | 128.37 (95.81 - 166.20) | 141.37 (105.79 - 183.87) | 0.49 (0.35-0.64) | 27.14 (16.69 - 40.72) | 30.03 (18.54 - 45.09) | 0.51 (0.36-0.65) |
| Madagascar | 85.36 (62.88 - 110.08) | 83.41 (61.93 - 108.09) | -0.09 (-0.16 to -0.02) | 17.85 (10.86 - 27.14) | 17.60 (10.55 - 26.53) | -0.07 (-0.14 to 0.01) |
| Malawi | 74.01 (55.22 - 95.86) | 78.00 (57.71 - 100.84) | 0.29 (0.22-0.35) | 15.47 (9.48 - 23.21) | 16.39 (9.88 - 24.70) | 0.33 (0.25-0.41) |
| Malaysia | 105.47 (78.64 - 136.32) | 142.69 (106.25 - 183.83) | 1.07 (1.03-1.11) | 22.36 (14.03 - 33.45) | 30.36 (18.42 - 45.93) | 1.07 (1.03-1.12) |
| Maldives | 85.34 (63.57 - 110.46) | 115.18 (86.59 - 148.77) | 1.25 (1.16-1.35) | 17.94 (10.84 - 27.16) | 24.52 (15.32 - 36.91) | 1.30 (1.21-1.40) |
| Mali | 77.69 (57.28 - 100.71) | 91.17 (66.97 - 118.82) | 0.64 (0.62-0.67) | 16.10 (9.79 - 24.24) | 19.09 (12.02 - 28.94) | 0.69 (0.67-0.72) |
| Malta | 272.58 (208.07 - 352.94) | 360.28 (276.32 - 458.62) | 1.01 (0.95-1.07) | 57.66 (36.17 - 86.52) | 76.08 (46.87 - 113.69) | 1.00 (0.94-1.06) |
| Marshall Islands | 75.07 (55.51 - 98.01) | 82.56 (61.27 - 105.80) | 0.29 (0.25-0.33) | 15.86 (9.70 - 23.79) | 17.48 (10.88 - 26.42) | 0.30 (0.26-0.34) |
| Mauritania | 101.04 (75.59 - 130.73) | 112.87 (84.67 - 146.29) | 0.44 (0.35-0.53) | 21.16 (13.13 - 31.61) | 23.78 (14.53 - 35.71) | 0.46 (0.37-0.55) |
| Mauritius | 98.24 (73.99 - 127.39) | 133.13 (99.22 - 172.84) | 1.05 (1.01-1.09) | 20.76 (12.61 - 31.77) | 28.13 (17.28 - 42.60) | 1.06 (1.01-1.10) |
| Mexico | 238.84 (183.42 - 307.52) | 253.84 (194.39 - 325.98) | 0.22 (0.21-0.24) | 50.81 (31.95 - 75.95) | 54.03 (34.06 - 81.05) | 0.22 (0.20-0.23) |
| Moldova | 118.03 (88.79 - 151.45) | 106.92 (80.21 - 137.50) | -0.25 (-0.63 to 0.13) | 24.88 (15.39 - 37.17) | 22.66 (14.08 - 34.48) | -0.24 (-0.61 to 0.13) |
| Mongolia | 106.87 (79.62 - 138.31) | 138.97 (103.19 - 178.21) | 1.07 (0.87-1.28) | 22.71 (14.01 - 34.16) | 29.44 (17.97 - 44.46) | 1.06 (0.85-1.26) |
| Montenegro | 135.78 (102.72 - 175.3) | 148.25 (112.47 - 190.54) | 0.62 (0.43-0.80) | 28.76 (17.80 - 43.57) | 31.46 (19.80 - 47.09) | 0.62 (0.44-0.8) |
| Morocco | 112.66 (86.13 - 145.41) | 139.95 (105.02 - 180.79) | 0.79 (0.74-0.83) | 23.47 (14.48 - 35.36) | 29.33 (18.01 - 44.49) | 0.81 (0.76-0.85) |
| Mozambique | 60.08 (44.29 - 78.32) | 80.45 (60.23 - 104.32) | 1.30 (1.20-1.39) | 12.51 (7.46 - 18.89) | 16.78 (10.35 - 25.63) | 1.31 (1.21-1.41) |
| Myanmar | 54.77 (40.32 - 70.95) | 92.00 (68.49 - 118.69) | 2.18 (1.98-2.38) | 11.51 (6.91 - 17.60) | 19.52 (12.05 - 29.52) | 2.21 (2.01-2.41) |
| Namibia | 132.94 (99.46 - 171.73) | 155.77 (116.22 - 202.03) | 0.63 (0.53-0.74) | 27.85 (17.18 - 41.83) | 32.60 (20.26 - 48.99) | 0.64 (0.53-0.75) |
| Nepal | 77.02 (57.51 - 99.39) | 99.84 (74.28 - 130.07) | 0.92 (0.86-0.98) | 16.00 (9.88 - 23.92) | 21.00 (12.95 - 31.95) | 0.98 (0.92-1.03) |
| Netherlands | 217.8 (172.33 - 274.37) | 285.07 (217.37 - 368.29) | 1.14 (1.09-1.19) | 46.04 (29.22 - 67.52) | 60.29 (37.57 - 90.38) | 1.16 (1.10-1.21) |
| New Zealand | 427.91 (323.97 - 554.58) | 458.01 (348.76 - 586.69) | 0.32 (0.29-0.36) | 89.29 (56.36 - 133.62) | 95.69 (60.01 - 144.04) | 0.33 (0.29-0.36) |
| Nicaragua | 152.73 (115.94 - 197.47) | 162.77 (124.21 - 212.04) | 0.33 (0.23-0.42) | 32.15 (19.55 - 47.73) | 34.70 (21.42 - 52.34) | 0.37 (0.28-0.46) |
| Niger | 75.50 (55.32 - 99.13) | 75.23 (55.62 - 97.33) | -0.01 (-0.12 to 0.10) | 15.77 (9.68 - 23.81) | 15.84 (9.61 - 24.05) | 0.03 (-0.09-0.14) |
| Nigeria | 100.93 (75.05 - 130.79) | 128.2 (95.98 - 166.03) | 1.26 (0.99-1.52) | 21.03 (12.66 - 31.35) | 26.79 (16.65 - 40.50) | 1.27 (1.00-1.54) |
| North Korea | 83.48 (61.67 - 107.24) | 64.32 (47.35 - 83.41) | -1.10 (-1.31 to -0.90) | 17.74 (10.98 - 27.11) | 13.68 (8.36 - 20.60) | -1.10 (-1.30 to -0.89) |
| Northern Mariana Islands | 181.06 (138.84 - 234.59) | 141.49 (105.76 - 182.58) | -0.90 (-0.96 to -0.84) | 38.49 (23.67 - 58.29) | 29.99 (18.72 - 44.94) | -0.91 (-0.96 to -0.85) |
| Norway | 381.99 (295.58 - 484.97) | 411.34 (318.11 - 525.91) | 0.29 (0.26-0.32) | 80.98 (51.42 - 120.31) | 86.5 (54.71 - 129.45) | 0.26 (0.23-0.28) |
| Oman | 175.09 (131.13 - 224.61) | 206.04 (155.79 - 262.92) | 0.73 (0.59-0.87) | 36.64 (22.34 - 54.76) | 43.52 (27.19 - 66.12) | 0.75 (0.61-0.90) |
| Pakistan | 99.04 (73.77 - 126.88) | 120.63 (90.13 - 155.11) | 0.76 (0.71-0.81) | 20.81 (12.68 - 31.57) | 25.46 (15.85 - 38.42) | 0.78 (0.73-0.84) |
| Palestine | 97.65 (72.50 - 125.81) | 114.00 (86.01 - 146.67) | 0.45 (0.38-0.53) | 20.33 (12.40 - 29.84) | 23.91 (15.00 - 36.06) | 0.46 (0.38-0.54) |
| Panama | 188.8 (143.38 - 242.59) | 250.73 (190.07 - 327.89) | 1.02 (0.94-1.11) | 40.21 (24.60 - 61.08) | 53.53 (33.48 - 81.04) | 1.03 (0.95-1.12) |
| Papua New Guinea | 68.59 (50.61 - 88.43) | 78.05 (57.48 - 101.67) | 0.30 (0.22-0.38) | 14.40 (8.75 - 21.77) | 16.40 (9.95 - 24.76) | 0.30 (0.23-0.38) |
| Paraguay | 161.60 (121.72 - 208.97) | 188.44 (142.01 - 245.50) | 0.49 (0.41-0.57) | 34.17 (20.95 - 50.85) | 39.95 (24.50 - 60.34) | 0.50 (0.43-0.58) |
| Peru | 253.15 (189.75 - 331.56) | 316.02 (240.26 - 418.27) | 0.90 (0.85-0.95) | 53.52 (32.90 - 80.85) | 67.18 (41.59 - 101.39) | 0.92 (0.87-0.97) |
| Philippines | 81.66 (60.11 - 105.27) | 98.92 (72.80 - 127.29) | 0.65 (0.58-0.73) | 17.23 (10.28 - 26.18) | 21.01 (12.73 - 31.93) | 0.68 (0.60-0.75) |
| Poland | 129.69 (97.52 - 169.53) | 173.34 (132.41 - 221.56) | 1.20 (1.14-1.26) | 27.47 (16.79 - 41.21) | 36.76 (22.73 - 55.59) | 1.21 (1.15-1.27) |
| Portugal | 279.63 (214.15 - 358.31) | 353.56 (270.72 - 453.20) | 0.92 (0.84-1.01) | 58.67 (36.59 - 88.40) | 74.29 (45.89 - 110.18) | 0.93 (0.84-1.02) |
| Puerto Rico | 286.51 (219.98 - 374.02) | 331.38 (250.76 - 428.60) | 0.58 (0.49-0.67) | 60.94 (38.29 - 91.45) | 70.41 (44.44 - 106.12) | 0.58 (0.49-0.67) |
| Qatar | 229.87 (174.25 - 293.88) | 255.04 (193.53 - 328.19) | 0.62 (0.48-0.76) | 48.31 (29.94 - 71.44) | 53.87 (33.27 - 81.68) | 0.63 (0.49-0.78) |
| Romania | 125.98 (94.31 - 162.12) | 153.36 (115.35 - 199.46) | 0.96 (0.77-1.15) | 26.57 (16.19 - 40.22) | 32.41 (20.24 - 48.88) | 0.97 (0.79-1.16) |
| Russian Federation | 167.95 (127.25 - 214.90) | 175.94 (132.68 - 225.85) | 0.33 (0.04-0.61) | 35.44 (22.33 - 52.50) | 37.09 (23.30 - 54.93) | 0.31 (0.02-0.60) |
| Rwanda | 77.17 (57.46 - 100.05) | 89.46 (66.61 - 115.97) | 0.69 (0.50-0.89) | 16.22 (9.94 - 24.26) | 18.82 (11.30 - 28.24) | 0.72 (0.50-0.94) |
| Saint Lucia | 208.43 (159.92 - 268.60) | 231.80 (178.03 - 297.54) | 0.35 (0.32-0.37) | 44.13 (26.80 - 66.45) | 49.12 (30.25 - 73.63) | 0.35 (0.33-0.38) |
| Saint Vincent and the Grenadines | 180.35 (135.56 - 233.53) | 224.21 (169.48 - 288.65) | 0.86 (0.83-0.89) | 38.07 (23.57 - 58.04) | 47.41 (28.89 - 70.80) | 0.87 (0.84-0.90) |
| Samoa | 80.72 (60.03 - 105.35) | 92.82 (68.88 - 120.02) | 0.68 (0.61-0.74) | 17.16 (10.70 - 25.75) | 19.68 (12.11 - 29.67) | 0.68 (0.61-0.74) |
| Sao Tome and Principe | 95.94 (70.77 - 124.32) | 104.83 (78.57 - 135.22) | 0.36 (0.26-0.47) | 20.18 (12.53 - 30.60) | 22.17 (13.59 - 33.15) | 0.38 (0.28-0.49) |
| Saudi Arabia | 203.40 (154.04 - 260.41) | 231.92 (176.74 - 300.55) | 0.50 (0.42-0.58) | 42.76 (26.68 - 64.35) | 48.91 (30.64 - 73.35) | 0.51 (0.43-0.59) |
| Senegal | 93.08 (69.75 - 121.06) | 98.18 (72.15 - 127.25) | 0.25 (0.18-0.31) | 19.40 (11.97 - 29.34) | 20.63 (12.67 - 31.58) | 0.27 (0.20-0.33) |
| Serbia | 129.60 (97.13 - 165.55) | 143.46 (108.41 - 187.26) | 0.70 (0.51-0.90) | 27.47 (16.89 - 41.50) | 30.44 (18.78 - 45.21) | 0.70 (0.51-0.90) |
| Seychelles | 116.35 (87.65 - 149.59) | 142.30 (107.51 - 183.74) | 0.60 (0.54-0.67) | 24.75 (15.15 - 38.07) | 30.33 (18.83 - 45.54) | 0.61 (0.55-0.68) |
| Sierra Leone | 86.25 (63.98 - 112.45) | 85.99 (64.07 - 110.81) | -0.14 (-0.32 to 0.04) | 17.96 (11.02 - 27.34) | 18.08 (11.03 - 27.17) | -0.12 (-0.31 to 0.08) |
| Singapore | 262.71 (199.78 - 336.04) | 377.93 (290.01 - 487.66) | 1.34 (1.32-1.37) | 55.74 (34.84 - 84.09) | 80.37 (49.39 - 120.24) | 1.35 (1.32-1.38) |
| Slovakia | 144.84 (109.28 - 185.22) | 179.23 (134.80 - 232.34) | 1.00 (0.88-1.13) | 30.69 (19.32 - 46.10) | 38.01 (23.37 - 57.34) | 1.00 (0.88-1.13) |
| Slovenia | 158.98 (119.38 - 204.36) | 180.98 (137.11 - 234.88) | 0.71 (0.63-0.79) | 33.58 (20.62 - 49.43) | 38.27 (24.16 - 56.76) | 0.70 (0.62-0.78) |
| Solomon Islands | 64.66 (47.42 - 83.62) | 69.54 (50.70 - 90.01) | 0.05 (-0.05 to 0.16) | 13.66 (8.14 - 20.73) | 14.62 (9.08 - 21.96) | 0.06 (-0.05 to 0.16) |
| Somalia | 65.21 (47.96 - 85.49) | 61.91 (45.33 - 80.71) | -0.17 (-0.27 to -0.06) | 13.60 (8.21 - 20.70) | 13.04 (7.87 - 20.09) | -0.14 (-0.24 to -0.03) |
| South Africa | 156.71 (118.14 - 200.69) | 167.16 (125.91 - 215.27) | 0.38 (0.31-0.45) | 32.95 (20.65 - 49.45) | 35.03 (21.87 - 51.83) | 0.36 (0.29-0.43) |
| South Korea | 202.36 (155.03 - 259.92) | 290.93 (223.38 - 373.94) | 1.28 (1.20-1.37) | 42.88 (26.55 - 63.11) | 61.78 (39.21 - 91.91) | 1.29 (1.20-1.38) |
| South Sudan | 105.83 (77.81 - 139.19) | 105.68 (79.06 - 136.15) | 0.13 (0.06-0.21) | 21.83 (13.70 - 33.26) | 21.97 (13.56 - 32.97) | 0.17 (0.10-0.24) |
| Spain | 385.67 (300.81 - 495.4) | 501.50 (383.05 - 642.41) | 1.11 (1.03-1.19) | 81.30 (51.24 - 121.61) | 105.66 (65.76 - 161.17) | 1.11 (1.03-1.19) |
| Sri Lanka | 78.04 (57.85 - 101.84) | 114.40 (85.11 - 147.70) | 1.38 (1.32-1.43) | 16.47 (10.21 – 25.00) | 24.36 (15.02 - 36.82) | 1.41 (1.35-1.47) |
| Sudan | 92.19 (68.85 - 118.48) | 119.07 (89.24 - 153.55) | 1.08 (0.99-1.18) | 19.18 (11.82 - 28.84) | 24.93 (15.19 - 38.06) | 1.11 (1.01-1.20) |
| Suriname | 213.48 (162.64 - 276.03) | 252.3 (192.30 - 327.98) | 0.77 (0.65-0.89) | 45.16 (28.27 - 67.02) | 53.22 (33.08 - 79.52) | 0.77 (0.65-0.89) |
| Swaziland | 127.90 (95.43 - 165.53) | 147.99 (112.09 - 191.47) | 0.49 (0.46-0.52) | 26.86 (16.57 - 40.39) | 30.69 (18.89 - 45.95) | 0.43 (0.41-0.46) |
| Sweden | 337.55 (256.44 - 435.06) | 414.87 (316.91 - 533.44) | 0.81 (0.77-0.85) | 71.02 (44.39 - 107.34) | 87.09 (54.63 - 129.96) | 0.8 (0.76-0.84) |
| Switzerland | 346.83 (265.49 - 442.87) | 390.36 (300.49 - 500.22) | 0.45 (0.42-0.47) | 73.34 (46.70 - 108.67) | 82.57 (52.69 - 122.85) | 0.46 (0.43-0.48) |
| Syria | 106.96 (80.97 - 137.89) | 127.26 (95.08 - 162.77) | 1.06 (0.90-1.22) | 22.4 (13.95 - 33.83) | 26.64 (16.47 - 40.44) | 1.07 (0.91-1.23) |
| Taiwan (Province of China) | 117.82 (88.19 - 152.85) | 165.72 (124.61 - 211.73) | 1.20 (1.11-1.30) | 25.17 (15.36 - 37.69) | 35.29 (21.62 - 52.77) | 1.20 (1.10-1.29) |
| Tajikistan | 107.62 (80.47 - 138.54) | 91.67 (67.77 - 118.15) | -0.48 (-0.90 to -0.05) | 22.81 (13.90 - 34.21) | 19.49 (11.88 - 29.32) | -0.47 (-0.89 to -0.05) |
| Tanzania | 84.63 (62.06 - 109.84) | 100.84 (75.27 - 130.53) | 0.76 (0.62-0.90) | 17.68 (10.76 - 26.94) | 21.31 (12.94 - 32.15) | 0.81 (0.66-0.95) |
| Thailand | 89.79 (66.29 - 117.08) | 125.54 (94.01 - 161.11) | 1.13 (1.07-1.19) | 19.14 (11.67 - 29.12) | 26.67 (16.38 - 39.67) | 1.13 (1.08-1.19) |
| The Bahamas | 286.74 (220.43 - 368.14) | 292.78 (224.27 - 379.3) | 0.22 (0.14-0.29) | 60.78 (37.87 - 91.47) | 62.10 (38.92 - 92.59) | 0.21 (0.14-0.29) |
| The Gambia | 88.12 (65.32 - 115.23) | 88.38 (65.10 - 114.85) | 0.07 (0.04-0.10) | 18.32 (11.24 - 27.44) | 18.41 (11.17 - 28.01) | 0.08 (0.05-0.11) |
| Timor-Leste | 58.48 (42.77 - 76.97) | 81.69 (59.96 - 105.54) | 1.39 (1.26-1.53) | 12.21 (7.28 - 18.52) | 17.27 (10.55 - 26.18) | 1.45 (1.31-1.58) |
| Togo | 85.57 (63.67 - 111.35) | 85.04 (63.03 - 110.39) | -0.07 (-0.13 to 0.01) | 17.86 (10.70 - 27.25) | 17.83 (11.08 - 27.10) | -0.05 (-0.11 to 0.02) |
| Tonga | 82.25 (61.37 - 106.42) | 91.92 (68.49 - 118.93) | 0.39 (0.37-0.41) | 17.38 (10.41 - 26.40) | 19.4 (11.88 - 29.15) | 0.39 (0.37-0.42) |
| Trinidad and Tobago | 242.94 (185.84 - 313.61) | 313.17 (239.73 - 406.59) | 1.37 (1.20-1.55) | 51.32 (31.62 - 77.11) | 66.16 (41.40 - 99.38) | 1.37 (1.20-1.54) |
| Tunisia | 123.68 (92.08 - 158.37) | 157.70 (119.12 - 200.55) | 0.99 (0.96-1.03) | 25.98 (15.86 - 39.25) | 33.31 (20.51 - 50.50) | 1.01 (0.98-1.04) |
| Turkey | 177.43 (137.92 - 226.83) | 256.09 (196.12 - 332.09) | 1.41 (1.32-1.50) | 37.18 (23.86 - 55.47) | 54.01 (33.66 - 80.80) | 1.43 (1.34-1.52) |
| Turkmenistan | 128.62 (97.35 - 165.01) | 154.51 (117.32 - 200.19) | 0.79 (0.42-1.16) | 27.34 (16.61 - 41.44) | 32.94 (20.52 - 49.52) | 0.78 (0.42-1.15) |
| Uganda | 72.45 (54.28 - 92.86) | 93.14 (69.21 - 119.07) | 1.00 (0.96-1.05) | 14.91 (9.19 - 22.57) | 19.60 (11.91 - 29.58) | 1.08 (1.03-1.12) |
| Ukraine | 136.20 (100.86 - 175.46) | 131.03 (96.76 - 169.60) | 0.08 (-0.24 to 0.40) | 28.67 (17.74 - 43.36) | 27.63 (17.25 - 41.50) | 0.09 (-0.23 to 0.41) |
| United Arab Emirates | 233.27 (178.51 - 297.99) | 203.88 (157.10 - 263.88) | -0.53 (-0.64 to -0.42) | 49.09 (30.54 - 73.25) | 43.09 (26.41 - 64.44) | -0.52 (-0.63 to -0.41) |
| United Kingdom | 315.09 (241.82 - 400.35) | 377.87 (290.07 - 479.45) | 0.74 (0.67-0.81) | 66.12 (42.10 - 99.29) | 79.58 (50.61 - 119.22) | 0.75 (0.68-0.81) |
| United States | 319.39 (244.61 - 409.79) | 349.15 (266.98 - 450.10) | 0.41 (0.29-0.53) | 66.95 (42.46 - 101.09) | 73.32 (46.55 - 110.73) | 0.42 (0.29-0.54) |
| Uruguay | 236.23 (180.43 - 303.49) | 293.64 (222.48 - 381.61) | 0.69 (0.59-0.78) | 50.07 (30.39 - 74.93) | 62.07 (38.58 - 93.36) | 0.69 (0.59-0.78) |
| Uzbekistan | 102.20 (75.45 - 131.52) | 118.99 (89.35 - 152.91) | 0.63 (0.42-0.84) | 21.57 (13.53 - 32.14) | 25.21 (15.88 - 37.63) | 0.63 (0.43-0.84) |
| Vanuatu | 73.46 (54.08 - 95.26) | 78.50 (58.11 - 101.31) | 0.23 (0.20-0.26) | 15.44 (9.34 - 23.36) | 16.55 (10.12 - 24.83) | 0.24 (0.21-0.27) |
| Venezuela | 220.47 (169.49 - 286.84) | 226.64 (172.26 - 293.53) | 0.16 (0.08-0.25) | 46.92 (28.78 – 71.00) | 48.27 (30.54 - 72.96) | 0.16 (0.08-0.25) |
| Vietnam | 61.48 (44.87 - 80.20) | 93.43 (69.08 - 119.97) | 1.60 (1.56-1.64) | 13.01 (7.92 - 19.66) | 19.97 (12.12 - 30.02) | 1.63 (1.60-1.67) |
| Virgin Islands, U.S. | 275.36 (209.89 - 355.65) | 378.87 (289.43 - 493.32) | 1.31 (1.11-1.51) | 58.39 (35.87 - 88.38) | 80.44 (50.36 - 121.29) | 1.31 (1.11-1.51) |
| Yemen | 97.92 (72.49 - 126.21) | 108.52 (81.66 - 140.11) | 0.67 (0.55-0.79) | 20.01 (12.42 - 30.65) | 22.33 (13.89 - 33.97) | 0.71 (0.59-0.83) |
| Zambia | 97.63 (72.62 - 126.26) | 111.65 (82.56 - 144.47) | 0.64 (0.41-0.86) | 20.48 (12.74 - 30.73) | 23.53 (14.44 - 35.70) | 0.66 (0.43-0.89) |
| Zimbabwe | 108.45 (80.47 - 140.37) | 96.05 (71.11 - 124.07) | -0.90 (-1.09 to -0.72) | 22.68 (14.00 - 34.53) | 20.19 (12.38 - 30.66) | -0.87 (-1.05 to -0.69) |

DALYs, disability-adjusted life-years; ASR, age-standardized rate; EAPC, estimated annual percentage change; UI, uncertainty interval.
