# Supplementary material for: Trends in the prevalence and disability-adjusted life years of eating disorders from 1990 to 2017: results from the Global Burden of Disease Study 2017
Source: Epidemiol Psychiatr Sci. 2020 Dec 7;29:e191. doi: 10.1017/S2045796020001055 (PMC7737181; doi:10.1017/S2045796020001055)
Supplement: Supplementary file 1 [file S2045796020001055sup001.zip › Supplementary_Table_4.docx]

**Supplementary Table 4. Age-standardized rates of prevalence and disability-adjusted life-years of anorexia nervosa in 2017 and their temporal trend from 1990 to 2017 at national level.**

|  | **Prevalence (95% UI)** | | | **DALYs (95% UI)** | | |
| --- | --- | --- | --- | --- | --- | --- |
|  | **ASR in 1990**  **(per 100 000 population)** | **ASR in 2017**  **(per 100 000 population)** | **EAPC (%)** | **ASR in 2017**  **(per 100 000 population)** | **ASR in 1990**  **(per 100 000 population)** | **EAPC (%)** |
| Afghanistan | 18.89 (14.20 - 24.32) | 19.13 (14.35 - 24.99) | 0.23 (-0.03 to 0.48) | 3.88 (2.31 - 5.87) | 4.00 (2.36 - 6.17) | 0.30 (0.05-0.54) |
| Albania | 25.73 (19.27 - 33.45) | 32.73 (24.54 - 42.04) | 1.15 (1.04-1.27) | 5.51 (3.15 - 8.57) | 7.02 (4.18 - 10.85) | 1.17 (1.05-1.28) |
| Algeria | 31.65 (23.51 - 41.05) | 34.22 (25.51 - 44.17) | 0.46 (0.35-0.57) | 6.71 (3.94 - 10.40) | 7.33 (4.35 - 11.07) | 0.48 (0.37-0.58) |
| American Samoa | 45.19 (33.20 - 59.07) | 42.40 (30.98 - 55.12) | -0.16 (-0.20 to -0.12) | 9.82 (5.93 - 15.16) | 9.27 (5.71 - 13.90) | -0.13 (-0.18 to -0.08) |
| Andorra | 134.63 (100.73 - 176.15) | 146.2 (109.74 - 192.23) | 0.48 (0.40-0.56) | 28.89 (17.80 - 43.48) | 31.67 (19.34 - 47.04) | 0.51 (0.43-0.59) |
| Angola | 28.72 (21.46 - 37.52) | 35.47 (26.41 - 45.43) | 0.98 (0.77-1.19) | 6.03 (3.46 - 9.43) | 7.53 (4.39 - 11.67) | 1.01 (0.79-1.22) |
| Antigua and Barbuda | 44.37 (32.83 - 57.60) | 50.77 (37.67 – 66.00) | 0.50 (0.44-0.56) | 9.46 (5.50 - 14.43) | 10.82 (6.33 - 16.54) | 0.50 (0.44-0.56) |
| Argentina | 64.59 (47.82 - 84.35) | 78.88 (58.75 - 103.42) | 0.65 (0.58-0.72) | 13.92 (8.14 - 21.48) | 16.82 (10.14 - 25.87) | 0.57 (0.48-0.66) |
| Armenia | 27.37 (20.54 - 35.47) | 31.67 (23.60 - 40.92) | 0.96 (0.68-1.25) | 5.94 (3.57 - 9.06) | 6.89 (4.06 - 10.50) | 0.94 (0.67-1.21) |
| Australia | 90.69 (66.39 - 117.29) | 113.01 (84.65 - 147.89) | 0.96 (0.88-1.04) | 19.75 (12.50 - 29.65) | 24.31 (14.87 - 36.81) | 0.87 (0.80-0.93) |
| Austria | 108.87 (81.17 - 141.68) | 135.54 (100.15 - 176.55) | 0.90 (0.81-0.98) | 23.22 (13.93 - 36.04) | 30.79 (19.47 - 45.80) | 1.20 (1.09-1.30) |
| Azerbaijan | 32.20 (24.37 - 41.58) | 38.36 (28.29 - 49.57) | 1.00 (0.52-1.48) | 6.99 (4.20 - 10.73) | 8.32 (4.86 - 12.75) | 1.00 (0.52-1.49) |
| Bahrain | 39.08 (29.01 - 51.11) | 43.23 (31.74 - 56.79) | 0.26 (0.21-0.32) | 8.32 (4.75 - 12.64) | 9.22 (5.37 - 13.82) | 0.27 (0.22-0.33) |
| Bangladesh | 21.97 (16.21 - 28.64) | 28.31 (20.96 - 37.11) | 0.90 (0.80-1.01) | 4.65 (2.70 - 7.11) | 6.03 (3.54 - 9.23) | 0.94 (0.83-1.04) |
| Barbados | 44.22 (32.74 - 57.21) | 46.03 (34.50 - 59.85) | 0.22 (0.16-0.29) | 9.49 (5.53 - 14.64) | 9.89 (5.67 - 14.73) | 0.21 (0.15-0.27) |
| Belarus | 33.28 (24.79 - 43.01) | 39.53 (29.59 - 51.18) | 0.85 (0.61-1.08) | 7.11 (4.23 - 11.15) | 8.52 (4.98 - 12.96) | 0.89 (0.65-1.12) |
| Belgium | 111.32 (83.08 - 146.35) | 128.65 (96.32 - 168.10) | 0.58 (0.56-0.60) | 24.09 (14.72 - 36.13) | 27.94 (17.02 - 42.52) | 0.65 (0.59-0.72) |
| Belize | 32.13 (23.74 - 42.29) | 38.36 (28.77 - 49.98) | 0.59 (0.52-0.65) | 6.84 (3.84 - 10.75) | 8.20 (4.84 - 12.55) | 0.57 (0.50-0.64) |
| Benin | 23.95 (17.75 - 31.03) | 25.25 (18.81 - 32.80) | 0.30 (0.25-0.34) | 5.02 (2.92 - 7.70) | 5.39 (3.07 - 8.49) | 0.34 (0.29-0.39) |
| Bermuda | 60.66 (44.77 - 78.81) | 69.57 (51.47 - 91.51) | 0.68 (0.61-0.75) | 13.02 (7.91 - 19.83) | 14.93 (8.72 - 22.64) | 0.67 (0.60-0.73) |
| Bhutan | 24.22 (18.04 - 31.60) | 33.63 (24.85 - 43.49) | 1.24 (1.21-1.28) | 5.13 (2.95 - 7.92) | 7.18 (4.23 - 11.08) | 1.28 (1.24-1.31) |
| Bolivia | 28.60 (21.30 - 37.20) | 33.89 (24.98 - 44.07) | 0.60 (0.54-0.65) | 6.08 (3.47 - 9.68) | 7.28 (4.22 - 11.44) | 0.61 (0.55-0.68) |
| Bosnia and Herzegovina | 20.34 (15.12 - 26.27) | 32.48 (23.99 - 42.44) | 2.14 (1.90-2.38) | 4.35 (2.51 - 6.84) | 6.98 (4.26 - 10.78) | 2.15 (1.91-2.39) |
| Botswana | 35.01 (25.92 - 45.69) | 44.34 (33.00 - 58.06) | 0.86 (0.84-0.88) | 7.45 (4.4 - 11.54) | 9.43 (5.75 - 14.35) | 0.87 (0.85-0.89) |
| Brazil | 40.88 (30.75 - 52.47) | 46.95 (35.08 - 60.94) | 0.54 (0.50-0.58) | 8.75 (5.31 - 12.94) | 10.11 (6.22 - 14.87) | 0.56 (0.53-0.60) |
| Brunei | 122.27 (91.58 - 159.51) | 122.02 (91.13 - 158.93) | 0.13 (0.08-0.18) | 26.67 (16.21 - 40.34) | 28.43 (17.83 - 41.44) | 0.38 (0.34-0.43) |
| Bulgaria | 33.39 (24.75 - 43.88) | 37.60 (28.03 - 48.64) | 0.57 (0.38-0.76) | 7.17 (4.25 - 11.09) | 8.14 (4.84 - 12.36) | 0.60 (0.41-0.79) |
| Burkina Faso | 20.97 (15.64 - 27.22) | 24.2 (17.93 - 31.33) | 0.57 (0.53-0.61) | 4.42 (2.56 - 6.89) | 5.17 (3.00 - 8.16) | 0.64 (0.58-0.69) |
| Burundi | 20.73 (15.51 - 26.82) | 19.74 (14.71 - 25.61) | -0.27 (-0.36 to -0.18) | 4.41 (2.50 - 6.82) | 4.21 (2.49 - 6.51) | -0.27 (-0.37 to -0.17) |
| Cambodia | 20.66 (15.37 - 26.76) | 27.28 (20.38 - 35.37) | 1.12 (1.02-1.22) | 4.39 (2.56 - 6.83) | 5.87 (3.45 – 9.00) | 1.17 (1.07-1.27) |
| Cameroon | 28.52 (20.95 - 37.11) | 27.85 (20.66 - 36.06) | -0.01 (-0.11 to 0.09) | 6.00 (3.48 - 9.21) | 5.92 (3.45 - 9.23) | 0.03 (-0.07 to 0.13) |
| Canada | 90.79 (67.61 - 118.28) | 98.76 (72.80 - 130.43) | 0.39 (0.35-0.42) | 19.68 (11.89 - 29.74) | 21.66 (13.29 - 32.89) | 0.43 (0.39-0.47) |
| Cape Verde | 25.60 (19.06 - 33.24) | 33.40 (24.66 - 43.50) | 1.16 (1.10-1.21) | 5.43 (3.19 - 8.34) | 7.14 (4.17 - 11.09) | 1.19 (1.13-1.25) |
| Central African Republic | 21.09 (15.77 - 27.4) | 19.46 (14.40 - 25.29) | -0.20 (-0.23 to -0.17) | 4.40 (2.57 - 6.78) | 4.14 (2.39 - 6.47) | -0.15 (-0.18 to -0.12) |
| Chad | 22.51 (16.57 - 29.03) | 25.93 (19.38 - 33.59) | 0.60 (0.50-0.70) | 4.72 (2.79 - 7.16) | 5.48 (3.12 - 8.48) | 0.62 (0.52-0.71) |
| Chile | 60.27 (44.78 - 78.82) | 80.96 (59.25 - 106.12) | 1.04 (1.01-1.06) | 12.94 (7.64 - 19.45) | 17.17 (9.95 - 26.03) | 0.99 (0.95-1.02) |
| China | 23.06 (16.85 - 30.84) | 39.47 (28.65 - 53.68) | 2.14 (2.09-2.20) | 5.00 (3.04 - 7.58) | 8.65 (5.12 - 13.16) | 2.18 (2.12-2.24) |
| Colombia | 36.05 (27.22 - 46.47) | 42.43 (31.01 - 55.27) | 0.56 (0.48-0.64) | 7.79 (4.59 - 11.77) | 9.12 (5.29 - 14.30) | 0.57 (0.49-0.64) |
| Comoros | 24.47 (18.20 - 31.66) | 23.01 (17.14 - 29.81) | -0.2 (-0.24 to -0.16) | 5.19 (2.94 - 8.13) | 4.91 (2.82 - 7.50) | -0.18 (-0.22 to -0.15) |
| Congo | 30.59 (22.98 - 39.94) | 32.93 (24.22 - 42.81) | 0.34 (0.23-0.44) | 6.42 (3.86 - 10.07) | 7.01 (4.11 - 10.89) | 0.38 (0.28-0.49) |
| Costa Rica | 36.06 (26.99 - 47.18) | 44.71 (33.04 - 57.87) | 0.81 (0.78-0.85) | 7.79 (4.42 - 11.93) | 9.59 (5.63 - 14.77) | 0.82 (0.78-0.85) |
| Cote d'Ivoire | 28.16 (20.75 - 36.95) | 28.05 (21.00 - 36.40) | -0.08 (-0.17 to 0.01) | 5.91 (3.42 - 9.13) | 5.98 (3.56 - 9.51) | -0.04 (-0.13 to 0.05) |
| Croatia | 36.12 (27.14 - 46.25) | 40.04 (29.78 - 52.43) | 0.65 (0.54-0.76) | 7.74 (4.68 - 11.85) | 8.70 (5.19 - 13.32) | 0.73 (0.60-0.85) |
| Cuba | 34.64 (25.34 - 44.74) | 37.98 (28.54 - 49.10) | 0.58 (0.39-0.78) | 7.51 (4.50 - 11.57) | 8.24 (4.78 - 12.58) | 0.57 (0.38-0.76) |
| Cyprus | 98.07 (73.30 - 127.75) | 114.26 (85.13 - 149.3) | 0.71 (0.63-0.80) | 20.83 (12.28 - 31.88) | 24.28 (14.88 - 37.46) | 0.73 (0.64-0.82) |
| Czech Republic | 39.28 (29.14 - 51.11) | 44.27 (32.83 - 58.07) | 0.58 (0.51-0.64) | 8.45 (4.89 - 13.10) | 9.53 (5.72 - 14.69) | 0.60 (0.54-0.66) |
| Democratic Republic of the Congo | 22.96 (16.91 - 29.61) | 19.91 (14.78 - 25.73) | -0.69 (-0.98 to -0.41) | 4.81 (2.79 - 7.32) | 4.21 (2.41 - 6.50) | -0.64 (-0.92 to -0.35) |
| Denmark | 111.98 (84.99 - 146.13) | 116.86 (86.44 - 153.04) | 0.37 (0.29-0.45) | 23.94 (14.61 - 35.64) | 25.07 (15.28 - 38.14) | 0.34 (0.25-0.44) |
| Djibouti | 27.51 (20.32 - 35.77) | 27.03 (20.03 - 35.30) | -0.05 (-0.18 to 0.09) | 5.87 (3.35 - 9.13) | 5.84 (3.42 - 9.05) | -0.03 (-0.18 to 0.12) |
| Dominica | 34.54 (25.87 - 44.87) | 40.46 (30.40 - 52.85) | 0.61 (0.59-0.62) | 7.45 (4.35 - 11.54) | 8.64 (5.10 - 13.28) | 0.59 (0.57-0.61) |
| Dominican Republic | 35.16 (26.25 - 45.40) | 44.23 (32.67 - 57.67) | 0.88 (0.85-0.91) | 7.66 (4.67 - 11.72) | 9.83 (5.94 - 14.91) | 0.93 (0.90-0.96) |
| Ecuador | 34.59 (25.75 - 45.27) | 39.13 (29.30 - 50.94) | 0.47 (0.39-0.55) | 7.41 (4.36 - 11.41) | 8.41 (4.84 - 12.73) | 0.48 (0.40-0.56) |
| Egypt | 25.30 (19.03 - 32.47) | 31.67 (23.58 - 41.13) | 0.88 (0.86-0.90) | 5.36 (3.16 - 8.27) | 6.76 (4.03 - 10.27) | 0.90 (0.87-0.93) |
| El Salvador | 29.77 (22.17 - 38.53) | 38.54 (28.76 - 50.29) | 0.99 (0.97-1.01) | 6.42 (3.77 - 9.9) | 8.35 (5.00 - 12.82) | 1.01 (0.99-1.04) |
| Equatorial Guinea | 20.82 (15.66 - 26.99) | 52.94 (39.63 - 69.69) | 4.65 (4.00-5.31) | 4.39 (2.60 - 6.87) | 11.26 (6.61 - 17.15) | 4.70 (4.05-5.36) |
| Eritrea | 18.89 (14.01 - 24.50) | 21.51 (15.93 - 27.75) | 0.33 (0.15-0.52) | 3.94 (2.26 - 6.24) | 4.59 (2.64 - 7.18) | 0.42 (0.23-0.61) |
| Estonia | 52.97 (39.75 - 68.43) | 51.86 (39.12 - 68.11) | 0.16 (0.04-0.28) | 15.04 (10.19 - 21.29) | 14.22 (9.73 - 20.46) | -0.22 (-0.32 to -0.12) |
| Ethiopia | 18.57 (13.93 - 24.23) | 22.89 (17.11 - 29.61) | 0.82 (0.58-1.06) | 3.92 (2.35 - 5.88) | 4.89 (2.97 - 7.29) | 0.88 (0.64-1.12) |
| Federated States of Micronesia | 25.47 (18.98 - 33.13) | 27.11 (20.19 - 35.51) | 0.21 (0.19-0.22) | 5.49 (3.16 - 8.52) | 5.93 (3.46 - 9.29) | 0.25 (0.23-0.27) |
| Fiji | 31.49 (23.76 - 40.26) | 35.75 (26.77 - 47.03) | 0.40 (0.38-0.42) | 6.78 (4.06 - 10.33) | 7.79 (4.81 - 11.95) | 0.44 (0.41-0.47) |
| Finland | 147.45 (112.79 - 189.69) | 159.39 (116.96 - 208.67) | 0.42 (0.34-0.49) | 31.72 (19.20 - 46.61) | 34.55 (20.92 - 51.96) | 0.45 (0.37-0.53) |
| France | 116.54 (87.07 - 150.75) | 127.80 (95.14 - 167.91) | 0.37 (0.34-0.39) | 26.02 (16.34 - 38.95) | 28.32 (17.63 - 42.55) | 0.34 (0.32-0.36) |
| Gabon | 44.01 (32.50 - 57.36) | 45.72 (33.92 - 59.82) | 0.11 (0.08-0.14) | 9.29 (5.38 - 14.30) | 9.73 (5.70 - 14.37) | 0.13 (0.10-0.17) |
| Georgia | 32.63 (24.59 - 42.55) | 32.17 (24.15 - 42.37) | 0.27 (-0.14 to 0.68) | 7.11 (4.19 - 11.02) | 7.01 (4.13 - 10.75) | 0.25 (-0.15 to 0.66) |
| Germany | 138.23 (105.34 - 176.11) | 151.75 (114.55 - 196.62) | 0.29 (0.21-0.37) | 30.16 (18.77 - 44.84) | 33.59 (20.87 - 50.15) | 0.34 (0.26-0.42) |
| Ghana | 24.87 (18.69 - 32.77) | 30.39 (22.56 - 39.34) | 0.69 (0.60-0.77) | 5.29 (3.03 - 8.21) | 6.50 (3.83 - 10.06) | 0.70 (0.61-0.79) |
| Greece | 107.21 (79.21 - 137.37) | 116.02 (85.69 - 153.52) | 0.50 (0.41-0.60) | 22.69 (13.33 - 34.25) | 25.13 (15.64 - 37.09) | 0.62 (0.51-0.73) |
| Greenland | 101.31 (75.30 - 131.92) | 116.33 (85.80 - 153.84) | 0.62 (0.55-0.69) | 21.61 (13.07 - 32.80) | 24.93 (15.21 - 37.68) | 0.64 (0.57-0.71) |
| Grenada | 34.00 (25.36 - 43.70) | 41.99 (31.43 - 54.58) | 0.88 (0.82-0.94) | 7.29 (4.31 - 11.17) | 9.01 (5.23 - 13.94) | 0.87 (0.82-0.92) |
| Guam | 55.08 (40.98 - 72.85) | 58.97 (43.64 - 76.60) | 0.29 (0.26-0.32) | 12.08 (7.20 - 18.32) | 12.98 (8.00 - 19.62) | 0.30 (0.27-0.33) |
| Guatemala | 32.18 (23.83 - 41.46) | 36.80 (27.32 - 47.81) | 0.52 (0.50-0.54) | 6.87 (4.16 - 10.44) | 7.92 (4.68 - 12.21) | 0.54 (0.52-0.57) |
| Guinea | 24.10 (17.85 - 31.12) | 24.06 (17.86 - 30.95) | -0.04 (-0.09 to 0.02) | 5.08 (2.95 - 7.80) | 5.12 (2.89 - 7.91) | 0.01 (-0.05 to 0.05) |
| Guinea-Bissau | 23.43 (17.26 - 30.52) | 23.57 (17.42 - 30.41) | -0.04 (-0.07 to -0.01) | 4.96 (2.83 - 7.64) | 5.02 (2.91 - 7.76) | -0.02 (-0.06 to 0.02) |
| Guyana | 28.61 (21.50 - 37.16) | 35.74 (26.53 - 46.47) | 0.78 (0.73-0.83) | 6.09 (3.51 - 9.51) | 7.6 (4.55 - 11.53) | 0.78 (0.72-0.83) |
| Haiti | 26.30 (19.46 - 34.27) | 25.65 (19.15 - 33.30) | -0.04 (-0.07 to 0.01) | 5.57 (3.30 - 8.60) | 5.43 (3.20 - 8.30) | -0.05 (-0.09 to -0.01) |
| Honduras | 28.87 (21.51 - 37.53) | 33.03 (24.64 - 42.67) | 0.55 (0.51-0.60) | 6.23 (3.64 - 9.67) | 7.17 (4.24 - 11.16) | 0.58 (0.53-0.63) |
| Hungary | 35.05 (26.10 - 45.12) | 41.86 (31.33 - 54.33) | 0.85 (0.75-0.94) | 7.5 (4.49 - 11.38) | 9.05 (5.45 - 13.84) | 0.90 (0.80-1.00) |
| Iceland | 112.76 (84.69 - 145.97) | 126.54 (94.5 - 165.28) | 0.52 (0.48-0.55) | 24.27 (14.9 - 36.91) | 26.86 (16.06 - 40.97) | 0.48 (0.45-0.52) |
| India | 22.07 (16.37 - 28.65) | 30.78 (23.04 - 39.49) | 1.25 (1.16-1.34) | 4.67 (2.87 - 6.97) | 6.56 (4.06 - 9.79) | 1.28 (1.19-1.37) |
| Indonesia | 27.14 (20.15 - 35.14) | 35.60 (26.55 - 45.78) | 0.85 (0.77-0.94) | 5.81 (3.49 - 8.68) | 7.66 (4.69 - 11.47) | 0.87 (0.78-0.95) |
| Iran | 30.25 (22.69 - 38.72) | 36.98 (27.80 - 47.47) | 0.91 (0.84-0.97) | 6.49 (4.04 - 9.56) | 8.17 (5.13 - 12.01) | 1.03 (0.96-1.10) |
| Iraq | 30.43 (22.51 - 39.32) | 34.16 (25.45 - 44.42) | 0.73 (0.53-0.92) | 6.43 (3.83 - 9.81) | 7.27 (4.39 - 11.42) | 0.75 (0.55-0.94) |
| Ireland | 82.84 (61.17 - 106.56) | 117.40 (87.66 - 151.82) | 1.41 (1.32-1.49) | 17.58 (10.54 - 26.37) | 24.93 (14.95 - 37.42) | 1.39 (1.30-1.48) |
| Israel | 72.21 (54.22 - 93.51) | 90.74 (66.62 – 119.00) | 0.91 (0.85-0.98) | 15.34 (9.22 - 23.33) | 19.28 (11.28 - 29.49) | 0.92 (0.86-0.98) |
| Italy | 122.58 (93.05 - 155.93) | 128.43 (96.54 - 165.25) | 0.16 (0.09-0.23) | 27.13 (17.18 - 40.30) | 27.67 (17.12 - 41.56) | 0.04 (-0.01 to 0.09) |
| Jamaica | 35.83 (26.76 - 46.17) | 38.26 (28.54 - 49.62) | 0.21 (0.19-0.23) | 7.69 (4.43 - 11.53) | 8.20 (4.82 - 12.51) | 0.20 (0.17-0.22) |
| Japan | 96.92 (73.11 - 124.67) | 104.93 (79.00 - 135.81) | 0.33 (0.16-0.50) | 21.8 (13.69 - 31.99) | 24.56 (15.88 - 35.62) | 0.40 (0.18-0.62) |
| Jordan | 28.83 (21.50 - 37.24) | 31.63 (23.62 - 40.98) | 0.57 (0.46-0.69) | 6.12 (3.63 - 9.41) | 6.76 (4.00 - 10.41) | 0.59 (0.48-0.70) |
| Kazakhstan | 35.64 (25.97 - 46.76) | 42.76 (32.07 - 55.22) | 0.90 (0.64-1.15) | 7.70 (4.61 - 11.75) | 9.25 (5.52 - 14.08) | 0.88 (0.63-1.13) |
| Kenya | 25.53 (19.10 - 32.92) | 27.29 (20.33 - 35.07) | 0.20 (0.12-0.27) | 5.46 (3.28 - 8.21) | 5.85 (3.56 - 8.73) | 0.21 (0.13-0.29) |
| Kiribati | 24.25 (17.99 - 31.62) | 23.87 (17.87 - 31.04) | -0.06 (-0.09 to -0.03) | 5.18 (3.03 - 8.03) | 5.16 (2.95 - 7.97) | -0.04 (-0.08 to 0.01) |
| Kuwait | 47.58 (34.78 - 62.08) | 55.06 (41.28 - 72.85) | 0.61 (0.53-0.70) | 10.56 (6.53 - 15.86) | 12.33 (7.49 - 18.49) | 0.59 (0.51-0.67) |
| Kyrgyzstan | 28.32 (21.00 - 36.11) | 25.16 (18.79 - 32.16) | -0.43 (-0.67 to -0.18) | 6.42 (3.98 - 9.70) | 5.71 (3.44 - 8.65) | -0.46 (-0.67 to -0.25) |
| Laos | 22.84 (17.05 - 29.41) | 31.46 (23.54 - 40.57) | 1.19 (1.08-1.30) | 4.86 (2.81 - 7.45) | 6.71 (3.8 - 10.44) | 1.21 (1.11-1.31) |
| Latvia | 37.65 (28.27 - 48.82) | 42.04 (31.46 - 55.08) | 0.76 (0.53-0.99) | 8.11 (4.96 - 12.39) | 9.07 (5.38 - 14.09) | 0.76 (0.53-0.99) |
| Lebanon | 31.64 (23.42 - 41.09) | 35.23 (26.56 - 46.13) | 0.58 (0.50-0.66) | 6.74 (3.98 - 10.43) | 7.57 (4.38 - 11.87) | 0.61 (0.53-0.70) |
| Lesotho | 22.61 (16.86 - 29.29) | 27.71 (20.72 - 35.76) | 0.73 (0.70-0.76) | 4.80 (2.76 - 7.43) | 5.84 (3.41 – 9.00) | 0.72 (0.68-0.75) |
| Liberia | 21.48 (15.85 - 28.24) | 19.92 (14.84 - 26.01) | 0.09 (-0.17 to 0.35) | 4.50 (2.61 - 6.93) | 4.17 (2.39 - 6.47) | 0.09 (-0.17 to 0.36) |
| Libya | 39.64 (29.70 - 51.28) | 31.93 (23.90 - 41.56) | -0.35 (-0.54 to -0.16) | 8.47 (4.99 - 12.88) | 6.86 (4.00 - 10.83) | -0.33 (-0.52 to -0.15) |
| Lithuania | 36.85 (27.40 - 48.47) | 43.65 (32.02 - 56.55) | 0.88 (0.68-1.09) | 7.87 (4.54 - 12.24) | 9.30 (5.48 - 14.17) | 0.89 (0.68-1.09) |
| Luxembourg | 143.98 (110.43 - 185.97) | 175.57 (132.16 - 227.4) | 0.74 (0.63-0.86) | 32.74 (21.04 - 49.10) | 39.96 (25.39 - 58) | 0.76 (0.67-0.85) |
| Macedonia | 31.78 (23.64 - 41.24) | 34.37 (25.59 - 44.55) | 0.40 (0.25-0.54) | 6.84 (3.89 - 10.69) | 7.41 (4.41 - 11.18) | 0.41 (0.27-0.55) |
| Madagascar | 23.06 (17.12 - 29.82) | 22.5 (16.88 - 29.36) | -0.11 (-0.16 to -0.05) | 4.88 (2.90 - 7.51) | 4.80 (2.80 - 7.51) | -0.09 (-0.14 to -0.03) |
| Malawi | 20.56 (15.30 - 26.85) | 21.55 (16.28 - 27.94) | 0.24 (0.18-0.30) | 4.35 (2.46 - 6.79) | 4.59 (2.58 - 7.14) | 0.28 (0.21-0.35) |
| Malaysia | 35.63 (26.36 - 46.35) | 46.19 (34.11 - 59.98) | 0.93 (0.90-0.96) | 7.65 (4.39 - 11.77) | 9.97 (5.84 - 15.03) | 0.94 (0.91-0.98) |
| Maldives | 29.37 (21.82 - 37.88) | 35.87 (26.54 - 46.63) | 0.89 (0.75-1.02) | 6.23 (3.67 - 9.50) | 7.72 (4.67 - 11.97) | 0.94 (0.80-1.08) |
| Mali | 21.57 (16.10 - 27.71) | 24.88 (18.41 - 32.33) | 0.58 (0.56-0.60) | 4.52 (2.69 - 6.97) | 5.30 (3.10 - 8.26) | 0.62 (0.60-0.64) |
| Malta | 91.58 (67.67 - 118.95) | 117.45 (86.78 - 153.38) | 0.91 (0.86-0.96) | 20.01 (12.48 - 30.05) | 25.62 (15.99 - 37.97) | 0.91 (0.86-0.95) |
| Marshall Islands | 25.77 (19.32 - 33.14) | 27.84 (20.75 - 35.96) | 0.22 (0.19-0.25) | 5.61 (3.31 - 8.57) | 6.14 (3.52 - 9.36) | 0.27 (0.24-0.30) |
| Mauritania | 27.17 (20.21 - 35.55) | 30.05 (22.35 - 39.10) | 0.39 (0.31-0.47) | 5.77 (3.29 - 8.84) | 6.45 (3.73 - 10.02) | 0.42 (0.34-0.50) |
| Mauritius | 33.02 (24.61 - 42.77) | 43.71 (32.19 - 57.41) | 0.97 (0.93-1.01) | 7.05 (4.16 - 10.86) | 9.41 (5.48 - 14.41) | 0.97 (0.93-1.01) |
| Mexico | 40.94 (30.66 - 53.03) | 45.05 (33.71 – 58.00) | 0.40 (0.38-0.43) | 8.86 (5.42 - 13.20) | 9.79 (5.93 - 14.52) | 0.39 (0.37-0.42) |
| Moldova | 32.24 (24.40 - 41.15) | 28.65 (21.50 - 36.77) | -0.34 (-0.63 to -0.05) | 7.67 (4.86 - 11.47) | 7.09 (4.64 - 10.49) | -0.26 (-0.5 to -0.02) |
| Mongolia | 26.76 (19.98 - 34.48) | 34.29 (25.60 - 44.78) | 0.98 (0.80-1.16) | 5.81 (3.40 - 8.75) | 7.42 (4.59 - 11.50) | 0.97 (0.79-1.16) |
| Montenegro | 33.27 (25.00 - 42.91) | 36.10 (27.04 - 46.39) | 0.56 (0.39-0.72) | 7.13 (4.16 - 11.03) | 7.79 (4.70 - 11.97) | 0.57 (0.41-0.73) |
| Morocco | 24.47 (18.18 - 31.54) | 28.99 (21.46 - 37.51) | 0.62 (0.57-0.66) | 5.16 (3.07 - 7.86) | 6.18 (3.54 - 9.55) | 0.64 (0.59-0.68) |
| Mozambique | 17.33 (12.97 - 22.49) | 22.33 (16.62 - 29.42) | 1.09 (1.02-1.16) | 3.68 (2.14 - 5.76) | 4.72 (2.71 - 7.25) | 1.09 (1.02-1.16) |
| Myanmar | 19.85 (14.80 - 25.83) | 31.17 (23.38 - 40.56) | 1.89 (1.72-2.06) | 4.21 (2.53 - 6.62) | 6.69 (3.91 - 10.47) | 1.94 (1.77-2.11) |
| Namibia | 34.02 (25.23 - 43.9) | 39.69 (29.43 - 52.15) | 0.61 (0.50-0.71) | 7.21 (4.07 - 11.31) | 8.47 (5.11 - 13.08) | 0.62 (0.51-0.73) |
| Nepal | 21.32 (15.73 - 28.2) | 26.46 (19.80 - 34.30) | 0.76 (0.71-0.82) | 4.49 (2.53 - 6.98) | 5.64 (3.15 - 8.77) | 0.83 (0.78-0.88) |
| Netherlands | 97.70 (75.54 - 124.55) | 142.71 (108.28 - 184.95) | 1.66 (1.52-1.79) | 20.86 (12.72 - 30.99) | 32.44 (20.59 - 47.98) | 1.98 (1.85-2.11) |
| New Zealand | 138.87 (102.33 - 179.74) | 142.34 (106.22 - 186.19) | 0.26 (0.19-0.33) | 29.55 (18.01 - 44.56) | 30.40 (18.76 - 45.80) | 0.30 (0.21-0.39) |
| Nicaragua | 30.28 (22.41 - 39.54) | 32.59 (24.30 - 42.12) | 0.40 (0.31-0.49) | 6.48 (3.78 - 9.99) | 7.09 (4.24 - 10.72) | 0.46 (0.37-0.54) |
| Niger | 21.08 (15.74 - 27.2) | 20.83 (15.37 - 26.95) | -0.06 (-0.16 to 0.04) | 4.45 (2.53 - 7.02) | 4.44 (2.55 - 6.87) | -0.04 (-0.13 to 0.06) |
| Nigeria | 27.39 (20.27 - 35.83) | 34.03 (25.07 - 44.02) | 1.13 (0.87-1.39) | 5.81 (3.35 - 8.97) | 7.23 (4.24 - 10.99) | 1.16 (0.89-1.42) |
| North Korea | 31.21 (23.00 - 40.22) | 23.82 (17.64 - 30.89) | -1.15 (-1.36 to -0.93) | 6.71 (3.84 - 10.28) | 5.15 (2.94 - 7.95) | -1.14 (-1.35 to -0.92) |
| Northern Mariana Islands | 60.85 (44.46 - 81.92) | 46.32 (34.41 - 60.40) | -1.01 (-1.15 to -0.88) | 13.26 (7.94 - 20.29) | 10.11 (6.11 - 15.35) | -1.01 (-1.15 to -0.87) |
| Norway | 107.67 (81.54 - 138.93) | 114.40 (86.29 - 148.08) | 0.20 (0.15-0.25) | 24.2 (15.45 - 35.28) | 24.7 (15.34 - 36.64) | 0.09 (0.05-0.13) |
| Oman | 34.95 (26.11 - 45.50) | 39.20 (28.79 - 51.05) | 0.50 (0.31-0.68) | 7.42 (4.45 - 11.48) | 8.41 (4.92 - 12.79) | 0.51 (0.33-0.70) |
| Pakistan | 26.01 (19.17 - 33.67) | 30.60 (22.75 - 39.54) | 0.60 (0.56-0.64) | 5.51 (3.31 - 8.52) | 6.56 (3.83 - 10.10) | 0.63 (0.59-0.67) |
| Palestine | 21.29 (15.98 - 27.56) | 24.15 (18.20 - 31.14) | 0.36 (0.29-0.42) | 4.52 (2.70 - 6.92) | 5.19 (2.95 - 7.93) | 0.38 (0.31-0.45) |
| Panama | 37.15 (27.54 - 48.23) | 49.20 (36.35 - 63.85) | 1.06 (0.99-1.13) | 8.25 (4.9 - 12.70) | 11.14 (6.83 - 16.49) | 1.12 (1.06-1.18) |
| Papua New Guinea | 23.43 (17.42 - 30.53) | 26.29 (19.51 - 34.04) | 0.26 (0.18-0.34) | 4.98 (2.96 - 7.64) | 5.61 (3.30 - 8.62) | 0.27 (0.19-0.35) |
| Paraguay | 36.60 (27.43 - 47.56) | 41.41 (30.94 - 53.40) | 0.38 (0.31-0.45) | 8.00 (4.81 - 12.06) | 9.24 (5.56 - 13.97) | 0.46 (0.39-0.53) |
| Peru | 33.73 (25.03 - 44.07) | 40.17 (29.7 - 52.49) | 0.76 (0.63-0.88) | 7.32 (4.43 - 11.20) | 8.75 (5.12 - 13.37) | 0.76 (0.64-0.89) |
| Philippines | 28.11 (20.73 - 36.43) | 32.95 (24.04 - 43.16) | 0.52 (0.45-0.60) | 6.03 (3.52 - 9.27) | 7.10 (4.16 - 10.96) | 0.54 (0.47-0.62) |
| Poland | 32.37 (24.13 - 41.56) | 42.76 (31.85 - 55.02) | 1.16 (1.12-1.21) | 7.09 (4.24 - 10.86) | 9.63 (5.79 - 14.34) | 1.33 (1.27-1.40) |
| Portugal | 99.91 (75.25 - 128.19) | 119.08 (87.20 - 157.49) | 0.62 (0.55-0.68) | 21.14 (12.64 - 32.12) | 25.25 (15.23 - 38.45) | 0.61 (0.54-0.68) |
| Puerto Rico | 52.14 (38.95 - 68.18) | 58.63 (43.15 - 76.77) | 0.51 (0.41-0.61) | 11.53 (6.88 - 17.69) | 12.93 (7.78 - 19.89) | 0.47 (0.37-0.57) |
| Qatar | 43.99 (32.91 - 57.54) | 45.60 (33.64 - 59.22) | 0.28 (0.10-0.46) | 9.39 (5.64 - 14.51) | 9.75 (5.65 - 14.8) | 0.28 (0.11-0.46) |
| Romania | 32.65 (24.27 - 42.06) | 39.17 (29.09 - 50.81) | 0.87 (0.70-1.05) | 6.97 (4.08 - 10.58) | 8.43 (4.95 - 13.01) | 0.90 (0.73-1.08) |
| Russian Federation | 40.29 (30.33 - 51.58) | 42.79 (32.24 - 55.14) | 0.38 (0.12-0.64) | 8.74 (5.36 - 12.98) | 9.27 (5.78 - 13.75) | 0.35 (0.08-0.62) |
| Rwanda | 21.15 (15.74 - 27.50) | 24.09 (17.80 - 31.35) | 0.58 (0.42-0.74) | 4.52 (2.53 - 6.86) | 5.12 (2.89 - 7.98) | 0.60 (0.43-0.78) |
| Saint Lucia | 38.08 (28.42 - 49.85) | 41.7 (30.95 - 54.37) | 0.30 (0.28-0.32) | 8.16 (4.77 - 12.50) | 8.96 (5.29 - 13.86) | 0.31 (0.29-0.33) |
| Saint Vincent and the Grenadines | 33.24 (24.77 - 43.26) | 40.46 (29.85 - 51.91) | 0.79 (0.76-0.82) | 7.10 (4.19 - 11.03) | 8.64 (5.06 - 13.35) | 0.78 (0.75-0.82) |
| Samoa | 26.71 (19.88 - 34.59) | 30.79 (22.95 - 39.85) | 0.67 (0.61-0.73) | 5.83 (3.47 - 8.92) | 6.73 (3.94 - 10.33) | 0.68 (0.62-0.74) |
| Sao Tome and Principe | 25.68 (19.17 - 33.20) | 27.78 (20.42 - 36.15) | 0.32 (0.22-0.43) | 5.44 (3.10 - 8.57) | 5.96 (3.44 - 9.03) | 0.35 (0.24-0.45) |
| Saudi Arabia | 41.17 (30.48 - 54.15) | 45.33 (33.73 - 58.83) | 0.35 (0.28-0.41) | 8.75 (5.02 - 13.50) | 9.69 (5.70 - 15.06) | 0.36 (0.31-0.42) |
| Senegal | 25.34 (18.70 - 33.20) | 26.07 (19.31 - 34.12) | 0.16 (0.10-0.22) | 5.33 (3.11 - 8.30) | 5.54 (3.23 - 8.74) | 0.19 (0.13-0.26) |
| Serbia | 31.90 (23.78 - 41.31) | 34.85 (26.05 - 45.08) | 0.61 (0.44-0.79) | 6.85 (4.02 - 10.48) | 7.51 (4.35 - 11.81) | 0.63 (0.45-0.81) |
| Seychelles | 38.68 (28.77 - 50.82) | 45.44 (34.00 - 59.70) | 0.43 (0.35-0.50) | 8.35 (4.88 - 12.63) | 9.81 (5.83 - 15.22) | 0.44 (0.36-0.52) |
| Sierra Leone | 23.72 (17.66 - 30.88) | 23.43 (17.26 - 30.33) | -0.18 (-0.33 to -0.03) | 5.01 (2.93 - 7.83) | 4.98 (2.84 - 7.78) | -0.14 (-0.30 to 0.02) |
| Singapore | 81.13 (59.37 - 107.56) | 122.38 (89.12 - 160.68) | 1.54 (1.50-1.57) | 17.36 (10.44 - 26.44) | 26.23 (15.68 - 40.47) | 1.55 (1.51-1.58) |
| Slovakia | 35.53 (26.47 - 45.81) | 43.13 (32.11 - 56.20) | 0.90 (0.78-1.01) | 7.62 (4.57 - 11.75) | 9.31 (5.45 - 14.22) | 0.93 (0.82-1.04) |
| Slovenia | 38.79 (28.83 - 50.07) | 43.50 (32.36 - 56.61) | 0.63 (0.56-0.70) | 8.32 (4.99 - 12.74) | 9.36 (5.57 - 14.34) | 0.63 (0.56-0.70) |
| Solomon Islands | 22.51 (16.61 - 29.35) | 24.00 (17.75 - 31.60) | 0.05 (-0.04 to 0.15) | 4.8 (2.8 - 7.570) | 5.18 (3.00 - 7.92) | 0.07 (-0.03 to 0.18) |
| Somalia | 17.75 (13.14 - 23.26) | 17.32 (12.85 - 22.71) | -0.09 (-0.17 to 0.01) | 3.75 (2.17 - 5.7) | 3.69 (2.11 - 5.67) | -0.07 (-0.16 to 0.03) |
| South Africa | 38.02 (28.59 - 48.90) | 40.62 (30.43 - 52.27) | 0.38 (0.31-0.45) | 8.10 (4.93 - 12.10) | 8.68 (5.22 - 13.04) | 0.39 (0.31-0.46) |
| South Korea | 65.46 (48.61 - 85.06) | 94.72 (71.20 - 123.31) | 1.37 (1.28-1.46) | 14.56 (8.97 – 22.00) | 21.84 (13.75 - 32.47) | 1.51 (1.40-1.62) |
| South Sudan | 27.31 (20.08 - 35.65) | 27.85 (20.90 - 36.35) | 0.19 (0.15-0.24) | 5.71 (3.27 - 8.93) | 5.89 (3.44 - 9.15) | 0.24 (0.19-0.29) |
| Spain | 140.68 (108.13 - 180.18) | 169.27 (124.80 - 222.36) | 0.66 (0.62-0.70) | 30.25 (19.11 - 45.61) | 36.22 (21.98 - 54.05) | 0.63 (0.59-0.67) |
| Sri Lanka | 26.97 (20.08 - 34.91) | 38.06 (28.12 - 49.89) | 1.25 (1.19-1.31) | 5.77 (3.39 - 8.73) | 8.19 (4.78 - 12.69) | 1.28 (1.23-1.33) |
| Sudan | 20.56 (15.38 - 26.83) | 25.11 (18.88 - 32.35) | 0.85 (0.75-0.94) | 4.34 (2.56 - 6.59) | 5.33 (3.13 - 8.06) | 0.85 (0.76-0.94) |
| Suriname | 38.53 (28.72 - 49.64) | 44.93 (33.28 - 58.02) | 0.72 (0.61-0.83) | 8.23 (4.80 - 12.49) | 9.58 (5.67 - 14.87) | 0.70 (0.59-0.81) |
| Swaziland | 33.27 (24.47 - 43.06) | 37.74 (27.93 - 49.31) | 0.40 (0.37-0.43) | 7.07 (4.13 - 10.90) | 7.98 (4.74 - 12.15) | 0.39 (0.36-0.42) |
| Sweden | 102.21 (75.99 - 132.71) | 116.85 (88.14 - 153.78) | 0.64 (0.59-0.68) | 22.57 (13.82 - 33.44) | 25.01 (15.12 - 38.33) | 0.55 (0.47-0.63) |
| Switzerland | 137.26 (103.75 - 177.52) | 135.02 (100.43 - 176.22) | -0.08 (-0.14 to -0.02) | 31.81 (20.30 - 46.84) | 30.92 (19.38 - 45.40) | -0.14 (-0.19 to -0.09) |
| Syria | 23.13 (17.36 - 29.97) | 26.67 (20.03 - 34.04) | 0.87 (0.74-1.01) | 4.90 (2.91 - 7.54) | 5.68 (3.24 - 8.85) | 0.90 (0.77-1.03) |
| Taiwan (Province of China) | 42.39 (31.29 - 55.15) | 57.56 (42.66 - 75.72) | 1.08 (0.99-1.17) | 9.36 (5.58 - 14.25) | 12.75 (7.76 - 19.44) | 1.09 (1.00-1.18) |
| Tajikistan | 27.10 (20.27 - 35.38) | 23.50 (17.30 - 30.35) | -0.44 (-0.80 to -0.08) | 5.85 (3.54 – 9.00) | 5.10 (2.95 - 8.03) | -0.42 (-0.79 to -0.06) |
| Tanzania | 23.19 (17.27 - 30.04) | 27.17 (20.18 - 35.46) | 0.68 (0.55-0.80) | 4.90 (2.90 - 7.56) | 5.80 (3.54 - 8.71) | 0.73 (0.60-0.86) |
| Thailand | 30.57 (22.66 - 39.78) | 41.22 (30.64 - 53.62) | 1.02 (0.98-1.07) | 6.60 (3.93 - 10.17) | 8.89 (5.15 - 13.66) | 1.03 (0.98-1.08) |
| The Bahamas | 51.07 (37.61 - 66.25) | 52.03 (38.26 - 67.61) | 0.21 (0.13-0.29) | 10.99 (6.69 - 16.69) | 11.17 (6.57 - 17.40) | 0.21 (0.13-0.28) |
| The Gambia | 24.00 (17.75 - 31.68) | 24.03 (17.94 - 31.13) | 0.06 (0.03-0.09) | 5.08 (2.85 - 7.84) | 5.09 (2.96 - 7.73) | 0.07 (0.04-0.10) |
| Timor-Leste | 20.65 (15.29 - 26.52) | 27.84 (20.80 - 36.21) | 1.27 (1.16-1.38) | 4.37 (2.61 - 6.67) | 5.97 (3.43 - 9.30) | 1.3 (1.19-1.41) |
| Togo | 23.21 (17.14 - 30.29) | 22.98 (17.05 - 29.97) | -0.08 (-0.13 to -0.03) | 4.90 (2.80 - 7.64) | 4.88 (2.87 - 7.56) | -0.04 (-0.10 to 0.01) |
| Tonga | 27.77 (20.78 - 35.86) | 30.76 (22.92 - 39.71) | 0.34 (0.31-0.36) | 5.98 (3.55 - 9.22) | 6.70 (3.97 - 10.33) | 0.39 (0.36-0.41) |
| Trinidad and Tobago | 43.67 (32.49 - 57.16) | 55.13 (40.93 - 71.79) | 1.29 (1.12-1.46) | 9.31 (5.52 - 14.31) | 11.79 (7.11 - 18.20) | 1.29 (1.12-1.46) |
| Tunisia | 26.36 (19.68 - 34.12) | 32.44 (24.32 - 41.87) | 0.84 (0.82-0.86) | 5.65 (3.25 - 8.59) | 6.93 (4.12 - 10.41) | 0.84 (0.82-0.87) |
| Turkey | 29.1 (21.59 - 37.82) | 35.78 (26.95 - 46.59) | 0.73 (0.64-0.82) | 6.20 (3.63 - 9.50) | 7.67 (4.60 - 11.75) | 0.75 (0.66-0.84) |
| Turkmenistan | 31.82 (23.32 - 40.74) | 37.35 (27.73 - 48.41) | 0.68 (0.36-1.01) | 6.90 (4.16 - 10.42) | 8.09 (4.89 - 12.61) | 0.67 (0.35-0.98) |
| Uganda | 20.19 (15.22 - 26.34) | 25.13 (18.49 - 32.65) | 0.87 (0.84-0.90) | 4.25 (2.44 - 6.56) | 5.34 (3.01 - 8.24) | 0.93 (0.89-0.97) |
| Ukraine | 33.64 (25.33 - 43.39) | 32.55 (23.97 - 42.27) | 0.06 (-0.23 to 0.35) | 7.20 (4.21 - 10.88) | 7.00 (4.10 - 10.79) | 0.07 (-0.22 to 0.36) |
| United Arab Emirates | 53.01 (38.65 - 70.17) | 45.09 (33.44 - 59.47) | -0.80 (-0.99 to -0.62) | 11.36 (6.67 - 17.41) | 9.68 (5.74 - 15.08) | -0.8 (-0.99 to -0.62) |
| United Kingdom | 103.82 (78.04 - 134.66) | 120.1 (90.86 - 154.95) | 0.64 (0.55-0.74) | 22.07 (13.59 - 32.88) | 26.04 (16.10 - 38.56) | 0.65 (0.53-0.78) |
| United States | 109.52 (82.62 - 141.17) | 118.84 (88.82 - 154.35) | 0.33 (0.25-0.41) | 23.48 (14.61 - 34.71) | 25.66 (16.02 - 38.30) | 0.36 (0.27-0.45) |
| Uruguay | 63.57 (46.80 - 81.48) | 79.2 (58.98 - 104.61) | 0.65 (0.53-0.77) | 13.65 (8.05 - 20.76) | 16.98 (10.15 - 25.88) | 0.63 (0.50-0.75) |
| Uzbekistan | 25.95 (19.47 - 33.92) | 29.67 (21.82 - 38.28) | 0.55 (0.37-0.72) | 5.58 (3.20 - 8.41) | 6.41 (3.73 - 9.87) | 0.55 (0.38-0.73) |
| Vanuatu | 25.28 (18.70 - 32.73) | 26.88 (20.11 - 34.83) | 0.21 (0.17-0.25) | 5.40 (3.10 - 8.18) | 5.83 (3.42 - 8.94) | 0.25 (0.20-0.29) |
| Venezuela | 42.25 (31.38 - 54.97) | 44.28 (32.77 - 57.55) | 0.24 (0.16-0.32) | 9.07 (5.33 - 13.91) | 9.54 (5.67 - 14.64) | 0.25 (0.17-0.33) |
| Vietnam | 22.03 (16.41 - 28.98) | 31.35 (23.31 - 40.73) | 1.36 (1.31-1.41) | 4.70 (2.81 - 7.41) | 6.79 (4.00 - 10.38) | 1.40 (1.35-1.45) |
| Virgin Islands, U.S. | 49.32 (36.72 - 64.34) | 66.78 (49.14 - 86.88) | 1.23 (1.02-1.44) | 10.62 (6.38 - 16.37) | 14.33 (8.35 - 22.02) | 1.23 (1.02-1.44) |
| Yemen | 21.55 (16.07 - 27.96) | 23.19 (17.23 - 30.47) | 0.55 (0.45-0.66) | 4.47 (2.57 - 6.79) | 4.87 (2.87 - 7.54) | 0.59 (0.48-0.69) |
| Zambia | 26.14 (19.54 - 33.80) | 29.57 (21.81 - 38.83) | 0.56 (0.37-0.75) | 5.55 (3.26 - 8.61) | 6.33 (3.66 - 9.78) | 0.61 (0.41-0.81) |
| Zimbabwe | 28.40 (21.27 - 36.65) | 25.51 (19.18 - 32.76) | -0.79 (-0.96 to -0.62) | 6.04 (3.55 - 9.29) | 5.43 (3.08 - 8.61) | -0.76 (-0.92 to -0.60) |

DALYs, disability-adjusted life-years; ASR, age-standardized rate; EAPC, estimated annual percentage change; UI, uncertainty interval.
