# Supplementary material for: Trends in the prevalence and disability-adjusted life years of eating disorders from 1990 to 2017: results from the Global Burden of Disease Study 2017
Source: Epidemiol Psychiatr Sci. 2020 Dec 7;29:e191. doi: 10.1017/S2045796020001055 (PMC7737181; doi:10.1017/S2045796020001055)
Supplement: Supplementary file 1 [file S2045796020001055sup001.zip › Supplementary_Table_3.docx]

**Supplementary Table 3. Age-standardized rates of prevalence and disability-adjusted life-years of eating disorders in 2017 and their temporal trend from 1990 to 2017 at national level.**

|  | **Prevalence (95% UI)** | | | **DALYs (95% UI)** | | |
| --- | --- | --- | --- | --- | --- | --- |
|  | **ASR in 1990**  **(per 100 000 population)** | **ASR in 2017**  **(per 100 000 population)** | **EAPC (%)** | **ASR in 2017**  **(per 100 000 population)** | **ASR in 1990**  **(per 100 000 population)** | **EAPC (%)** |
| Afghanistan | 101.05 (77.95 - 127.75) | 104.72 (80.86 - 133.18) | 0.35 (0.06-0.65) | 20.75 (13.00 - 30.43) | 21.82 (13.51 - 31.79) | 0.41 (0.12-0.70) |
| Albania | 126.23 (99.20 - 159.14) | 165.89 (128.84 - 207.95) | 1.34 (1.19-1.48) | 26.99 (16.96 - 40.46) | 35.46 (22.14 - 52.38) | 1.33 (1.19-1.48) |
| Algeria | 181.41 (140.79 - 228.66) | 201.02 (158.58 - 255.71) | 0.55 (0.45-0.65) | 38.31 (23.84 - 56.64) | 42.63 (26.85 - 62.83) | 0.57 (0.47-0.67) |
| American Samoa | 181.30 (143.67 - 224.52) | 170.64 (134.97 - 212.56) | -0.16 (-0.20 to -0.12) | 38.69 (23.99 - 56.49) | 36.51 (23.22 - 53.47) | -0.15 (-0.19 to -0.11) |
| Andorra | 538.34 (439.08 - 671.49) | 590.74 (472.82 - 729.96) | 0.51 (0.44-0.59) | 114.46 (74.63 - 167.07) | 125.96 (81.86 - 184.93) | 0.52 (0.44-0.60) |
| Angola | 137.31 (106.98 - 171.81) | 170.91 (134.49 - 215.33) | 1.02 (0.78-1.26) | 28.77 (18.23 - 41.95) | 36.02 (22.37 - 53.23) | 1.04 (0.80-1.28) |
| Antigua and Barbuda | 289.59 (227.33 - 364.25) | 335.74 (267.15 - 425.50) | 0.56 (0.50-0.61) | 61.27 (38.64 - 91.25) | 71.02 (45.85 - 104.9) | 0.56 (0.50-0.61) |
| Argentina | 303.08 (242.86 - 376.36) | 371.43 (298.28 - 465.87) | 0.68 (0.61-0.74) | 64.50 (41.55 - 94.43) | 78.92 (49.77 - 117.13) | 0.66 (0.59-0.73) |
| Armenia | 135.44 (104.67 - 170.76) | 158.77 (123.19 - 199.17) | 1.06 (0.72-1.39) | 29.12 (18.17 - 42.96) | 34.10 (21.71 - 51.05) | 1.04 (0.71-1.37) |
| Australia | 632.11 (502.53 - 793.75) | 843.81 (690.21 - 1025.90) | 1.32 (1.20-1.44) | 134.11 (86.56 - 196.57) | 178.58 (118.13 - 255.58) | 1.31 (1.18-1.43) |
| Austria | 495.53 (398.52 - 610.56) | 619.45 (499.47 - 769.74) | 0.90 (0.82-0.98) | 105.09 (67.44 - 152.93) | 133.72 (87.33 - 194.41) | 0.98 (0.90-1.07) |
| Azerbaijan | 160.79 (126.67 - 201.60) | 194.66 (152.78 - 245.58) | 1.10 (0.57-1.63) | 34.53 (21.66 - 50.51) | 41.75 (26.31 - 61.68) | 1.10 (0.57-1.63) |
| Bahrain | 231.32 (182.92 - 288.63) | 261.90 (207.14 - 327.74) | 0.41 (0.37-0.44) | 48.80 (30.77 – 72.00) | 55.36 (34.84 - 82.36) | 0.42 (0.39-0.46) |
| Bangladesh | 100.37 (76.69 - 125.93) | 135.15 (105.20 - 169.73) | 1.09 (0.98-1.20) | 21.33 (13.26 - 31.24) | 28.79 (18.02 - 42.27) | 1.10 (0.99-1.21) |
| Barbados | 290.92 (230.85 - 369.52) | 303.36 (240.17 - 385.15) | 0.22 (0.17-0.28) | 62.06 (39.53 - 91.77) | 64.64 (41.06 - 95.15) | 0.22 (0.16-0.27) |
| Belarus | 164.51 (130.09 - 207.93) | 196.64 (154.61 - 246.77) | 0.92 (0.66-1.19) | 34.95 (22.27 - 51.78) | 41.87 (26.27 - 61.52) | 0.92 (0.66-1.18) |
| Belgium | 463.92 (373.85 - 572.64) | 549.01 (443.95 - 681.20) | 0.63 (0.61-0.65) | 98.69 (62.85 - 144.92) | 116.82 (76.09 - 169.08) | 0.65 (0.63-0.67) |
| Belize | 205.23 (158.60 - 263.09) | 248.28 (195.05 - 317.19) | 0.64 (0.58-0.71) | 43.62 (27.51 - 64.76) | 52.82 (33.09 - 78.16) | 0.64 (0.58-0.71) |
| Benin | 109.71 (84.86 - 138.62) | 118.64 (93.33 - 150.52) | 0.37 (0.31-0.42) | 22.93 (14.51 - 34.50) | 25.17 (15.81 - 37.53) | 0.42 (0.36-0.47) |
| Bermuda | 402.22 (317.48 - 509.24) | 462.40 (368.57 - 587.88) | 0.69 (0.62-0.76) | 85.78 (53.87 - 127.01) | 98.56 (62.49 - 147.13) | 0.69 (0.62-0.76) |
| Bhutan | 114.77 (88.09 - 144.32) | 166.75 (130.09 - 210.82) | 1.45 (1.42-1.48) | 24.28 (15.21 - 35.98) | 35.50 (22.27 - 52.75) | 1.47 (1.44-1.50) |
| Bolivia | 239.63 (186.05 - 304.11) | 295.17 (229.49 - 379.50) | 0.75 (0.69-0.81) | 50.71 (31.96 - 75.9) | 62.77 (39.35 - 93.44) | 0.77 (0.71-0.83) |
| Bosnia and Herzegovina | 96.84 (74.67 - 123.50) | 164.11 (129.16 - 207.55) | 2.42 (2.15-2.70) | 20.77 (13.14 - 30.10) | 35.00 (22.19 - 51.08) | 2.40 (2.13-2.67) |
| Botswana | 169.54 (131.79 - 214.35) | 219.58 (172.73 - 276.20) | 0.93 (0.91-0.95) | 35.69 (22.75 - 52.21) | 46.08 (29.38 - 68.47) | 0.93 (0.92-0.94) |
| Brazil | 225.84 (178.76 - 283.86) | 265.13 (211.00 - 331.82) | 0.62 (0.58-0.66) | 47.76 (30.68 - 70.01) | 56.26 (36.06 - 82.09) | 0.63 (0.59-0.67) |
| Brunei | 507.88 (411.74 - 630.82) | 488.82 (391.70 - 612.58) | -0.03 (-0.06 to 0.01) | 108.97 (70.21 - 157.52) | 106.95 (69.47 - 154.83) | 0.05 (0.01-0.09) |
| Bulgaria | 168.32 (131.78 - 212.35) | 192.12 (151.75 - 240.58) | 0.64 (0.44-0.84) | 35.92 (22.40 - 53.21) | 41.16 (25.72 - 60.4) | 0.65 (0.46-0.85) |
| Burkina Faso | 94.58 (73.83 - 118.9) | 112.32 (87.08 - 141.64) | 0.68 (0.63-0.73) | 19.82 (12.57 - 28.96) | 23.87 (15.09 – 36.00) | 0.73 (0.68-0.79) |
| Burundi | 94.79 (74.00 - 119.24) | 88.94 (68.71 - 113.81) | -0.33 (-0.44 to -0.21) | 20.2 (12.63 - 29.90) | 19.01 (11.95 - 28.21) | -0.32 (-0.44 to -0.19) |
| Cambodia | 76.45 (58.38 - 96.01) | 106.22 (83.44 - 133.59) | 1.33 (1.21-1.44) | 16.27 (10.08 - 24.46) | 22.75 (14.34 - 33.81) | 1.35 (1.24-1.47) |
| Cameroon | 134.08 (103.89 - 168.32) | 131.32 (101.22 - 164.61) | 0.02 (-0.08 to 0.13) | 28.08 (18.03 - 41.11) | 27.87 (17.41 - 40.94) | 0.07 (-0.04 to 0.18) |
| Canada | 403.81 (325.95 - 500.21) | 433.59 (345.97 - 538.84) | 0.33 (0.30-0.36) | 85.81 (55.80 - 125.57) | 92.48 (59.59 - 135.61) | 0.34 (0.31-0.37) |
| Cape Verde | 119.93 (93.01 - 152.24) | 161.09 (126.87 - 202.52) | 1.29 (1.23-1.35) | 25.47 (15.97 - 37.71) | 34.38 (22.00 - 50.51) | 1.31 (1.24-1.37) |
| Central African Republic | 97.07 (75.41 - 121.92) | 88.35 (67.54 - 112.63) | -0.26 (-0.29 to -0.22) | 20.34 (12.98 - 29.61) | 18.74 (11.81 - 27.73) | -0.21 (-0.25 to -0.17) |
| Chad | 102.34 (78.34 - 128.91) | 120.63 (93.55 - 151.29) | 0.69 (0.58-0.80) | 21.51 (13.56 - 31.52) | 25.43 (16.10 - 37.55) | 0.70 (0.59-0.81) |
| Chile | 283.30 (225.75 - 354.29) | 381.41 (303.82 - 471.48) | 1.07 (1.05-1.09) | 60.04 (38.19 - 87.3) | 80.59 (51.27 - 119.24) | 1.06 (1.04-1.08) |
| China | 82.71 (64.55 - 102.01) | 150.20 (118.78 - 186.03) | 2.32 (2.23-2.42) | 17.90 (11.50 - 26.25) | 32.43 (20.52 - 48.03) | 2.31 (2.22-2.40) |
| Colombia | 203.51 (160.92 - 257.36) | 245.58 (194.25 - 309.23) | 0.63 (0.54-0.72) | 43.54 (27.01 - 65.12) | 52.55 (33.15 - 77.63) | 0.64 (0.55-0.73) |
| Comoros | 114.85 (88.68 - 144.38) | 107.60 (82.40 - 135.93) | -0.21 (-0.26 to -0.17) | 24.32 (15.19 - 35.93) | 23.01 (14.34 - 34.12) | -0.18 (-0.22 to -0.13) |
| Congo | 146.98 (113.80 - 186.58) | 158.90 (121.67 - 201.18) | 0.35 (0.24-0.47) | 30.80 (19.19 - 45.71) | 33.63 (21.44 - 49.40) | 0.39 (0.26-0.51) |
| Costa Rica | 222.49 (173.46 - 281.16) | 272.05 (216.19 - 342.04) | 0.75 (0.72-0.79) | 47.56 (30.03 - 71.26) | 58.13 (37.32 - 86.64) | 0.75 (0.71-0.79) |
| Cote d'Ivoire | 132.73 (103.04 - 166.23) | 133.89 (104.16 - 168.32) | -0.06 (-0.15 to 0.04) | 27.87 (17.33 - 40.63) | 28.42 (17.97 - 41.73) | -0.02 (-0.12 to 0.07) |
| Croatia | 183.11 (143.24 - 233.33) | 204.80 (159.39 - 256.09) | 0.72 (0.60-0.85) | 39.08 (24.93 - 57.58) | 43.95 (28.28 - 64.58) | 0.75 (0.63-0.88) |
| Cuba | 224.55 (175.31 - 282.44) | 248.57 (194.65 - 314.47) | 0.64 (0.42-0.86) | 47.96 (30.30 - 70.88) | 53.17 (33.42 - 78.55) | 0.64 (0.43-0.86) |
| Cyprus | 392.02 (315.64 - 483.76) | 468.99 (380.55 - 579.54) | 0.79 (0.71-0.88) | 82.93 (52.79 - 121.96) | 99.41 (64.23 - 148.44) | 0.80 (0.72-0.88) |
| Czech Republic | 198.69 (154.13 - 249.04) | 227.36 (178.85 - 282.14) | 0.65 (0.58-0.73) | 42.36 (26.79 - 62.38) | 48.40 (30.04 - 70.93) | 0.65 (0.58-0.71) |
| Democratic Republic of the Congo | 107.85 (82.83 - 134.85) | 91.64 (70.45 - 116.45) | -0.8 (-1.13 to -0.47) | 22.51 (14.18 - 32.92) | 19.43 (12.29 - 28.9) | -0.74 (-1.08 to -0.41) |
| Denmark | 446.06 (361.30 - 550.79) | 497.63 (402.26 - 617.99) | 0.51 (0.46-0.57) | 94.71 (61.23 - 137.77) | 105.58 (67.98 - 154.67) | 0.50 (0.44-0.56) |
| Djibouti | 133.77 (103.71 - 169.40) | 131.63 (102.18 - 165.19) | -0.05 (-0.22 to 0.12) | 28.60 (17.86 - 42.52) | 28.19 (17.78 - 42.06) | -0.04 (-0.21 to 0.13) |
| Dominica | 224.91 (173.77 - 286.36) | 264.92 (207.94 - 334.03) | 0.64 (0.62 to 0.66) | 47.90 (30.02 - 70.64) | 56.34 (35.26 - 82.62) | 0.63 (0.61-0.65) |
| Dominican Republic | 224.84 (177.14 - 281.05) | 290.64 (227.38 - 370.09) | 1.00 (0.96-1.03) | 48.13 (30.35 - 71.53) | 62.39 (39.54 - 93.63) | 1.00 (0.96-1.04) |
| Ecuador | 294.99 (227.48 - 378.40) | 347.27 (272.80 - 442.99) | 0.62 (0.53-0.71) | 62.79 (39.43 - 94.06) | 74.04 (47.05 - 111.89) | 0.62 (0.54-0.71) |
| Egypt | 143.17 (109.99 - 179.57) | 185.76 (145.34 - 233.19) | 1.02 (1.00-1.05) | 30.17 (19.03 - 44.55) | 39.29 (24.43 - 58.5) | 1.03 (1.01-1.06) |
| El Salvador | 179.34 (139.7 - 227.45) | 231.86 (184.80 - 295.87) | 0.99 (0.97-1.01) | 38.32 (24.22 - 57.38) | 49.72 (30.93 - 73.71) | 1.00 (0.98-1.02) |
| Equatorial Guinea | 94.83 (72.70 - 119.37) | 271.01 (214.59 - 340.25) | 5.18 (4.49-5.88) | 19.9 (12.26 - 29.46) | 57.10 (35.96 - 83.71) | 5.19 (4.50-5.89) |
| Eritrea | 86.20 (65.20 - 108.56) | 100.22 (77.38 – 126.00) | 0.38 (0.17-0.59) | 17.97 (11.23 - 26.71) | 21.36 (13.43 - 32.13) | 0.47 (0.27-0.68) |
| Estonia | 203.72 (162.79 - 255.83) | 231.40 (183.56 - 285.76) | 0.72 (0.62-0.82) | 47.39 (31.45 - 67.63) | 52.49 (34.34 - 75.27) | 0.54 (0.47-0.60) |
| Ethiopia | 83.19 (65.34 - 104.45) | 107.49 (83.09 - 133.92) | 1.01 (0.73-1.30) | 17.57 (11.38 - 25.71) | 22.93 (14.44 - 33.93) | 1.07 (0.79-1.35) |
| Federated States of Micronesia | 98.48 (76.51 - 123.68) | 106.58 (83.54 - 132.97) | 0.27 (0.24-0.30) | 21.09 (13.38 - 30.99) | 22.92 (14.41 - 33.85) | 0.28 (0.25-0.31) |
| Fiji | 124.24 (97.80 - 154.15) | 142.50 (111.12 - 177.71) | 0.46 (0.44-0.48) | 26.58 (17.07 - 39.14) | 30.56 (18.97 – 45.00) | 0.47 (0.45-0.48) |
| Finland | 487.54 (396.13 - 594.21) | 546.26 (445.83 - 675.10) | 0.53 (0.44-0.61) | 103.73 (66.03 - 148.91) | 116.52 (74.94 - 171.06) | 0.54 (0.45-0.63) |
| France | 455.81 (366.35 - 562.29) | 518.46 (416.99 - 645.26) | 0.49 (0.48-0.51) | 97.92 (63.29 - 142.80) | 111.23 (71.62 - 163.68) | 0.49 (0.47-0.50) |
| Gabon | 214.60 (171.08 - 267.95) | 221.91 (175.46 - 277.07) | 0.10 (0.08-0.12) | 44.93 (28.35 - 66.39) | 46.73 (29.74 - 68.49) | 0.11 (0.09-0.14) |
| Georgia | 163.37 (127.92 - 205.77) | 162.25 (126.57 – 205.00) | 0.33 (-0.13 to 0.80) | 35.23 (22.22 - 51.44) | 34.97 (21.83 - 52.66) | 0.32 (-0.13 to 0.78) |
| Germany | 417.54 (342.91 - 507.87) | 469.96 (382.42 - 573.46) | 0.43 (0.41-0.45) | 89.68 (57.63 - 128.41) | 101.41 (66.09 - 145.17) | 0.44 (0.42-0.46) |
| Ghana | 116.10 (90.52 - 146.32) | 144.28 (112.17 - 182.05) | 0.74 (0.66-0.83) | 24.54 (15.45 - 36.35) | 30.71 (19.34 - 45.76) | 0.76 (0.68-0.85) |
| Greece | 462.46 (374.79 - 568.82) | 514.70 (416.74 - 634.64) | 0.59 (0.48-0.69) | 97.76 (62.54 - 143.40) | 109.65 (71.47 - 160.47) | 0.62 (0.51-0.73) |
| Greenland | 445.08 (363.53 - 552.78) | 494.89 (400.64 - 605.49) | 0.5 (0.44-0.57) | 93.8 (61.37 - 135.65) | 104.76 (67.51 - 151.52) | 0.52 (0.45-0.59) |
| Grenada | 218.57 (170.37 - 274.38) | 275.93 (216.77 - 352.60) | 0.97 (0.91-1.02) | 46.51 (29.15 - 68.51) | 58.75 (37.12 - 88.24) | 0.96 (0.91-1.01) |
| Guam | 225.21 (176.62 - 277.41) | 241.74 (192.69 - 298.58) | 0.31 (0.29-0.34) | 48.36 (30.36 - 71.61) | 51.87 (33.25 - 75.86) | 0.31 (0.29-0.34) |
| Guatemala | 195.66 (153.98 - 247.18) | 221.77 (175.20 - 279.95) | 0.47 (0.45-0.49) | 41.53 (26.26 - 60.92) | 47.43 (29.79 - 70.92) | 0.50 (0.48-0.52) |
| Guinea | 111.17 (84.32 - 141.24) | 111.08 (86.24 - 141.74) | -0.08 (-0.16 to -0.01) | 23.40 (14.64 - 34.50) | 23.57 (14.57 - 35.01) | -0.05 (-0.11 to 0.02) |
| Guinea-Bissau | 107.51 (82.78 - 137.28) | 109.39 (84.08 - 138.28) | -0.04 (-0.08 to 0.01) | 22.68 (14.37 - 33.36) | 23.16 (14.53 - 34.17) | -0.02 (-0.06-0.02) |
| Guyana | 180.12 (140.84 - 225.79) | 230.04 (178.04 - 290.23) | 0.88 (0.83-0.92) | 38.06 (23.81 - 56.55) | 48.72 (30.89 - 72.68) | 0.89 (0.84 to 0.94) |
| Haiti | 161.95 (125.70 - 206.32) | 157.96 (123.11 - 200.92) | -0.03 (-0.08 to 0.01) | 34.04 (21.41 - 50.29) | 33.26 (20.99 - 48.75) | -0.02 (-0.07 to 0.02) |
| Honduras | 173.78 (133.87 - 220.81) | 195.75 (152.43 - 248.04) | 0.47 (0.42-0.52) | 37.17 (23.21 - 54.52) | 41.95 (26.43 - 63.37) | 0.48 (0.43-0.53) |
| Hungary | 190.05 (149.73 - 238.47) | 223.77 (177.03 - 279.99) | 0.76 (0.68-0.83) | 40.40 (25.82 - 59.59) | 47.68 (30.09 - 70.62) | 0.77 (0.69-0.85) |
| Iceland | 451.45 (361.79 - 554.79) | 515.22 (414.37 - 631.78) | 0.60 (0.56-0.63) | 96.05 (62.22 - 141.44) | 109.17 (70.76 - 159.45) | 0.59 (0.55-0.63) |
| India | 103.90 (81.72 - 128.79) | 154.81 (121.73 - 192.29) | 1.54 (1.44-1.63) | 21.99 (13.94 - 32.28) | 32.85 (20.91 - 47.94) | 1.55 (1.45-1.64) |
| Indonesia | 105.44 (83.15 - 129.93) | 144.44 (114.22 - 179.05) | 1.00 (0.91-1.09) | 22.57 (14.27 - 33.01) | 30.90 (19.56 - 45.42) | 1.00 (0.91-1.08) |
| Iran | 173.43 (136.67 - 215.9) | 220.96 (175.92 - 274.27) | 1.07 (1.00-1.14) | 36.61 (23.27 - 53.31) | 46.99 (30.14 - 68.21) | 1.10 (1.03-1.17) |
| Iraq | 174.73 (136.05 - 219.37) | 200.76 (156.55 - 251.36) | 0.83 (0.63-1.02) | 36.46 (23.11 - 53.34) | 42.30 (26.40 - 62.01) | 0.87 (0.67-1.06) |
| Ireland | 358.41 (286.59 - 446.20) | 511.61 (409.63 - 633.50) | 1.43 (1.34-1.53) | 75.73 (48.69 – 112.00) | 108.2 (69.83 - 158.71) | 1.43 (1.34-1.52) |
| Israel | 345.39 (276.89 - 433.55) | 428.79 (343.16 - 531.93) | 0.82 (0.78-0.86) | 73.08 (46.85 - 107.70) | 90.91 (57.87 - 133.46) | 0.83 (0.78-0.87) |
| Italy | 546.47 (440.66 - 675.33) | 578.80 (470.34 - 713.03) | 0.16 (0.08-0.24) | 117.09 (75.43 - 171.94) | 123.23 (79.98 - 178.54) | 0.13 (0.05-0.21) |
| Jamaica | 230.11 (180.58 - 291.99) | 249.11 (197.42 - 317.02) | 0.26 (0.24-0.29) | 49.06 (30.69 - 73.27) | 53.06 (34.11 – 79.00) | 0.25 (0.23-0.28) |
| Japan | 369.83 (300.80 - 456.60) | 408.67 (331.62 - 503.82) | 0.31 (0.23-0.39) | 80.04 (51.57 - 114.96) | 89.20 (58.36 - 129.10) | 0.32 (0.22-0.42) |
| Jordan | 159.45 (125.26 - 199.63) | 180.63 (141.05 - 227.21) | 0.68 (0.57-0.80) | 33.69 (21.26 - 49.82) | 38.34 (24.01 - 56.63) | 0.69 (0.58-0.81) |
| Kazakhstan | 180.75 (141.47 - 226.93) | 216.30 (171.76 - 274.53) | 0.91 (0.62-1.20) | 38.57 (24.74 - 56.73) | 46.18 (28.64 - 68.98) | 0.91 (0.62-1.19) |
| Kenya | 121.21 (94.72 - 150.99) | 131.55 (103.21 - 163.28) | 0.25 (0.16-0.33) | 25.85 (16.53 - 37.82) | 28.07 (17.76 - 41.28) | 0.26 (0.17-0.34) |
| Kiribati | 93.46 (72.45 - 117.51) | 92.07 (71.59 - 114.25) | -0.06 (-0.09 to -0.04) | 19.92 (12.52 - 28.88) | 19.65 (12.44 - 28.74) | -0.06 (-0.09 to -0.03) |
| Kuwait | 269.22 (213.84 - 341.62) | 323.99 (256.81 - 406.77) | 0.75 (0.70-0.80) | 57.29 (36.03 - 83.91) | 69.12 (43.21 - 103.32) | 0.75 (0.70-0.80) |
| Kyrgyzstan | 139.31 (109.06 - 174.88) | 122.60 (95.39 - 153.48) | -0.44 (-0.72 to -0.15) | 30.18 (19.26 - 44.21) | 26.67 (16.65 - 39.34) | -0.44 (-0.71 to -0.16) |
| Laos | 86.13 (65.72 - 108.43) | 124.05 (97.49 - 154.67) | 1.35 (1.24-1.46) | 18.34 (11.49 - 27.31) | 26.63 (16.61 - 39.36) | 1.38 (1.27-1.49) |
| Latvia | 187.54 (147.57 - 236.94) | 210.17 (165.6 - 262.92) | 0.79 (0.54-1.04) | 39.92 (25.42 - 58.65) | 44.77 (27.66 - 65.75) | 0.79 (0.54-1.04) |
| Lebanon | 180.98 (141.40 - 229.46) | 207.53 (161.53 - 261.10) | 0.71 (0.64-0.79) | 38.14 (24.52 - 56.36) | 43.98 (27.79 - 65.09) | 0.73 (0.66-0.80) |
| Lesotho | 105.86 (81.46 - 134.13) | 133.35 (102.48 - 167.97) | 0.83 (0.81-0.85) | 22.45 (14.28 - 33.05) | 28.02 (17.70 - 40.91) | 0.80 (0.77-0.83) |
| Liberia | 97.67 (74.75 - 122.12) | 90.64 (68.46 - 114.81) | 0.14 (-0.15 to 0.43) | 20.55 (12.82 - 29.90) | 19.05 (11.99 - 28.3) | 0.14 (-0.16 to 0.45) |
| Libya | 231.90 (183.38 - 293.78) | 186.54 (146.36 - 233.65) | -0.28 (-0.49 to -0.07) | 48.96 (30.60 - 71.86) | 39.51 (24.85 - 57.41) | -0.28 (-0.49 to -0.07) |
| Lithuania | 183.57 (145.17 - 227.51) | 218.97 (171.85 - 275.71) | 0.95 (0.72-1.18) | 38.93 (24.40 - 57.43) | 46.38 (29.17 - 68.81) | 0.94 (0.71-1.18) |
| Luxembourg | 543.59 (440.44 - 668.59) | 678.36 (547.25 - 828.14) | 0.84 (0.74-0.93) | 117.47 (75.79 - 171.37) | 146.67 (95.4 - 214.23) | 0.84 (0.75-0.92) |
| Macedonia | 159.22 (124.56 - 201.17) | 174.92 (137.37 - 220.71) | 0.48 (0.33-0.63) | 33.98 (21.24 - 50.27) | 37.44 (23.68 - 54.89) | 0.49 (0.34-0.63) |
| Madagascar | 107.40 (80.99 - 135.50) | 104.91 (80.69 - 132.01) | -0.1 (-0.17 to -0.03) | 22.73 (14.18 - 33.72) | 22.39 (14.04 - 33.24) | -0.07 (-0.14 to -0.01) |
| Malawi | 93.47 (72.43 - 118.36) | 98.21 (75.58 - 124.67) | 0.27 (0.21-0.34) | 19.82 (12.46 - 28.94) | 20.98 (13.14 - 30.60) | 0.32 (0.24-0.40) |
| Malaysia | 140.03 (110.23 - 174.92) | 188.3 (148.86 - 233.82) | 1.04 (1.00-1.09) | 30.01 (18.91 - 44.04) | 40.33 (24.95 - 59.20) | 1.04 (1.00-1.08) |
| Maldives | 113.63 (88.96 - 141.62) | 150.53 (118.45 - 186.80) | 1.18 (1.08-1.29) | 24.17 (15.2 - 35.50) | 32.24 (20.54 – 48.00) | 1.21 (1.10-1.32) |
| Mali | 98.24 (76.72 - 122.17) | 115.32 (89.56 - 144.70) | 0.65 (0.63-0.68) | 20.62 (12.62 - 30.43) | 24.39 (15.87 - 36.23) | 0.68 (0.65-0.70) |
| Malta | 363.59 (295.55 - 452.23) | 476.84 (383.38 - 588.20) | 0.98 (0.92-1.04) | 77.67 (49.98 - 113.16) | 101.70 (65.08 - 148.52) | 0.97 (0.92-1.03) |
| Marshall Islands | 99.89 (77.25 - 124.44) | 109.22 (85.63 - 136.50) | 0.27 (0.23-0.31) | 21.48 (13.58 - 31.68) | 23.61 (15.24 - 34.82) | 0.29 (0.26-0.33) |
| Mauritania | 127.15 (99.27 - 159.08) | 141.82 (111.28 - 179.07) | 0.43 (0.34-0.52) | 26.93 (16.86 - 39.56) | 30.23 (19.37 - 44.51) | 0.45 (0.37-0.54) |
| Mauritius | 130.30 (102.15 - 163.12) | 176.71 (139.69 - 219.04) | 1.05 (1.01-1.09) | 27.81 (17.52 - 41.48) | 37.54 (23.62 - 54.97) | 1.03 (0.99-1.08) |
| Mexico | 279.13 (221.56 - 348.82) | 298.19 (237.48 - 372.70) | 0.25 (0.23-0.26) | 59.68 (38.53 - 87.95) | 63.82 (40.41 - 94.58) | 0.24 (0.23-0.26) |
| Moldova | 148.98 (116.74 - 186.92) | 134.53 (104.75 - 169.75) | -0.27 (-0.63 to 0.09) | 32.55 (20.95 - 47.43) | 29.75 (19.32 - 43.44) | -0.25 (-0.58 to 0.09) |
| Mongolia | 132.95 (103.61 - 167.08) | 172.25 (133.28 - 216.28) | 1.06 (0.86-1.26) | 28.51 (18.3 - 42.04) | 36.86 (23.01 - 54.90) | 1.04 (0.84-1.24) |
| Montenegro | 168.07 (132.65 - 212.37) | 183.5 (144.59 - 231.36) | 0.61 (0.43-0.79) | 35.88 (22.58 - 53.52) | 39.25 (24.78 - 58.77) | 0.61 (0.43-0.78) |
| Morocco | 136.12 (106.95 - 172.77) | 168.34 (131.30 - 213.07) | 0.77 (0.72-0.82) | 28.63 (17.88 - 42.72) | 35.51 (22.57 – 53.00) | 0.78 (0.73-0.82) |
| Mozambique | 76.56 (57.78 - 97.98) | 101.48 (78.00 - 127.9) | 1.25 (1.16-1.35) | 16.19 (10.01 - 23.78) | 21.50 (13.45 - 31.64) | 1.26 (1.17-1.35) |
| Myanmar | 73.52 (56.31 - 93.64) | 122.27 (95.20 - 153.58) | 2.14 (1.94-2.33) | 15.72 (9.86 - 23.39) | 26.21 (16.51 - 38.81) | 2.14 (1.94-2.33) |
| Namibia | 166.13 (130.39 - 210.49) | 194.24 (153.58 - 244.43) | 0.64 (0.53-0.74) | 35.07 (22.14 - 52.24) | 41.07 (26.20 - 61.19) | 0.63 (0.53-0.74) |
| Nepal | 97.29 (74.56 - 122.10) | 125.18 (97.66 - 158.37) | 0.91 (0.85-0.96) | 20.49 (13.08 - 30.27) | 26.64 (16.69 - 39.65) | 0.94 (0.89-1.00) |
| Netherlands | 315.08 (260.07 - 384.96) | 426.62 (347.71 - 522.07) | 1.30 (1.22-1.38) | 66.9 (43.74 - 96.02) | 92.73 (61.16 - 134.74) | 1.43 (1.35-1.51) |
| New Zealand | 564.69 (455.10 - 700.61) | 598.3 (477.38 - 741.75) | 0.31 (0.27-0.35) | 118.84 (76.32 - 174.92) | 126.09 (81.86 - 185.56) | 0.32 (0.27-0.37) |
| Nicaragua | 181.94 (142.37 - 232.44) | 194.77 (152.46 - 247.55) | 0.34 (0.25-0.44) | 38.63 (24.05 - 56.42) | 41.79 (26.14 - 62.94) | 0.39 (0.29-0.48) |
| Niger | 95.28 (73.28 - 122.59) | 94.96 (72.59 - 120.68) | -0.01 (-0.12 to 0.09) | 20.22 (12.59 - 30.04) | 20.29 (12.47 - 30.34) | 0.01 (-0.10 to 0.12) |
| Nigeria | 127.31 (98.64 - 158.91) | 160.81 (124.74 - 202.49) | 1.23 (0.97-1.50) | 26.84 (16.77 - 39.43) | 34.02 (21.59 - 50.03) | 1.25 (0.98-1.52) |
| North Korea | 113.43 (88.79 - 140.71) | 87.12 (68.74 - 109.30) | -1.12 (-1.32 to -0.91) | 24.45 (15.33 - 35.96) | 18.83 (11.96 - 27.89) | -1.11 (-1.32 to -0.9) |
| Northern Mariana Islands | 241.05 (192.17 - 300.7) | 187.13 (148.31 - 235.54) | -0.93 (-1.00 to -0.85) | 51.75 (33.22 - 76.95) | 40.10 (25.59 - 59.82) | -0.93 (-1.01 to -0.85) |
| Norway | 488.16 (397.65 - 596.11) | 524.1 (424.89 - 644.82) | 0.27 (0.25-0.29) | 105.18 (68.80 - 152.17) | 111.19 (71.88 - 162.43) | 0.22 (0.20-0.23) |
| Oman | 208.67 (162.09 - 261.40) | 244.60 (192.50 - 306.45) | 0.69 (0.54-0.84) | 44.05 (27.55 - 64.99) | 51.93 (32.92 - 76.79) | 0.71 (0.56-0.86) |
| Pakistan | 124.04 (98.17 - 155.47) | 150.12 (116.97 - 187.26) | 0.73 (0.68-0.78) | 26.32 (16.28 - 39.21) | 32.02 (20.10 - 46.98) | 0.75 (0.70-0.80) |
| Palestine | 117.96 (92.12 - 149.16) | 137.55 (106.7 - 171.99) | 0.44 (0.37-0.51) | 24.85 (15.81 - 36.55) | 29.10 (18.35 - 43.16) | 0.45 (0.37-0.52) |
| Panama | 224.97 (177.94 - 284.54) | 299.50 (235.89 - 375.99) | 1.04 (0.96-1.12) | 48.46 (30.15 - 72.88) | 64.66 (41.56 - 96.93) | 1.05 (0.97-1.13) |
| Papua New Guinea | 91.17 (70.47 - 114.93) | 103.44 (79.23 - 129.13) | 0.29 (0.21-0.37) | 19.38 (12.04 - 28.65) | 22.01 (13.77 - 32.16) | 0.30 (0.22-0.37) |
| Paraguay | 197.29 (156.18 - 249.39) | 229.06 (179.63 - 287.05) | 0.48 (0.40-0.55) | 42.17 (26.74 - 61.87) | 49.19 (31.04 - 73.22) | 0.49 (0.42-0.57) |
| Peru | 285.67 (221.44 - 367.13) | 355.43 (276.72 - 457.60) | 0.89 (0.84-0.94) | 60.84 (38.08 - 91.24) | 75.94 (47.69 - 113.13) | 0.90 (0.85-0.95) |
| Philippines | 108.88 (85.35 - 136.14) | 130.93 (102.64 - 161.64) | 0.62 (0.55-0.70) | 23.26 (14.48 - 33.81) | 28.11 (17.53 - 41.39) | 0.64 (0.56-0.72) |
| Poland | 161.26 (126.12 - 202.36) | 215.19 (170.44 - 270.18) | 1.20 (1.14-1.25) | 34.55 (21.78 - 50.44) | 46.39 (29.65 - 68.49) | 1.23 (1.18-1.29) |
| Portugal | 378.30 (305.81 - 467.82) | 470.96 (375.74 - 576.27) | 0.84 (0.76-0.92) | 79.8 (51.76 - 116.39) | 99.53 (64.31 – 144.00) | 0.84 (0.76-0.92) |
| Puerto Rico | 338.13 (268.63 - 431.81) | 388.98 (307.63 - 491.14) | 0.57 (0.48-0.66) | 72.47 (46.37 - 108.22) | 83.34 (53.98 - 123.63) | 0.56 (0.47-0.66) |
| Qatar | 272.91 (215.89 - 342.45) | 300.04 (235.04 - 376.34) | 0.57 (0.42-0.71) | 57.69 (36.20 - 84.54) | 63.61 (40.12 - 94.40) | 0.58 (0.43-0.73) |
| Romania | 157.97 (124.71 - 198.83) | 191.23 (150.9 - 238.99) | 0.94 (0.75-1.13) | 33.54 (20.79 - 49.62) | 40.83 (25.39 - 60.64) | 0.96 (0.77-1.15) |
| Russian Federation | 207.62 (165.15 - 257.45) | 218.11 (171.60 - 271.15) | 0.34 (0.05-0.62) | 44.18 (28.32 - 64.53) | 46.35 (29.73 - 67.75) | 0.32 (0.03-0.60) |
| Rwanda | 97.47 (75.02 - 122.47) | 112.58 (87.93 - 142.72) | 0.68 (0.48-0.87) | 20.75 (13.22 - 30.45) | 23.94 (15.10 - 35.36) | 0.69 (0.49-0.90) |
| Saint Lucia | 245.98 (194.94 - 312.85) | 272.97 (214.43 - 344.83) | 0.34 (0.32-0.37) | 52.29 (32.35 - 77.29) | 58.08 (36.42 - 85.52) | 0.35 (0.32-0.37) |
| Saint Vincent and the Grenadines | 212.53 (166.03 - 269.28) | 264.09 (207.71 - 331.86) | 0.86 (0.83-0.89) | 45.16 (28.5 - 67.27) | 56.05 (35.29 - 82.78) | 0.86 (0.83-0.89) |
| Samoa | 106.71 (84.20 - 134.38) | 122.49 (97.30 - 152.87) | 0.68 (0.61-0.74) | 22.99 (14.41 - 33.65) | 26.41 (16.76 - 39.12) | 0.68 (0.61-0.74) |
| Sao Tome and Principe | 120.57 (93.90 - 153.28) | 131.69 (101.76 - 166.05) | 0.36 (0.25-0.46) | 25.63 (16.25 - 38.01) | 28.13 (17.78 - 41.47) | 0.38 (0.27-0.48) |
| Saudi Arabia | 244.05 (191.09 - 305.68) | 276.60 (218.24 - 351.81) | 0.47 (0.40-0.55) | 51.51 (32.73 - 76.55) | 58.61 (36.99 - 86.19) | 0.49 (0.41-0.56) |
| Senegal | 117.48 (91.00 - 147.80) | 123.36 (95.55 - 155.9) | 0.22 (0.16-0.29) | 24.73 (15.44 - 35.75) | 26.17 (16.61 - 38.71) | 0.25 (0.19-0.32) |
| Serbia | 160.87 (123.88 - 201.42) | 177.56 (136.89 - 225.24) | 0.69 (0.50-0.89) | 34.32 (21.71 - 50.92) | 37.96 (23.96 - 56.22) | 0.69 (0.50-0.88) |
| Seychelles | 154.3 (121.09 - 191.60) | 187.3 (148.02 - 233.42) | 0.56 (0.50-0.63) | 33.10 (20.96 - 48.80) | 40.14 (25.20 - 59.23) | 0.57 (0.50-0.64) |
| Sierra Leone | 109.12 (84.21 - 136.89) | 108.77 (84.18 - 137.43) | -0.15 (-0.34 to 0.03) | 22.97 (14.73 - 33.85) | 23.05 (14.48 - 34.34) | -0.12 (-0.31 to 0.06) |
| Singapore | 343.11 (273.30 - 424.29) | 498.79 (399.15 - 619.73) | 1.39 (1.37-1.40) | 73.1 (46.54 - 108.47) | 106.59 (68.52 - 155.59) | 1.40 (1.38-1.42) |
| Slovakia | 179.60 (142.35 - 224.88) | 221.68 (174.95 - 279.85) | 0.99 (0.86-1.11) | 38.31 (24.65 - 56.20) | 47.32 (29.93 - 70.59) | 0.99 (0.87-1.11) |
| Slovenia | 196.67 (155.17 - 243.6) | 223.79 (178.33 - 279.8) | 0.70 (0.62-0.78) | 41.9 (26.36 - 61.53) | 47.63 (30.66 - 69.94) | 0.69 (0.61-0.76) |
| Solomon Islands | 86.48 (65.64 - 109.31) | 92.71 (72.33 - 116.90) | 0.06 (-0.05 to 0.16) | 18.47 (11.47 - 27.32) | 19.80 (12.57 - 28.94) | 0.06 (-0.04 to 0.16) |
| Somalia | 81.81 (62.11 - 104.79) | 78.38 (59.77 - 99.32) | -0.15 (-0.25 to -0.05) | 17.35 (10.72 - 25.71) | 16.73 (10.66 - 24.56) | -0.12 (-0.22 to -0.02) |
| South Africa | 193.89 (153.11 - 240.92) | 207.13 (163.93 - 257.23) | 0.39 (0.32-0.46) | 41.06 (26.31 - 60.32) | 43.71 (27.58 - 64.24) | 0.36 (0.29-0.43) |
| South Korea | 266.70 (213.27 - 330.72) | 384.40 (309.09 - 478.58) | 1.30 (1.22-1.39) | 57.44 (37.08 - 83.21) | 83.62 (54.31 - 121.18) | 1.35 (1.25-1.44) |
| South Sudan | 131.93 (102.19 - 165.72) | 132.42 (103.55 - 167.40) | 0.15 (0.08-0.21) | 27.54 (17.13 - 41.18) | 27.87 (17.69 - 41.10) | 0.18 (0.12-0.25) |
| Spain | 524.53 (430.22 - 638.6) | 666.98 (533.25 - 832.80) | 0.99 (0.94-1.04) | 111.55 (71.96 - 163.20) | 141.88 (91.73 - 208.10) | 0.99 (0.93-1.04) |
| Sri Lanka | 104.16 (82.45 - 132.28) | 151.57 (117.53 - 188.78) | 1.35 (1.30-1.41) | 22.24 (14.25 - 32.81) | 32.55 (20.22 - 47.78) | 1.38 (1.32-1.43) |
| Sudan | 111.97 (86.32 - 140.82) | 143.34 (112.03 - 180.69) | 1.04 (0.95-1.14) | 23.51 (14.93 - 34.54) | 30.26 (18.54 - 45.06) | 1.06 (0.96-1.16) |
| Suriname | 251.53 (198.44 - 317.01) | 295.84 (233.22 - 377.35) | 0.77 (0.65-0.88) | 53.39 (33.77 - 78.96) | 62.8 (39.39 - 92.59) | 0.76 (0.64-0.88) |
| Swaziland | 160.05 (125.79 - 201.94) | 184.57 (145.92 - 234.03) | 0.48 (0.44-0.51) | 33.92 (20.96 - 50.30) | 38.67 (23.93 - 57.47) | 0.42 (0.40-0.45) |
| Sweden | 438.20 (350.99 - 546.81) | 529.73 (425.58 - 656.02) | 0.77 (0.73-0.81) | 93.59 (60.16 - 136.81) | 112.10 (70.95 - 162.68) | 0.74 (0.7-0.79) |
| Switzerland | 482.85 (394.24 - 590.66) | 523.87 (424.10 - 642.96) | 0.31 (0.28-0.33) | 105.15 (69.45 - 150.75) | 113.49 (74.63 - 165.10) | 0.28 (0.25-0.31) |
| Syria | 129.04 (100.71 - 163.09) | 152.60 (120.33 - 191.17) | 1.03 (0.88-1.19) | 27.30 (17.25 - 40.19) | 32.32 (20.61 - 48.22) | 1.04 (0.88-1.19) |
| Taiwan (Province of China) | 159.79 (124.83 - 199.24) | 222.39 (174.54 - 275.70) | 1.17 (1.08-1.27) | 34.53 (21.83 - 50.68) | 48.03 (30.41 - 70.08) | 1.17 (1.07-1.26) |
| Tajikistan | 133.83 (104.45 - 167.75) | 114.30 (87.39 - 144.46) | -0.47 (-0.88 to -0.06) | 28.66 (18.04 - 42.34) | 24.59 (15.39 – 36.00) | -0.46 (-0.87 to -0.05) |
| Tanzania | 106.77 (82.32 - 136.48) | 127.40 (97.97 - 161.34) | 0.77 (0.63-0.91) | 22.58 (13.94 - 33.78) | 27.11 (17.16 - 39.97) | 0.79 (0.65-0.94) |
| Thailand | 119.73 (93.43 - 149.96) | 166.00 (130.27 - 204.49) | 1.12 (1.07-1.17) | 25.74 (16.01 - 38.37) | 35.57 (22.40 - 52.34) | 1.11 (1.06-1.16) |
| The Bahamas | 337.10 (266.08 - 421.07) | 344.35 (271.27 - 435.14) | 0.21 (0.14-0.28) | 71.78 (45.12 - 106.48) | 73.27 (46.32 - 109.10) | 0.21 (0.14-0.29) |
| The Gambia | 111.24 (85.82 - 140.38) | 111.35 (86.64 - 141.42) | 0.07 (0.04-0.10) | 23.39 (14.66 - 34.26) | 23.50 (14.88 - 34.89) | 0.08 (0.05-0.11) |
| Timor-Leste | 77.88 (60.53 - 99.44) | 108.58 (84.12 - 136.94) | 1.38 (1.26-1.50) | 16.58 (10.40 - 24.24) | 23.24 (14.77 - 34.19) | 1.41 (1.28-1.54) |
| Togo | 108.09 (83.76 - 137.55) | 106.85 (82.52 - 136.78) | -0.08 (-0.14 to -0.01) | 22.76 (14.05 - 34.05) | 22.71 (14.16 - 33.27) | -0.05 (-0.11 to 0.02) |
| Tonga | 109.04 (84.49 - 137.84) | 121.50 (94.51 - 151.10) | 0.38 (0.36-0.40) | 23.36 (14.68 - 34.40) | 26.10 (16.68 - 38.6) | 0.39 (0.37-0.42) |
| Trinidad and Tobago | 285.53 (224.56 - 359.96) | 367.56 (288.88 - 466.49) | 1.36 (1.19-1.54) | 60.62 (38.49 - 89.67) | 77.94 (49.36 - 115.92) | 1.36 (1.19-1.53) |
| Tunisia | 148.94 (117.27 - 188.52) | 189.39 (146.11 - 236.45) | 0.98 (0.95-1.01) | 31.63 (19.74 - 46.67) | 40.23 (25.29 - 59.72) | 0.98 (0.95-1.01) |
| Turkey | 205.83 (165.37 - 256.33) | 290.55 (226.56 - 368.37) | 1.32 (1.24-1.39) | 43.38 (27.87 - 64.38) | 61.68 (39.23 - 90.91) | 1.34 (1.27-1.41) |
| Turkmenistan | 159.56 (125.00 - 199.53) | 191.29 (151.29 - 239.46) | 0.77 (0.41-1.13) | 34.23 (21.54 - 50.33) | 41.04 (26.12 - 60.30) | 0.76 (0.41-1.12) |
| Uganda | 91.43 (70.43 - 115.09) | 117.43 (90.28 - 146.52) | 1.00 (0.96-1.04) | 19.16 (12.04 - 28.36) | 24.93 (15.60 - 36.65) | 1.05 (1.00-1.09) |
| Ukraine | 169.03 (131.70 - 210.71) | 162.48 (125.88 - 205.96) | 0.07 (-0.25 to 0.40) | 35.88 (22.32 - 53.48) | 34.63 (22.19 - 51.61) | 0.09 (-0.23 to 0.40) |
| United Arab Emirates | 285.77 (225.46 - 357.06) | 248.82 (198.52 - 312.8) | -0.57 (-0.69 to -0.45) | 60.45 (37.61 - 88.96) | 52.76 (32.97 - 77.79) | -0.57 (-0.69 to -0.45) |
| United Kingdom | 417.64 (338.09 - 511.36) | 496.32 (402.05 - 608.30) | 0.72 (0.64-0.79) | 88.19 (57.28 - 127.46) | 105.62 (68.60 - 152.48) | 0.72 (0.64-0.81) |
| United States | 427.82 (344.48 - 527.35) | 466.60 (376.00 - 575.09) | 0.39 (0.28-0.50) | 90.44 (58.71 - 130.89) | 98.98 (64.01 - 144.99) | 0.40 (0.29-0.52) |
| Uruguay | 299.27 (238.27 - 376.16) | 371.51 (294.45 - 467.51) | 0.68 (0.58-0.78) | 63.72 (40.08 - 93.32) | 79.05 (50.82 - 117.39) | 0.67 (0.57-0.77) |
| Uzbekistan | 127.17 (99.09 - 160.33) | 147.63 (116.69 - 184.14) | 0.62 (0.41-0.82) | 27.15 (17.46 - 39.90) | 31.63 (20.16 - 46.25) | 0.62 (0.42-0.82) |
| Vanuatu | 97.89 (75.51 - 122.59) | 104.66 (81.20 - 131.13) | 0.23 (0.19-0.26) | 20.84 (13.13 - 30.89) | 22.38 (14.10 - 32.58) | 0.24 (0.21-0.27) |
| Venezuela | 262.33 (205.71 - 330.26) | 270.42 (215.75 - 342.77) | 0.17 (0.09-0.25) | 56.00 (35.06 - 82.90) | 57.82 (36.41 - 85.24) | 0.18 (0.10-0.26) |
| Vietnam | 82.74 (63.84 - 104.12) | 124.01 (96.95 - 154.88) | 1.54 (1.50-1.58) | 17.72 (11.12 - 26.28) | 26.75 (16.89 - 39.68) | 1.57 (1.53-1.61) |
| Virgin Islands, U.S. | 323.94 (257.03 - 412.62) | 444.87 (349.58 - 566.58) | 1.30 (1.10-1.50) | 69.01 (43.73 - 103.14) | 94.77 (59.68 - 140.75) | 1.30 (1.10-1.50) |
| Yemen | 118.56 (91.85 - 150.03) | 130.58 (103.25 - 163.38) | 0.65 (0.53-0.77) | 24.48 (15.4 - 36.33) | 27.19 (17.02 - 40.38) | 0.68 (0.57-0.80) |
| Zambia | 122.81 (95.95 - 154.50) | 140.26 (107.42 - 176.50) | 0.63 (0.41-0.84) | 26.03 (16.45 - 38.60) | 29.85 (18.64 - 44.74) | 0.65 (0.43-0.87) |
| Zimbabwe | 135.86 (105.83 - 171.44) | 120.62 (93.84 - 151.37) | -0.88 (-1.06 to -0.69) | 28.72 (18.11 - 42.17) | 25.62 (16.05 - 38.20) | -0.85 (-1.02 to -0.67) |

DALYs, disability-adjusted life-years; ASR, age-standardized rate; EAPC, estimated annual percentage change; UI, uncertainty interval.
