# Supplementary material for: Trends in the prevalence and disability-adjusted life years of eating disorders from 1990 to 2017: results from the Global Burden of Disease Study 2017
Source: Epidemiol Psychiatr Sci. 2020 Dec 7;29:e191. doi: 10.1017/S2045796020001055 (PMC7737181; doi:10.1017/S2045796020001055)
Supplement: Supplementary file 1 [file S2045796020001055sup001.zip › Supplementary_Table_2.docx]

**Supplementary Table 2. Age-standardized rates of prevalence and disability-adjusted life-years of bulimia nervosa in 2017 and their temporal trend from 1990 to 2017 at global and regional levels.**

|  | **Prevalence (95% UI)** | | | **DALYs (95% UI)** | | |
| --- | --- | --- | --- | --- | --- | --- |
|  | **ASR in 1990**  **(per 100 000 population)** | **ASR in 2017**  **(per 100 000 population)** | **EAPC (%)** | **ASR in 1990**  **(per 100 000 population)** | **ASR in 2017**  **(per 100 000 population)** | **EAPC (%)** |
| **Global** | 134.19 (101.98 - 171.29) | 160.25 (121.55 - 204.57) | 0.71 (0.64 - 0.77) | 28.26 (17.90 - 42.21) | 33.85 (21.34 - 50.45) | 0.72 (0.65 - 0.78) |
| **Sex** |  |  |  |  |  |  |
| Male | 89.48 (66.84 – 115.87) | 107.95 (81.24 – 139.55) | 0.75 (0.66 – 0.83) | 19.00 (11.82 – 28.11) | 22.95 (14.19 – 33.87) | 0.75 (0.67 – 0.83) |
| Female | 180.15 (138.61 – 229.54) | 213.68 (163.47 – 272.52) | 0.68 (0.61 – 0.74) | 37.78 (23.98 – 56.58) | 44.98 (28.62 – 67.78) | 0.69 (0.63 – 0.75) |
| **Socio-demographic index** |  |  |  |  |  |  |
| High SDI | 293.45 (226.38 - 372.67) | 339.27 (260.52 - 432.89) | 0.57 (0.53 - 0.62) | 61.88 (39.35 - 92.57) | 71.58 (45.82 - 107.51) | 0.58 (0.53 - 0.62) |
| High-middle SDI | 118.41 (88.90 - 152.05) | 168.54 (127.65 - 217.51) | 1.42 (1.27 - 1.56) | 25.01 (15.67 - 37.17) | 35.71 (22.39 - 53.08) | 1.43 (1.29 - 1.57) |
| Middle SDI | 95.75 (71.18 - 123.12) | 140.06 (105.92 - 179.16) | 1.45 (1.38 - 1.51) | 20.27 (12.65 - 30.11) | 29.73 (18.79 - 43.88) | 1.46 (1.39 - 1.52) |
| Low-middle SDI | 93.36 (69.40 - 119.52) | 126.64 (94.37 - 162.05) | 1.22 (1.13 - 1.31) | 19.53 (12.28 - 29.18) | 26.65 (16.77 - 39.42) | 1.24 (1.15 - 1.34) |
| Low SDI | 78.82 (58.08 - 101.32) | 96.16 (71.20 - 123.54) | 0.75 (0.62 - 0.88) | 16.41 (10.22 - 24.45) | 20.19 (12.44 - 29.99) | 0.78 (0.65 - 0.92) |
| **Region** |  |  |  |  |  |  |
| High-income Asia Pacific | 252.70 (194.69 - 322.76) | 303.62 (233.43 - 390.35) | 0.60 (0.55 - 0.66) | 53.68 (33.86 - 80.02) | 64.40 (41.15 - 96.06) | 0.60 (0.54 - 0.65) |
| Central Asia | 121.72 (90.65 - 156.78) | 133.25 (100.45 - 171.27) | 0.55 (0.22 - 0.88) | 25.78 (16.42 - 38.28) | 28.27 (17.77 - 42.15) | 0.54 (0.22 - 0.87) |
| East Asia | 62.01 (45.60 - 81.18) | 111.27 (82.35 - 143.62) | 2.25 (2.15 - 2.36) | 13.19 (8.14 - 19.60) | 23.77 (14.84 - 35.79) | 2.27 (2.16 - 2.38) |
| South Asia | 83.97 (62.10 - 107.74) | 122.55 (91.34 - 156.55) | 1.46 (1.37 - 1.55) | 17.56 (10.91 - 26.17) | 25.80 (16.13 - 38.17) | 1.49 (1.40 - 1.58) |
| Southeast Asia | 76.82 (56.81 - 99.01) | 106.59 (78.98 - 136.33) | 1.13 (1.08 - 1.19) | 16.26 (10.16 - 24.24) | 22.65 (14.06 - 33.74) | 1.15 (1.09 - 1.20) |
| Australasia | 523.64 (403.06 - 665.15) | 692.78 (552.74 - 855.61) | 1.27 (1.16 - 1.38) | 110.17 (68.86 - 163.63) | 145.51 (94.72 - 212.43) | 1.27 (1.16 - 1.39) |
| Caribbean | 195.29 (148.94 - 251.62) | 212.51 (161.70 - 272.65) | 0.43 (0.37 - 0.49) | 41.32 (25.79 - 61.47) | 44.95 (28.32 - 67.63) | 0.43 (0.37 - 0.49) |
| Central Europe | 133.13 (100.04 - 170.46) | 165.51 (125.72 - 212.98) | 1.00 (0.90 - 1.11) | 28.15 (17.51 - 41.99) | 35.07 (22.15 - 52.29) | 1.01 (0.91 - 1.11) |
| Eastern Europe | 157.56 (118.63 - 201.89) | 164.25 (123.16 - 210.83) | 0.33 (0.04 - 0.62) | 33.23 (21.02 - 49.04) | 34.62 (21.77 - 51.59) | 0.32 (0.03 - 0.61) |
| Western Europe | 336.09 (262.20 - 421.77) | 392.61 (302.86 - 500.20) | 0.62 (0.59 - 0.66) | 70.92 (44.94 - 105.84) | 82.87 (52.32 - 124.93) | 0.63 (0.59 - 0.66) |
| Andean Latin America | 248.85 (188.11 - 323.86) | 304.12 (232.83 - 396.59) | 0.78 (0.74 - 0.83) | 52.59 (32.68 - 79.22) | 64.56 (40.40 - 96.37) | 0.80 (0.75 - 0.85) |
| Central Latin America | 210.08 (160.72 - 270.56) | 228.60 (175.29 - 292.84) | 0.30 (0.28 - 0.32) | 44.65 (28.14 - 67.31) | 48.65 (30.74 - 73.01) | 0.31 (0.28 - 0.33) |
| Southern Latin America | 234.50 (180.59 - 300.56) | 295.76 (225.72 - 383.12) | 0.80 (0.75 - 0.85) | 49.53 (31.09 - 72.76) | 62.46 (38.79 - 94.43) | 0.80 (0.74 - 0.85) |
| Tropical Latin America | 185.00 (140.83 - 239.88) | 217.95 (166.87 - 282.66) | 0.63 (0.58 - 0.67) | 38.90 (24.40 - 58.56) | 45.96 (29.15 - 68.53) | 0.64 (0.59 - 0.69) |
| North Africa and Middle East | 141.36 (106.96 - 180.61) | 173.83 (132.55 - 222.48) | 0.90 (0.85 - 0.95) | 29.52 (18.66 - 44.11) | 36.50 (23.02 - 54.30) | 0.92 (0.87 - 0.97) |
| High-income North America | 318.81 (245.07 - 409.11) | 347.88 (265.90 - 447.56) | 0.40 (0.29 - 0.51) | 66.87 (42.30 - 100.93) | 73.08 (46.43 - 110.27) | 0.41 (0.29 - 0.52) |
| Oceania | 77.14 (56.94 - 99.03) | 82.00 (60.53 - 105.84) | 0.08 (0.02 - 0.14) | 16.23 (10.12 - 24.43) | 17.25 (10.42 - 25.92) | 0.08 (0.02 - 0.14) |
| Central Sub-Saharan Africa | 92.75 (69.42 - 119.54) | 92.49 (68.64 - 119.83) | -0.02 (-0.30 - 0.25) | 19.19 (11.88 - 28.61) | 19.35 (11.91 - 28.74) | 0.02 (-0.26 - 0.30) |
| Eastern Sub-Saharan Africa | 77.29 (57.33 - 100.11) | 90.42 (66.87 - 115.97) | 0.64 (0.51 - 0.78) | 16.14 (10.19 - 24.02) | 19.05 (11.87 - 28.48) | 0.68 (0.54 - 0.82) |
| Southern Sub-Saharan Africa | 144.75 (108.08 - 185.98) | 153.15 (114.97 - 196.88) | 0.27 (0.24 - 0.31) | 30.40 (18.99 - 45.73) | 32.08 (20.09 - 47.71) | 0.26 (0.22 - 0.30) |
| Western Sub-Saharan Africa | 95.06 (71.02 - 121.96) | 111.66 (83.10 - 144.59) | 0.79 (0.62 - 0.95) | 19.79 (12.24 - 29.46) | 23.39 (14.77 - 34.79) | 0.81 (0.64 - 0.98) |

DALYs, disability-adjusted life-years; ASR, age-standardized rate; EAPC, estimated annual percentage change; UI, uncertainty interval.
