# Supplementary material for: Trends in the prevalence and disability-adjusted life years of eating disorders from 1990 to 2017: results from the Global Burden of Disease Study 2017
Source: Epidemiol Psychiatr Sci. 2020 Dec 7;29:e191. doi: 10.1017/S2045796020001055 (PMC7737181; doi:10.1017/S2045796020001055)
Supplement: Supplementary file 1 [file S2045796020001055sup001.zip › Supplementary_Table_1.docx]

**Supplementary Table 1. Age-standardized rates of prevalence and disability-adjusted life-years of** **anorexia nervosa in 2017 and their temporal trend from 1990 to 2017 at global and regional levels.**

|  | **Prevalence (95% UI)** | | | **DALYs (95% UI)** | | |
| --- | --- | --- | --- | --- | --- | --- |
|  | **ASR in 1990**  **(per 100 000 population)** | **ASR in 2017**  **(per 100 000 population)** | **EAPC (%)** | **ASR in 1990**  **(per 100 000 population)** | **ASR in 2017**  **(per 100 000 population)** | **EAPC (%)** |
| **Global** | 39.33 (29.86 - 50.13) | 43.87 (33.10 - 56.52) | 0.44 (0.40 - 0.48) | 8.49 (5.29 - 12.55) | 9.51 (5.91 - 14.09) | 0.45 (0.42 - 0.49) |
| **Sex** |  |  |  |  |  |  |
| Male | 15.33 (11.41 – 19.87) | 17.02 (12.65 – 22.04) | 0.40 (0.35 – 0.45) | 3.33 (2.03 – 5.01) | 3.71 (2.27 – 5.56) | 0.41 (0.36 – 0.46) |
| Female | 64.00 (48.93 – 81.61) | 71.49 (54.12 – 92.45) | 0.45 (0.41 – 0.49) | 13.80 (8.65 – 20.23) | 15.48 (9.63 – 22.79) | 0.46 (0.43 – 0.50) |
| **Socio-demographic index** |  |  |  |  |  |  |
| High SDI | 99.54 (75.53 - 126.52) | 111.43 (84.84 - 143.42) | 0.44 (0.38 - 0.49) | 21.7 (13.55 - 31.77) | 24.50 (15.35 - 36.12) | 0.45 (0.39 - 0.52) |
| High-middle SDI | 32.17 (24.08 - 42.09) | 44.60 (32.90 - 58.73) | 1.30 (1.21 - 1.40) | 6.93 (4.19 - 10.40) | 9.68 (5.83 - 14.51) | 1.32 (1.22 - 1.42) |
| Middle SDI | 26.79 (20.07 – 35.00) | 37.25 (27.70 - 48.37) | 1.27 (1.23 - 1.31) | 5.76 (3.51 - 8.61) | 8.06 (4.91 - 12.02) | 1.29 (1.25 - 1.33) |
| Low-middle SDI | 24.42 (18.21 - 31.51) | 31.34 (23.27 - 40.10) | 0.99 (0.90 - 1.08) | 5.18 (3.13 - 7.65) | 6.69 (4.06 – 10.00) | 1.01 (0.93 - 1.10) |
| Low SDI | 21.23 (15.95 - 27.37) | 24.82 (18.53 - 31.80) | 0.57 (0.45 - 0.68) | 4.47 (2.69 - 6.67) | 5.28 (3.25 - 7.90) | 0.6 (0.49 - 0.72) |
| **Region** |  |  |  |  |  |  |
| High-income Asia Pacific | 87.06 (65.86 - 111.84) | 102.45 (77.21 - 132.59) | 0.62 (0.48 - 0.75) | 19.52 (12.24 - 28.67) | 23.78 (15.38 - 34.79) | 0.69 (0.51 - 0.87) |
| Central Asia | 30.17 (22.60 - 38.89) | 32.80 (24.49 - 42.12) | 0.48 (0.20 - 0.76) | 6.54 (4.04 - 9.63) | 7.12 (4.33 - 10.80) | 0.47 (0.20 - 0.75) |
| East Asia | 23.5 (17.21 - 31.36) | 39.44 (28.65 - 53.42) | 2.06 (2.00 - 2.11) | 5.10 (3.10 - 7.72) | 8.64 (5.11 - 13.14) | 2.09 (2.04 - 2.15) |
| South Asia | 22.41 (16.71 - 29.12) | 30.46 (22.71 - 39.11) | 1.15 (1.07 - 1.23) | 4.74 (2.9 - 7.10) | 6.50 (4.02 - 9.66) | 1.18 (1.10 - 1.27) |
| Southeast Asia | 26.51 (19.72 - 34.25) | 35.06 (26.26 - 45.24) | 0.97 (0.91 - 1.03) | 5.68 (3.44 - 8.46) | 7.55 (4.62 - 11.27) | 0.99 (0.93 - 1.04) |
| Australasia | 98.89 (72.48 - 127.2) | 117.43 (88.64 - 152.29) | 0.79 (0.73 - 0.86) | 21.42 (13.41 - 31.99) | 25.23 (15.54 - 38.02) | 0.73 (0.67 - 0.78) |
| Caribbean | 35.69 (26.67 - 45.81) | 38.28 (28.84 - 49.13) | 0.37 (0.31 - 0.42) | 7.72 (4.69 - 11.56) | 8.31 (5.04 - 12.54) | 0.36 (0.31 - 0.41) |
| Central Europe | 32.94 (24.74 - 41.95) | 40.41 (30.27 - 51.76) | 0.94 (0.84 - 1.03) | 7.10 (4.35 - 10.53) | 8.85 (5.42 - 13.29) | 1.02 (0.93 - 1.12) |
| Eastern Europe | 38.30 (28.78 - 49.11) | 40.26 (30.28 - 51.60) | 0.35 (0.09 - 0.61) | 8.32 (5.06 - 12.37) | 8.74 (5.42 - 12.93) | 0.33 (0.06 - 0.59) |
| Western Europe | 120.41 (92.27 - 152.43) | 134.53 (102.65 - 172.92) | 0.43 (0.38 - 0.48) | 26.24 (16.54 - 38.46) | 29.39 (18.47 - 43.55) | 0.43 (0.38 - 0.48) |
| Andean Latin America | 33.13 (24.71 - 42.77) | 38.68 (28.86 - 50.39) | 0.63 (0.53 - 0.73) | 7.15 (4.29 - 10.73) | 8.38 (5.00 - 12.62) | 0.64 (0.54 - 0.74) |
| Central Latin America | 38.67 (29.13 - 49.82) | 42.94 (32.11 - 55.47) | 0.41 (0.39 - 0.44) | 8.36 (5.08 - 12.45) | 9.30 (5.69 - 13.9) | 0.42 (0.39 - 0.44) |
| Southern Latin America | 63.25 (47.55 – 82.00) | 79.46 (58.80 - 103.46) | 0.76 (0.70 - 0.82) | 13.61 (8.01 - 20.55) | 16.92 (10.22 - 25.59) | 0.69 (0.62 - 0.77) |
| Tropical Latin America | 40.78 (30.62 - 52.31) | 46.77 (35.01 - 60.56) | 0.54 (0.50 - 0.58) | 8.73 (5.30 - 12.91) | 10.08 (6.19 - 14.83) | 0.56 (0.52 - 0.60) |
| North Africa and Middle East | 28.32 (21.24 - 36.37) | 32.77 (24.54 - 41.94) | 0.66 (0.60 - 0.72) | 6.02 (3.68 - 8.92) | 7.03 (4.33 - 10.48) | 0.69 (0.63 - 0.75) |
| High-income North America | 107.67 (81.31 - 138.45) | 116.94 (87.42 - 152.30) | 0.34 (0.26 - 0.42) | 23.11 (14.39 - 34.33) | 25.28 (15.76 - 37.69) | 0.37 (0.28 - 0.45) |
| Oceania | 26.00 (19.51 - 33.62) | 27.51 (20.40 - 35.62) | 0.06 (0.01 - 0.13) | 5.56 (3.37 - 8.38) | 5.90 (3.58 - 8.98) | 0.07 (0.01 - 0.14) |
| Central Sub-Saharan Africa | 24.66 (18.31 - 31.66) | 24.69 (18.57 - 31.68) | 0.01 (-0.23 - 0.23) | 5.17 (3.09 - 7.82) | 5.23 (3.12 - 7.84) | 0.04 (-0.19 - 0.28) |
| Eastern Sub-Saharan Africa | 21.28 (15.86 - 27.43) | 24.24 (18.06 - 31.11) | 0.53 (0.41 - 0.64) | 4.51 (2.72 - 6.77) | 5.17 (3.15 - 7.72) | 0.57 (0.45 - 0.68) |
| Southern Sub-Saharan Africa | 35.51 (26.68 - 45.34) | 37.47 (28.13 - 48.19) | 0.26 (0.22 - 0.31) | 7.56 (4.56 - 11.33) | 7.99 (4.83 - 11.92) | 0.27 (0.22 - 0.32) |
| Western Sub-Saharan Africa | 25.85 (19.26 - 33.30) | 29.78 (22.14 - 38.41) | 0.69 (0.53 - 0.85) | 5.47 (3.23 - 8.33) | 6.33 (3.87 - 9.60) | 0.72 (0.56 - 0.88) |

DALYs, disability-adjusted life-years; ASR, age-standardized rate; EAPC, estimated annual percentage change; UI, uncertainty interval.
