# Supplementary figures and images for: Trends in the prevalence and disability-adjusted life years of eating disorders from 1990 to 2017: results from the Global Burden of Disease Study 2017
Source: Epidemiol Psychiatr Sci. 2020 Dec 7;29:e191. doi: 10.1017/S2045796020001055 (PMC7737181; doi:10.1017/S2045796020001055)

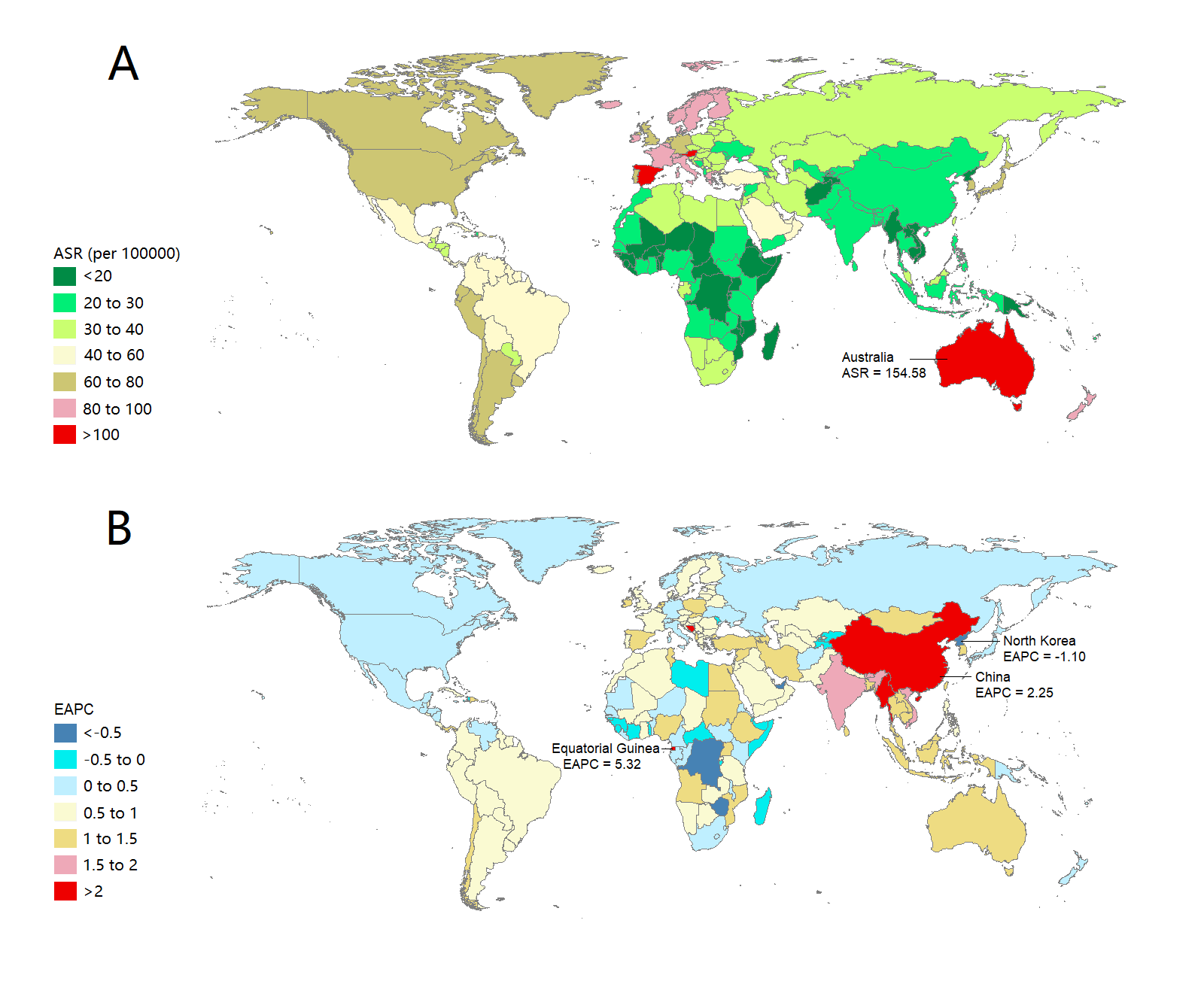

Supplement: Supplementary file 1 [file S2045796020001055sup001.zip › Supplementary_Figure_7.tif]

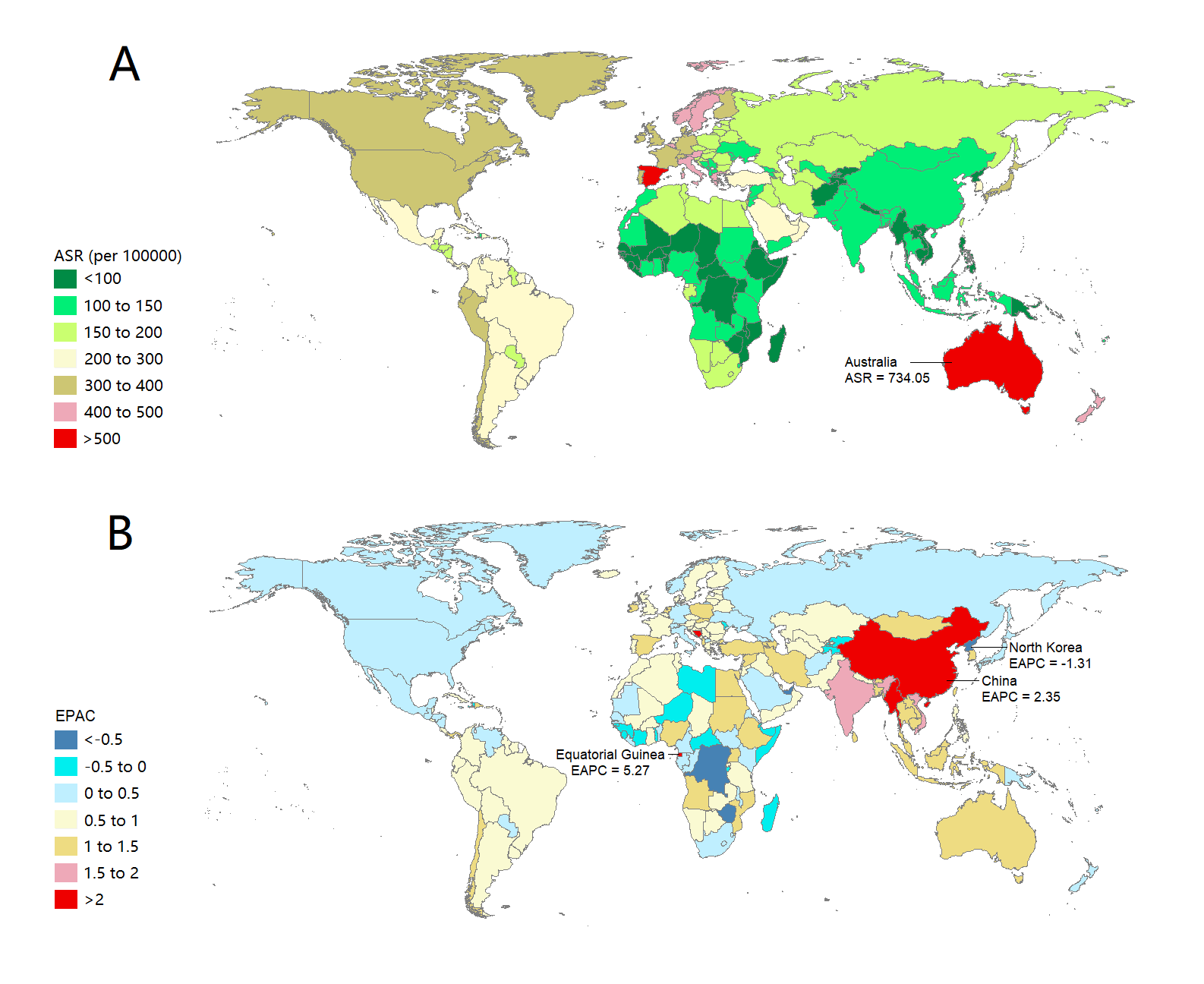

Supplement: Supplementary file 1 [file S2045796020001055sup001.zip › Supplementary_Figure_6.tif]

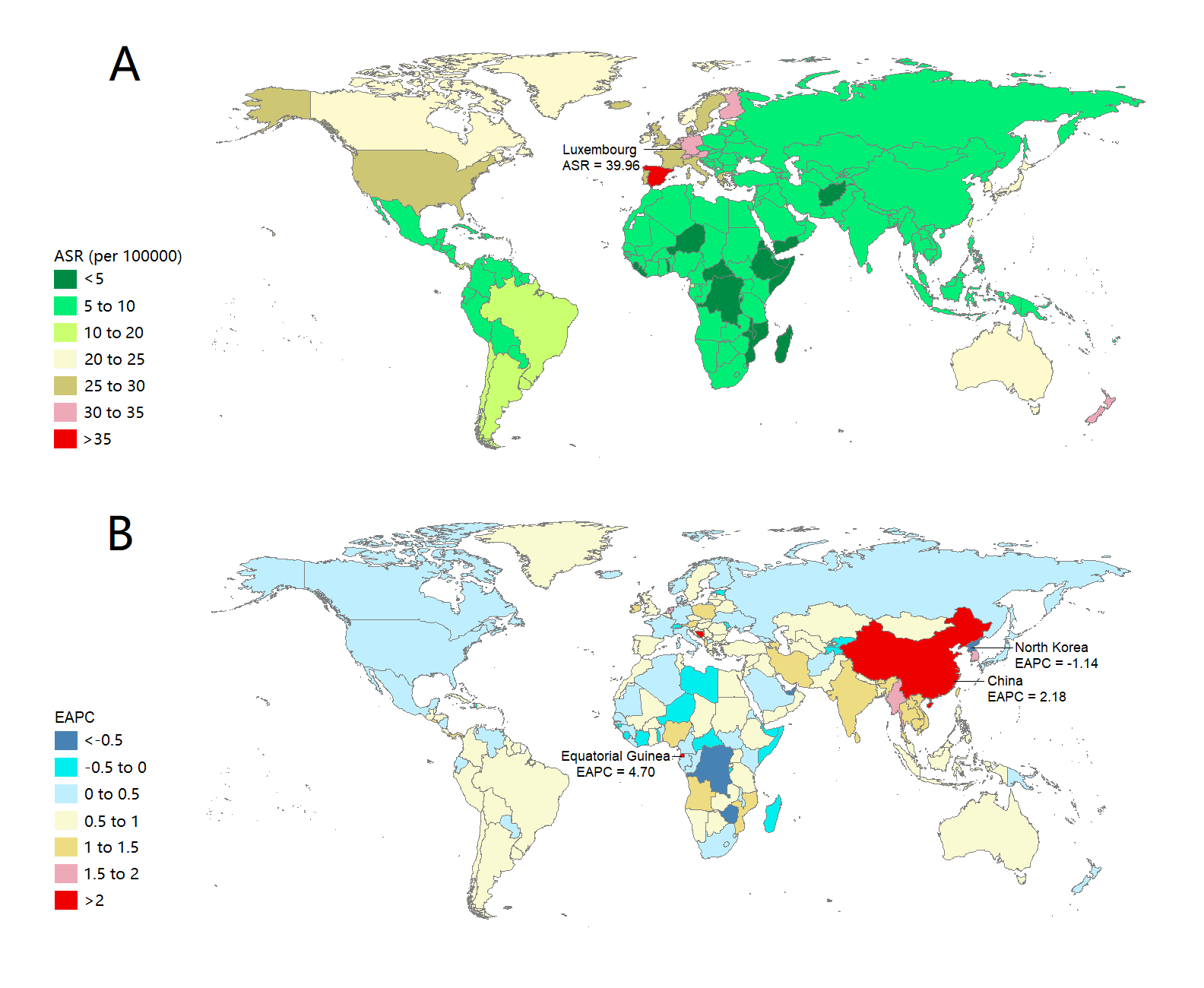

Supplement: Supplementary file 1 [file S2045796020001055sup001.zip › Supplementary_Figure_5.tif]

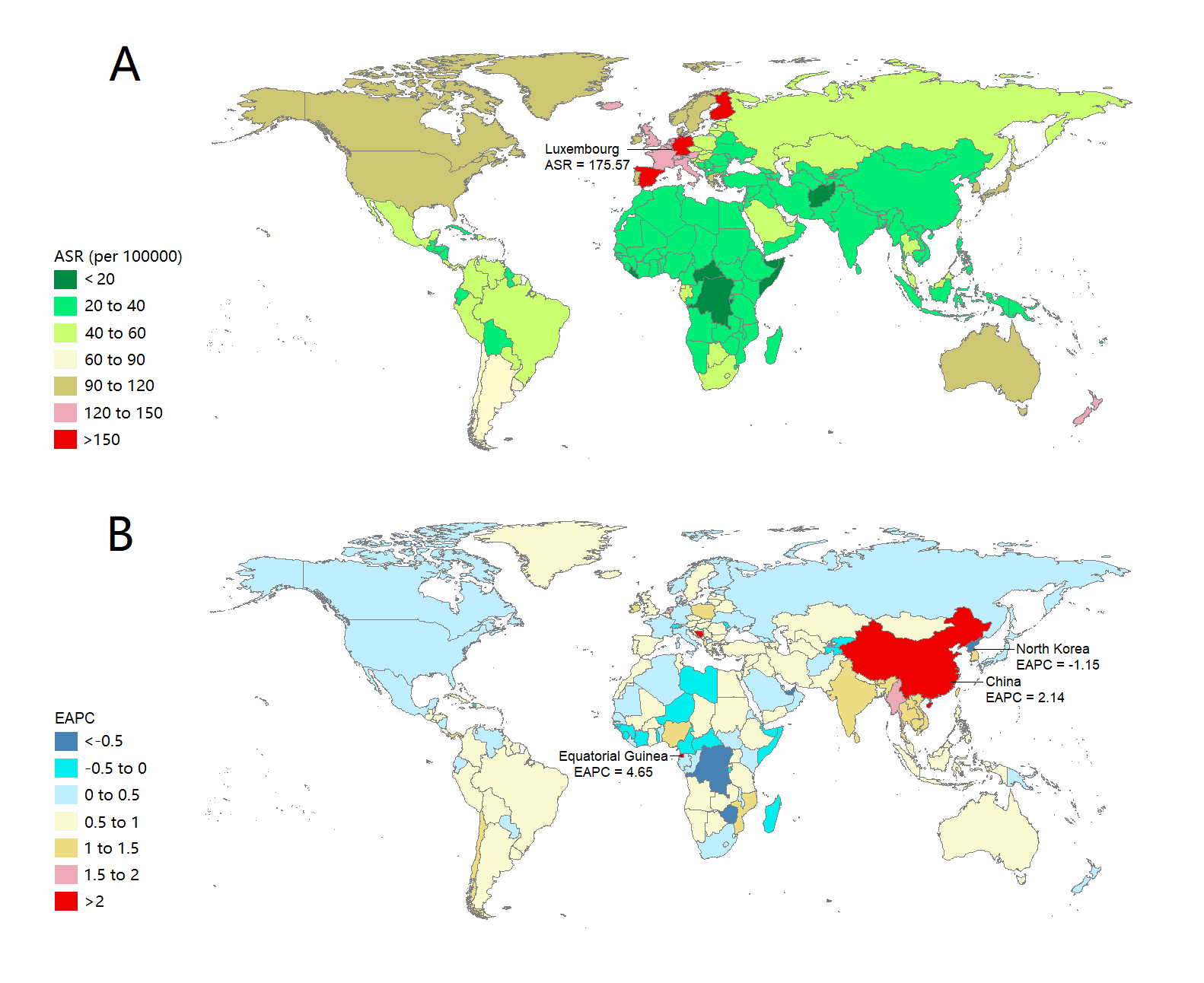

Supplement: Supplementary file 1 [file S2045796020001055sup001.zip › Supplementary_Figure_4.tif]

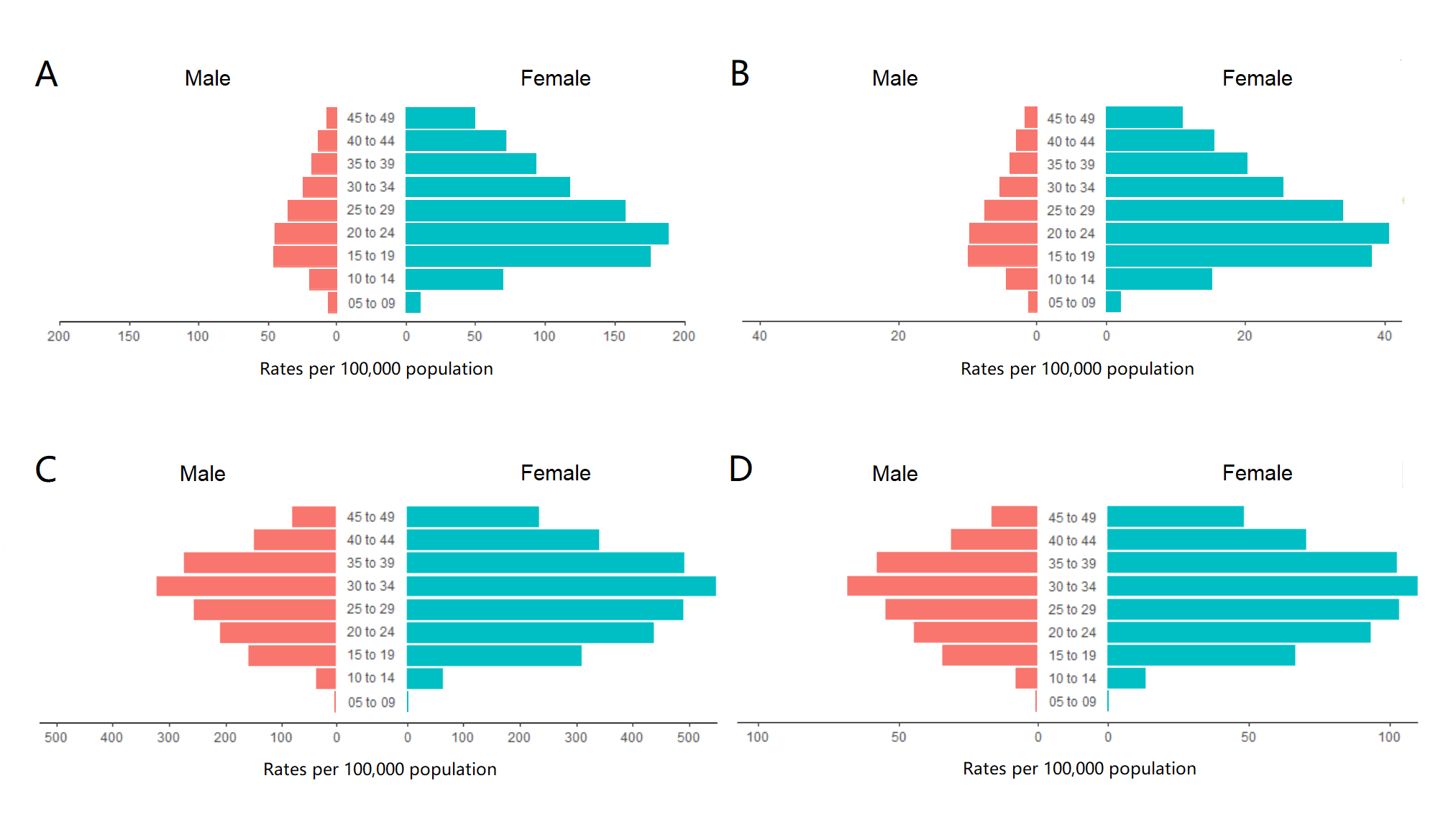

Supplement: Supplementary file 1 [file S2045796020001055sup001.zip › Supplementary_Figure_3.tif]

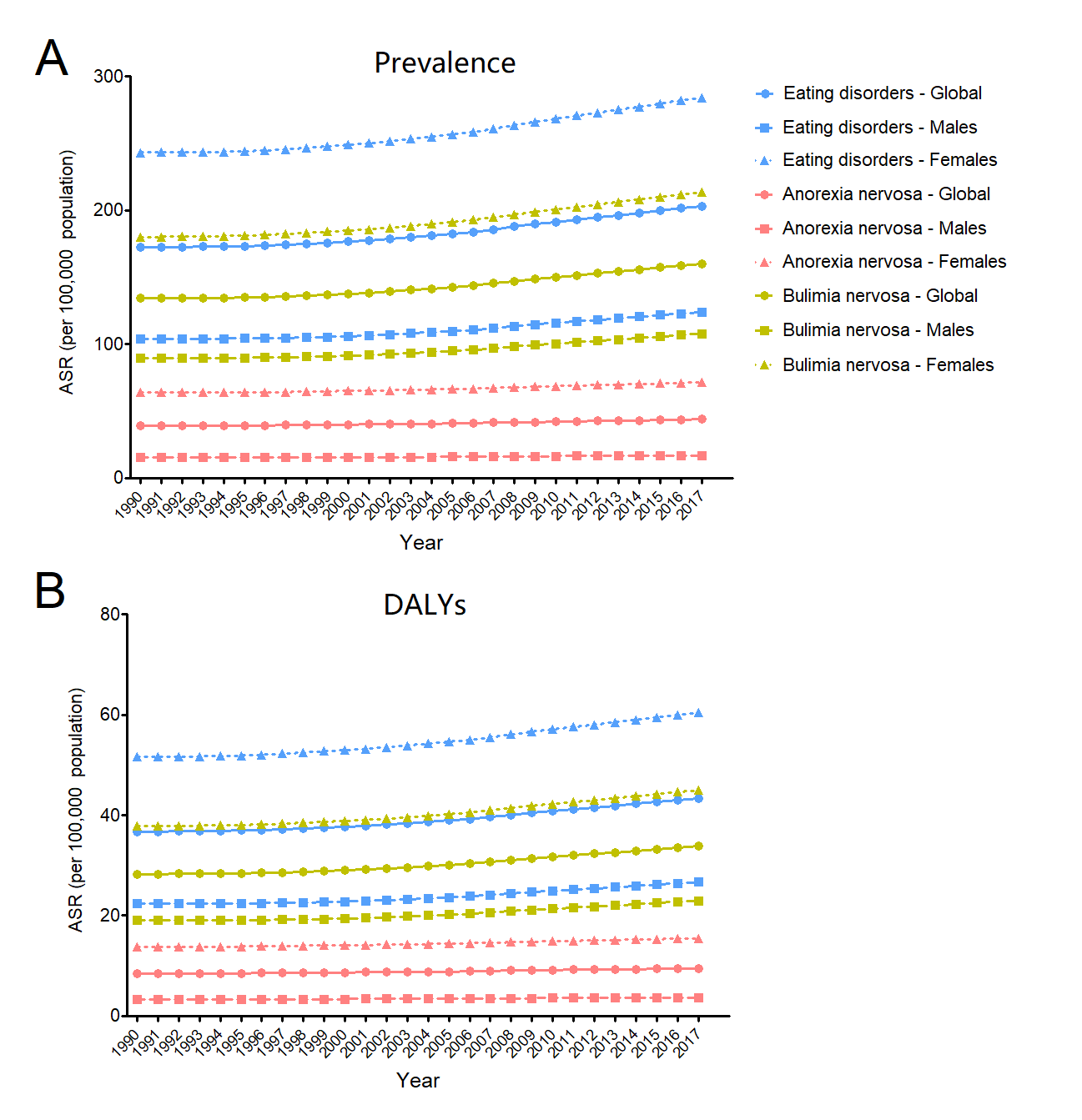

Supplement: Supplementary file 1 [file S2045796020001055sup001.zip › Supplementary_Figure_2.tif]

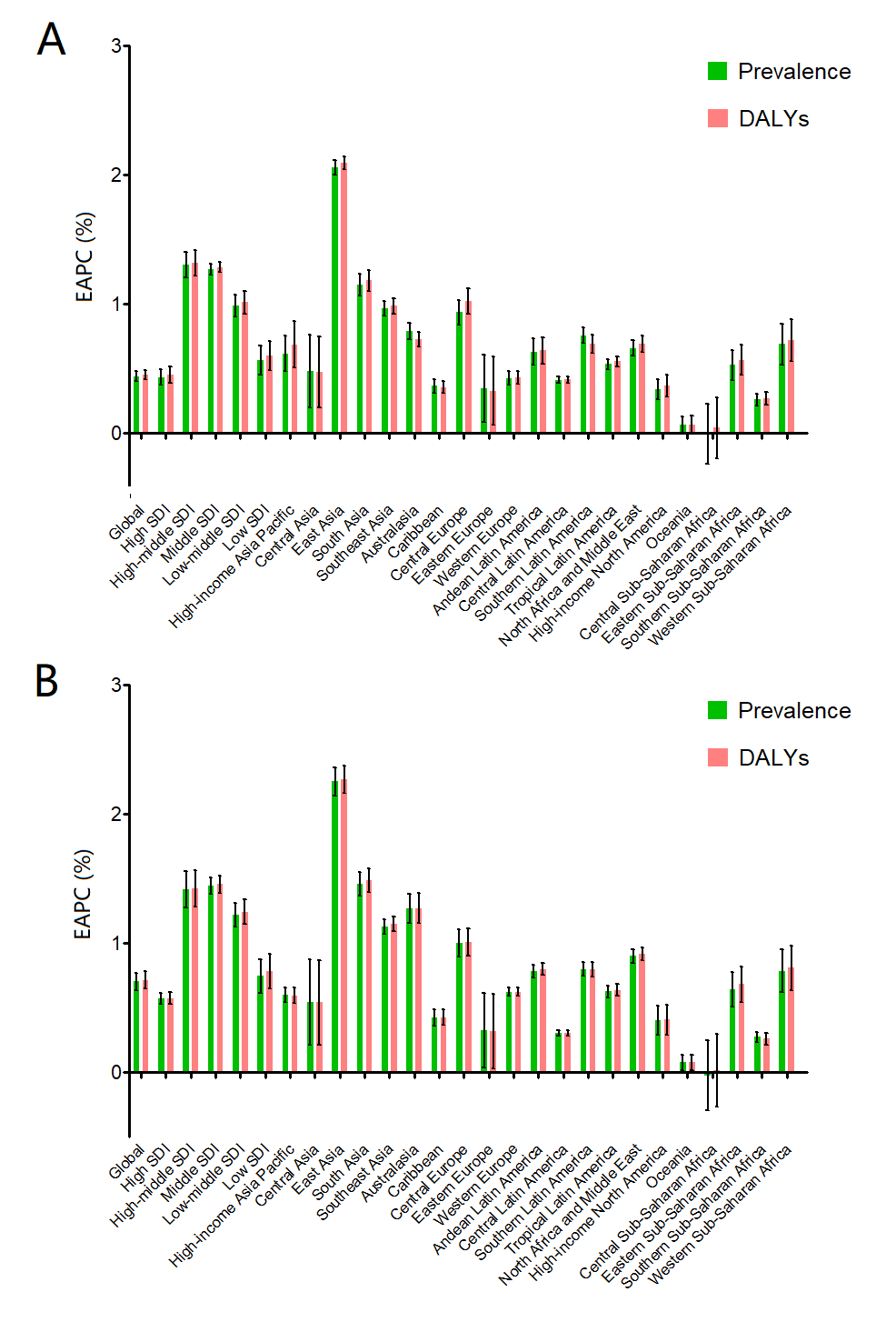

Supplement: Supplementary file 1 [file S2045796020001055sup001.zip › Supplementary_Figure_1.tif]
